# Novel Multi-Target Agents Based on the Privileged Structure of 4-Hydroxy-2-quinolinone

**DOI:** 10.3390/molecules29010190

**Published:** 2023-12-28

**Authors:** Ioanna Kostopoulou, Andromachi Tzani, Konstantina Chronaki, Kyriakos C. Prousis, Eleni Pontiki, Dimitra Hadjiplavlou-Litina, Anastasia Detsi

**Affiliations:** 1 Laboratory of Organic Chemistry, Department of Chemical Sciences, School of Chemical Engineering, National Technical University of Athens, Heroon Polytechniou 9, Zografou Campus, 15780 Athens, Greece; ioanna.th.kostopoulou@gmail.com (I.K.); atzani@mail.ntua.gr (A.T.); konstantina163264@hotmail.gr (K.C.); 2 Institute of Chemical Biology, National Hellenic Research Foundation, 48 Vassileos Constantinou Avenue, 11635 Athens, Greece; kyrprous@eie.gr; 3 Laboratory of Pharmaceutical Chemistry, Faculty of Health Sciences, School of Pharmacy, Aristotle University of Thessaloniki, 54124 Thessaloniki, Greece; epontiki@pharm.auth.gr (E.P.); hadjipav@pharm.auth.gr (D.H.-L.)

**Keywords:** 4-hydroxy-2-quinolinone, carboxamides, antioxidant activity, lipoxygenase inhibition, structure–activity relationships, molecular docking

## Abstract

In this work, the privileged scaffold of 4-hydroxy-2quinolinone is investigated through the synthesis of carboxamides and hybrid derivatives, as well as through their bioactivity evaluation, focusing on the ability of the molecules to inhibit the soybean LOX, as an indication of their anti-inflammatory activity. Twenty-one quinolinone carboxamides, seven novel hybrid compounds consisting of the quinolinone moiety and selected cinnamic or benzoic acid derivatives, as well as three reverse amides are synthesized and classified as multi-target agents according to their LOX inhibitory and antioxidant activity. Among all the synthesized analogues, quinolinone–carboxamide compounds **3h** and **3s**, which are introduced for the first time in the literature, exhibited the best LOX inhibitory activity (IC_50_ = 10 μM). Furthermore, carboxamide **3g** and quinolinone hybrid with acetylated ferulic acid **11e** emerged as multi-target agents, revealing combined antioxidant and LOX inhibitory activity (**3g**: IC_50_ = 27.5 μM for LOX inhibition, 100% inhibition of lipid peroxidation, 67.7% ability to scavenge hydroxyl radicals and 72.4% in the ABTS radical cation decolorization assay; **11e**: IC_50_ = 52 μM for LOX inhibition and 97% inhibition of lipid peroxidation). The in silico docking results revealed that the synthetic carboxamide analogues **3h** and **3s** and NDGA (the reference compound) bind at the same alternative binding site in a similar binding mode.

## 1. Introduction

Nitrogen heterocycles are the main structural unit of a wide variety of natural products, such as DNA and RNA bases, alkaloids, etc., as well as synthetic molecules with potential pharmaceutical, cosmetic, and agrochemical applications. Among the heterocyclic compounds, quinolines and quinolones consist of privileged scaffolds for the development of new drugs. These nitrogen-containing heterocyclic aromatic analogues are present in numerous natural and biologically active compounds. Quinoline and quinolone derivatives possess diverse biological activities and pharmacological properties such as antioxidant, anti-inflammatory, antimalarial, anti-bacterial, antifungal, anti-tubercular, antiviral, anthelmintic, anticonvulsant, and analgesic activity [1,2,3,4,5].

**Figure 1:**
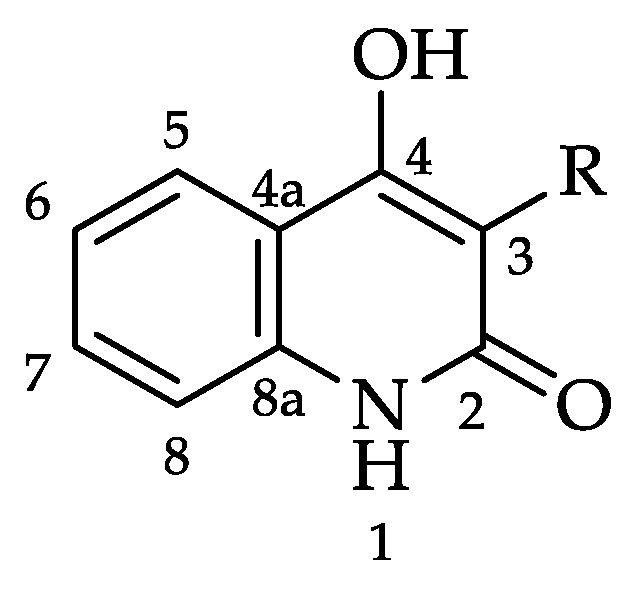
3-Substituted 4-hydroxy-2-quinolinones.

The quinolinone scaffold is one of the most attractive privileged structures for drug discovery research, exhibiting a wide variety of biological activities, such as antibacterial, anti-inflammatory, anticancer, and neuroprotective [6,7,8,9,10,11,12,13]. In particular, compounds possessing 4-hydroxy-2-quinolinone structural features (Figure 1) have gained interest as an important class of heterocyclic molecules with well-known reported activity [14,15,16], while several synthetic strategies have been developed [17,18,19,20].

Driven by the trend of developing new pharmaceutical molecules with improved and multiple properties, research often focuses on combining quinoline, quinolone, or quinolinone structural moieties with amides to form carboxamides. Heterocyclic compounds bearing a substitution on the N-1 position, particularly N-substituted carboxamides, have been extensively examined over the years [21,22,23]. The quinoline-3-carboxamides and their analogues have not only shown significant biological activities (such as antimalarial [24] and antibacterial [25] activity, etc.), but they also could be used in the treatment of auto-immune diseases such as multiple sclerosis, rheumatoid arthritis, systemic lupus erythematosus, and lupus nephritis [26,27,28]. 

**Figure 2:**
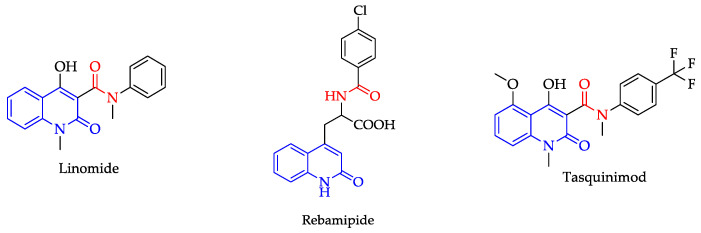
Chemical structures of quinolinone–carboxamide, which are active pharmaceutical ingredients.

Linomide (Figure 2) is a characteristic quinolinone carboxamide, which has been proven to inhibit the process of angiogenesis, being effective against various types of cancers and autoimmune disorders, such as multiple sclerosis, rheumatoid arthritis, systemic lupus erythematosus, and autoimmune encephalomyelitis [29,30]. Rebamipide (Figure 2), a quinolinone amide derivative, is a gastro-protective agent prescribed for the treatment of gastric ulcers and gastritis. Furthermore, it presents antioxidant activity protecting the gastric mucosa against oxygen-derived free radicals [31,32]. Tasquinimod (Figure 2) is a second-generation quinolinone-3-carboxamide; it has been evaluated as 30–60 times more potent anti-tumor agent than linomide, and it has shown antiangiogenic, antitumor, and immune-modulatory properties in preclinical models of prostate cancer and other solid tumors [33,34].

The scientific community’s interest in the aforementioned carboxamide derivatives is not limited and seems to be growing steadily over the years since there is a plethora of information available in the recent literature. 

**Figure 3:**
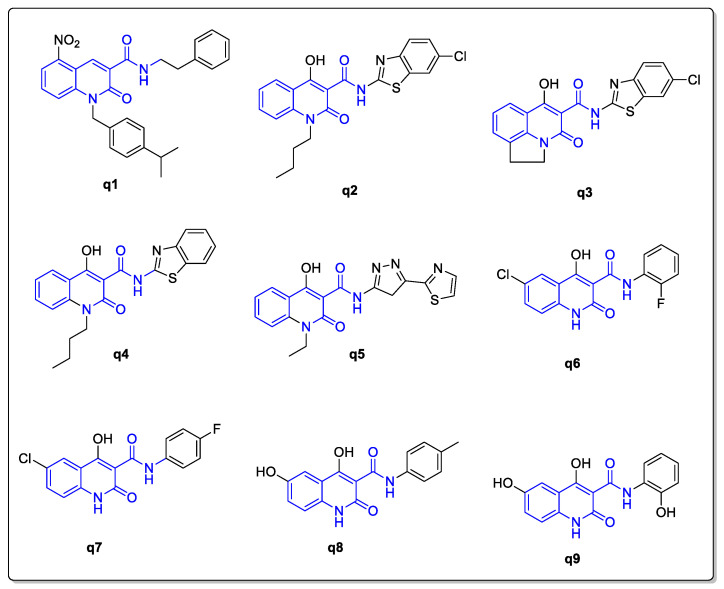
Quinolinone-3-carboxamide derivatives with potent biological activity.

In 2016, Claudia Mugnaini and her research team worked on the design, synthesis, and pharmacological evaluation of 4-quinolone-3-carboxamides and 4-hydroxy-2-quinolone-3-carboxamides as high-affinity cannabinoid receptor 2 (CB2R) ligands. The synthesized derivatives revealed potent activity leading to a novel class of ligands; however, their physicochemical profile should be further examined [35]. In same year, Seung-Hwa Kwak and his group reported a detailed structure–activity relationship analysis of a series of novel quinolinone–carboxamide derivatives and their immunosuppressive effects on IL-2 released from activated T cells. After the optimization of the procedure, the results showed that the synthesized analogues could be used as lead compounds in the design and development of new immunosuppressant agents for treating T cell-mediated immune disorders, while one of them showed significant potency (**q1**; Figure 3) [36]. 

In 2020, Srigouri Huddara and his team introduced 4-hydroxy-2-oxo-1,2-dihydroquinolines as potential inhibitors of *Streptococcus pneumoniae.* In vivo experiments showed acceptable pharmacokinetic profiles for some of the compounds, while the most active one revealed high plasma protein binding. Furthermore, their results revealed three of the compounds as potent to overcome antibiotic resistance (**q2**–**q4**; Figure 3) [37]. In the same year and following the same need for novel antibiotics, Wenjie Xue and his team presented the design, synthesis, biological evaluation, and target identification of N-thiadiazole-4-hydroxy-2-quinolone-3-carboxamides as potential antibacterial agents against *S. aureus*. In vitro and in vivo bioassays demonstrated one of the compounds as the most potent agent (**q5**; Figure 3) [38].

More recently, in 2022, the research team of Sweidan and Sabbah reported the design, synthesis, and biological evaluation of a series of N-phenyl-6-chloro-4-hydroxy-2-quinolone-3-carboxamides and 4,6-dihydroxy-2-quinolone-3-carboxamides. New analogues were examined as anticancer agents and, more specifically, as inhibitors of the phosphatidylinositol3-kinase (PI3Kα), which has emerged as a significant target for the design and development of anticancer drugs. Results demonstrated that two N-phenyl-6-chloro carboxamide analogues (**q6**, **q7**; Figure 3) exerted significant toxicity against human epithelial colorectal adenocarcinoma (Caco-2) and human colon cancer (HCT-116) cell lines. Furthermore, two 4,6-dihydroxy-2-quinolone-3-carboxamide derivatives (**q8**, **q9**; Figure 3) exhibited the most potent cytotoxic effect on breast cancer (MCF-7) and colon cancer (HCT-116) cell lines [39,40].

**Figure 4:**
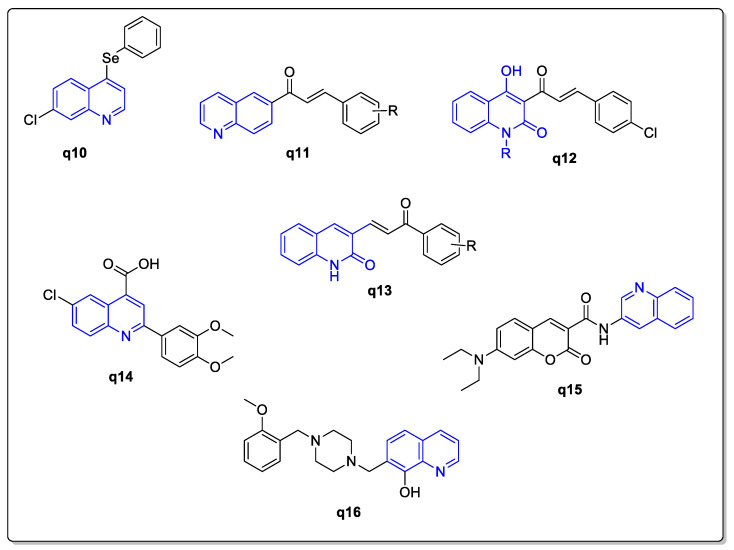
Quinoline and quinolinone derivatives with multi-target activity.

Compounds with multi-target activity have gained researchers’ interest in medicinal chemistry and drug design, bringing on a new era of multi-factorial disease treatment. In particular, the heterocyclic quinoline moiety has been extensively investigated in terms of its ability to offer a multi-target profile in the final drug. A.S. Reis and her team reported for the first time the multi-target activity of 4-phenylselenyl-7-chloroquinoline (**q10**; Figure 4) against anxiety pathology [41]. In 2021, E.M.O.A. Ismail and coworkers published an in silico multi-target approach of several quinoline and quinazoline alkaloids as inhibitors of COVID-19 [42]. Furthermore, in 2020, Mamdouh F.A. Mohamed and his research group published a review study reporting several quinoline–chalcone hybrids (**q11**–**q13**; Figure 4) as potential multi-target anticancer agents [43]. However, the majority of the references in the literature present quinoline derivatives or hybrids as multi-target compounds for neurodegeneration, particularly against key targets in Alzheimer’s disease (**q14**–**q16**; Figure 4) [44,45,46,47,48]. 

Since chronic inflammation and oxidative stress are two commonly associated conditions involved in the pathophysiology of cancer, diabetes, cardiovascular pulmonary diseases, and others, the development of novel drugs behaving as multi-target compounds could offer better treatment [49,50,51,52]. 

Lipoxygenases (LOXs) are a heterogeneous family of structurally related non-heme iron-containing enzymes that catalyze the oxidation of polyunsaturated fatty acids (PUFA) (such as linoleic or arachidonic acid) to produce hydroperoxides. They are widely spread in plants, fungi, and animals, while they are classified into several types of LOXs according to their selectivity to oxygenate fatty acids in a specific position [53,54]. 5-Lipoxygenase (5-LOX) is directly related to human diseases, and its mechanism of expression has been extensively studied. 5-LOX catalyzes two steps in the biosynthesis of leukotrienes (LTs), a group of bioactive lipid mediators of inflammation derived from arachidonic acid. LTs are involved in many inflammatory and allergic disorders, while novel studies of molecular and cellular biology showed the implication of 5-lipoxygenase in diseases like cancer, osteoporosis, and heart attack [55,56,57].

Oxidative stress caused by the excessive production of reactive oxygen species (ROSs) highly contributes to the pathophysiology of various diseases. ROSs, like superoxide radical anion, hydrogen peroxide, and hydroxyl radical, are produced during the inflammation process by phagocytic leukocytes (e.g., neutrophils, monocytes, macrophages, and eosinophils) that invade the tissue. Since the rate of ROS production is increased in most pathophysiological conditions, developing novel drugs that combine anti-inflammatory and antioxidant activity could be beneficial for treating several diseases [58,59,60,61].

Our research group has been previously involved with synthesizing several N-substituted-4-hydroxy-2-quinolinones and quinolinone–carboxamides to investigate their potential dual-acting role as antioxidant and anti-inflammatory agents. Derivatives with an appealing pharmacological profile have been identified from this research [62,63,64,65,66]. 

In this project, the design, synthesis, and bioactivity evaluation of three classes of different compounds, which share the structural framework of 4-hydroxy-2-quinolinone as a common feature, are presented. More specifically, herein we report the synthesis of twenty-two quinolinone-3-carboxamides (**3a**–**3u**, **7**), from which, to our knowledge, derivatives **3a**–**3e**, **3g**–**3k**, **3n**–**3s**, **3u,** and **7** are introduced for the first time in the literature; seven novel quinolinone–carboxamide and cinnamic or benzoic acid hybrids (**11a**–**11g**); and three reverse amides (**16a**–**16c**), from which **16b** and **16c** analogues are new.

## 2. Results and Discussion

### 2.1. Chemistry

**Scheme 1:**
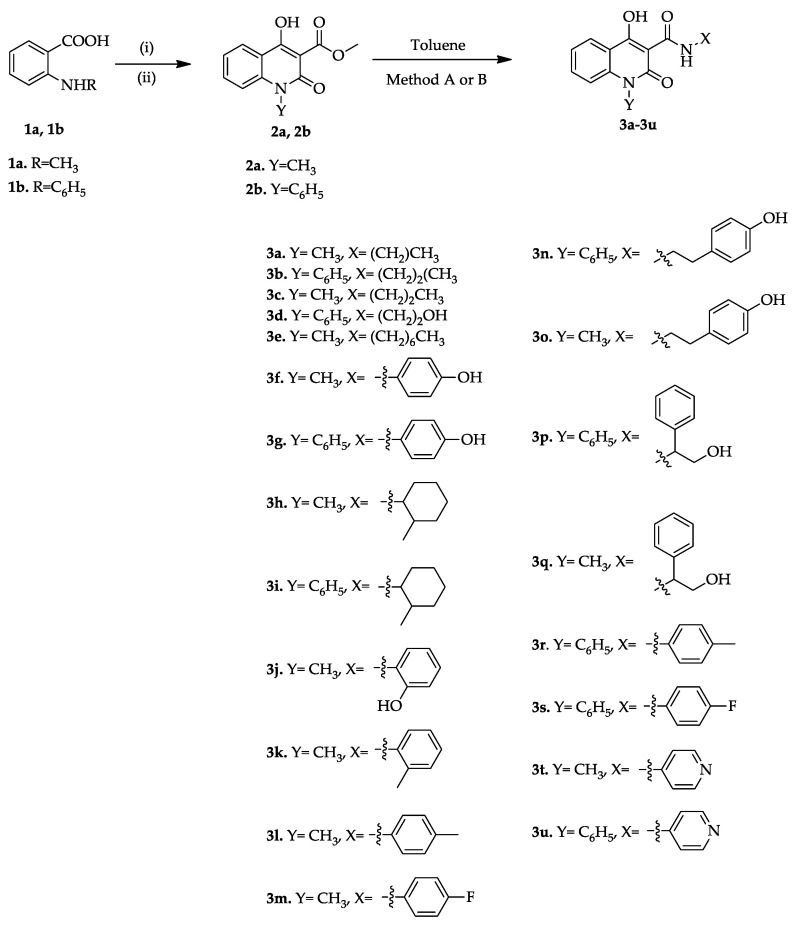
Synthetic strategy for the preparation of the desired quinolinone-3-carboxamides **3a-3u**. Reagents and conditions: (i) HOBt, DCC, THF, 0 °C, (ii) NaH, dimethymalonate, and THF, r.t. [26].

In the first series of the synthesized compounds, quinolinone–carboxamides **3a**–**3u** were prepared following the synthetic strategy depicted in Scheme 1. More specifically, the synthesis of the N-substituted-3-carboxymethyl quinolinone intermediates **2a** and **2b** was accomplished using the corresponding commercially available anthranilic acids (**1a** and **1b**) as the starting materials, following our previously reported two-step methodology [26]. In this concept, the carboxylic acid group of compounds **1a** and **1b** was transformed into the non-isolated active benzotriazolyl ester using N-hydroxybenzotriazole (HOBt) and dicyclohexylcarbodiimide (DCC), which was then reacted with dimethymalonate sodium salt through a C-acylation reaction. The afforded enolate intermediates were then cyclized to obtain the preferred 3-ethoxycarbonyl-4-hydroxyquinolin-2-ones **2a** and **2b**. The final amide formation was furnished by heating the corresponding quinolinone esters with an equimolar amount of the appropriate amine or aniline, using toluene as solvent.

**Scheme 2:**
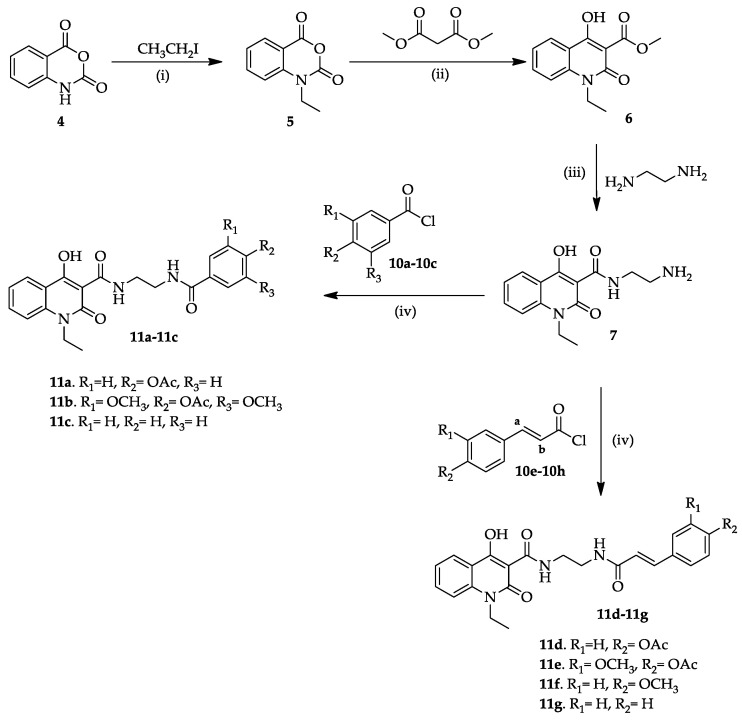
Synthesis of novel quinolinone–phenolic acid hybrids (**11a**–**11g**). Reagents and conditions: (i) ethyl iodide, NaH, r.t.; (ii) dimethyl malonate, NaH, DMF, 80 °C; (iii) ethylenediamine toluene, 80 °C; (iv) phenolic acid chloride (**10a**–**10c**, **10e**–**10h**), THF, Et_3_N; 50 °C.

The second series of compounds consists of hybrid molecules (**11a**–**11g**), which combine the framework of 4-hydroxy-2-quinolinone with selected substituted benzoic or cinnamic acid motifs connected through a di-amide linker (Scheme 2). More specifically, the key intermediate nitrogen heterocycle, N-ethyl-3-methoxycarbonyl-4-hydroxy-2-quinolinone (**6**) was synthesized through a two-step procedure starting from isatoic anhydride **4**, which was alkylated using ethyl iodide in the presence of sodium hydride in dimethylformamide (DMF), to yield compound **5**. Quinolinone derivative **6** was then synthesized through a C-acylation reaction of dimethyl malonate, using N-ethyl isatoic anhydride **5** as an acylating agent in sodium hydride, resulting in a 35% yield. The reaction of quinolinone **6** with ethylenediamine formed the amino carboxamide **7**. The desired hybrids (**11a**–**11g**) were synthesized via amidation reaction of the carboxamide **7** with either various acetyloxy benzoic acid chlorides (**10a**–**10c**) or acetyloxy- or methoxy substituted cinnamic acid chlorides (**10e**–**10h**), using triethylamine (Et_3_N) as the base in tetrahydrofuran (THF) at 50 °C. These final hybrid diamides were isolated in moderate to high yields (56% to 90%).

**Scheme 3:**
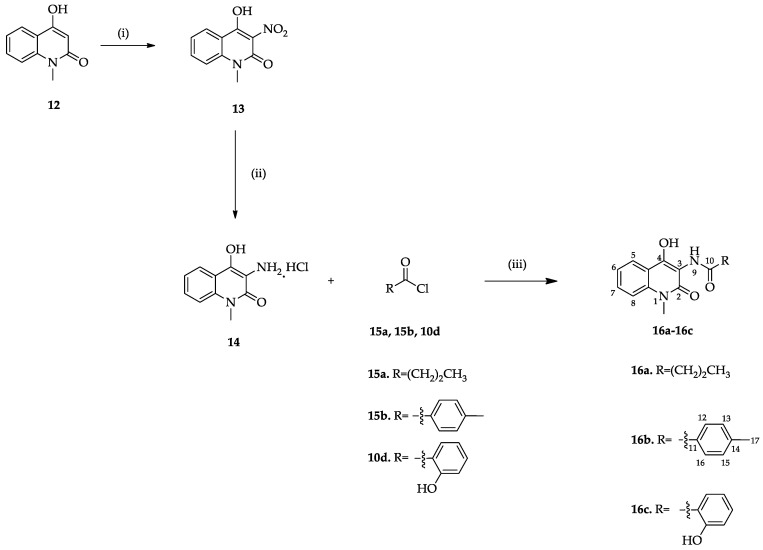
Synthesis of quinolinone–reverse carboxamides **16a**–**16c**. Reagents and conditions: (i) HNO_3_, NaNO_2_, CH_3_COOH; (ii) aq. NaOH 1M, Na_2_S_2_O_4_; (iii) Et_3_N, THF, 50 °C.

Furthermore, three quinolinone-reverse amides (**16a**–**16c**) were synthesized (Scheme 3). More specifically, the commercially available starting material, 4-hydroxy-1-methyl-quinolin-2(1H)-one (**12**), was transformed to its 3-nitro analogue **13** through a nitration reaction, using 70% HNO_3_ and NaNO_2_ in CH_3_COOH under heating at 90 °C. Reduction of the nitro group was carried out in an alkaline environment of aqueous solution NaOH 1M using sodium dithionite as the reducing agent, and after acidification, the hydrochloric salt of 3-amino-4-hydroxy-2-quinolinone (**14**) was obtained. The desired reverse carboxamides (**16a**–**16c**) were obtained through an acylation reaction between quinolinone **14** and the corresponding acid chloride **15a**–**15c** in moderate yields of 30–46%. Butyryl chloride (**15a**) and 4-methyl-benzoyl chloride (**15b**) were commercially available, while 2-hydroxy-benzoyl chloride (**10d**) was synthesized according to the protocol described in the Experimental Section.

The structural characterization of the synthesized compounds was confirmed by analytical means such as ^1^H and ^13^C-NMR and mass spectrometry (MS). 

The ^1^H-NMR spectra of all the synthesized carboxamides **3a**–**3u** and **7**, as well as the ones of the hybrids **11a**–**11g,** are characterized by a signal at a very low field, between 16 and 17 ppm. This signal is attributed to the proton of the 4-OH group of the quinolinone moiety, which is deshielded as a result of taking part in a strong intramolecular hydrogen bond with the neighboring carbonyl group of the amide bond. The signal of the 4-OH group in the ^1^H NMR spectra of the synthesized reverse amides **16a**–**16c** appears between 12 and 13 ppm, owing to the fact that the intramolecular hydrogen bond with the carbonyl group is weaker as the carbonyl group is located further than in the case of the carboxamides **3a**–**3u** and **7**.

### 2.2. Bioactivity Assays 

In this work, the carboxamides **3a**–**3u**, the quinolinone–cinnamic or benzoic acid hybrids **11a**–**11g** and the reverse amides **16a**–**16c** were tested for their antioxidant activity via five in vitro techniques; (i) the interaction with the stable free radical 2,2-diphenyl-1-picrylhydrazyl (DPPH); (ii) the inhibition of lipid peroxidation; (iii) the hydroxyl radical scavenging ability; (iv) the 2,2-azino-bis(3-ethylbenzothiazoline-6-sulfonic acid (ABTS) radical cation (ABTS**·**^+^) reduction; and (v) the superoxide anion radical scavenging ability [61,67,68,69,70].

Furthermore, all the synthesized compounds were examined as inhibitors of soybean lipoxygenase (LOX), which is a plant enzyme with satisfactory homology to the human 5-LOX, and the results can be qualitatively considered as an indication of their anti-inflammatory activity. For this study, the UV absorbance-based soybean LOX assay was used [67,68,69,71,72,73]. 

#### 2.2.1. DPPH Assay 

The results of the DPPH scavenging ability of all the synthesized compounds are presented in Table 1, while nordihydroguaiaretic acid (NDGA) is used as the reference compound. 

The majority of the tested carboxamides **3a**–**3u** showed a weak activity, while this result was not time-dependent (from 20 to 60 min reaction). Compounds **3f** and **3g**, which possess a p-phenolic group at position 3 of the heterocyclic ring, exhibited the best activity in this assay (48.6% and 51.7%, respectively, in a 60 min interaction), confirming the above statement. Changing the position of the hydroxyl group from the p- to the o-position of the aromatic ring led to the derivative **3j** with analogous activity (46.5% in a 60 min interaction with the radical). Replacement of the hydroxyl group with a methyl (analogues **3k**, **3l**, and **3r**) or a fluoro (analogues **3m** and **3s**) substituent led to inactive compounds, regardless of the substituent on the heterocyclic nitrogen. 

The evaluation of the antioxidant activity of the quinolinone hybrid analogues **11a**–**11g** via the DPPH in vitro technique showed that they do not possess any activity (0–5.0%). Furthermore, the synthesized compounds **16a**–**16c** also showed weak activity in this assay, while it seems that the reverse amide bond does not enhance the antioxidant activity of the molecules. This can be verified in the case of analogues **3j** and **16c**: the carboxamide **3j** showed 46.5% DPPH radical inhibitory activity, whereas the reverse amide **16c** showed lower activity (28.0%). 

Overall, the results of the DPPH method revealed that the antioxidant activity of the tested compounds is not depended on the substituent, which is attached to the nitrogen of the quinolinone moiety, while it is mostly related to the presence or not, of phenolic hydroxyl groups, which can react directly with the free radical. Although all the tested compounds possess a hydroxyl group at position 4 of the heterocyclic moiety, this OH cannot effectively interact with the DPPH radical as it is involved in a strong hydrogen bond with the adjacent carbonyl oxygen. This observation is in accordance with our previous studies on analogous structures [64,65].

#### 2.2.2. Lipid Peroxidation of Linoleic Acid Induced by AAPH Radical

As far as the ability of the tested compounds to inhibit lipid peroxidation of linoleic acid induced by a thermal free radical producer (AAPH) is concerned, the majority of the analogues were found to be potent inhibitors showing activity equal to or even higher than the reference compound Trolox (Table 1). 

Among all the synthesized carboxamides (**3a**–**3u**), derivatives **3a**–**3c**, **3f**, **3g**, **3n**–**3q**, and **3t** showed the best activity, with a 100% inhibition value. More specifically, N-methyl and N-phenyl analogues **3a** and **3b**, which possess a propyl substituent attached to the amide group, exhibited the best activity. The elongation of the aliphatic chain of the amide to seven carbon atoms, in the case of **3a** analogue, led to the inactive **3e** derivative. 

Carboxamide **3c**, possessing an N-methyl substituent at the quinolinone framework and a hydroxyethyl group attached to the amide bond, is one of the most potent inhibitors (100% inhibition). Replacement of the N-methyl substituent with an N-phenyl one (carboxamide **3d**) resulted in a weak antioxidant agent (32.8%). 

N-methyl and N-phenyl carboxamides **3f** and **3g**, which are derived by 4-amino-phenol, exhibited 100% inhibition. The shift of the hydroxyl group to the o-position of the aromatic ring converted the potent **3f** agent to a weak inhibitor (analogue **3j**, 32.8% inhibition). Furthermore, the replacement of the hydroxyl group with a methyl (analogues **3l** and **3r**) or a fluoro (analogues **3m** and **3s**) substituent led to inactive or very weak antioxidant compounds (0% to 28.9% inhibition). 

Overall, it seems that the antioxidant activity via the AAPH in vitro assay is equally affected by the group attached to the amide bond and the N-substituent. However, the presence of a p-phenolic group at the amide part always results in a potent inhibitor, regardless of the substituent attached to the heterocyclic nitrogen (carboxamides **3f**, **3g**, **3n**, and **3o**).

As far as the hybrid compounds that share the structural features of quinolinone and cinnamic or benzoic acid derivatives are concerned, results revealed that all the synthesized and tested analogues (**11a**–**11g**) were strong inhibitors of the lipid peroxidation of linoleic acid, exhibiting higher activity than the reference compound Trolox (88.0%). However, it seems that the cinnamic acid derivatives **11d** and **11g** showed a slightly better activity (94.0% and 97.0%, respectively) than their benzoic acid analogues **11a** and **11c** (89.0% and 91.0%, respectively). 

The evaluation of the antioxidant activity of the reverse amides **16a**–**16c** via this method led to satisfactory activity (83.0% inhibition of lipid peroxidation for the **16a** analogue and 88.0% for the **16b** and **16c** analogues). Furthermore, in the case of **3j** and **16c** analogues, it seems that the insertion of the reverse amide moiety enhanced the inhibitory activity from 32.8% (carboxamide **3j**) to 88.0% (reverse amide **16c**).

#### 2.2.3. Competition with DMSO for Hydroxyl Radicals

The results of the antioxidant activity of carboxamide derivatives **3a**–**3u** in terms of their ability to scavenge hydroxyl radicals are presented in Table 2.

Caboxamides **3a**–**3u** were selected to be tested for the competition with DMSO for hydroxyl radicals assay (Table 2). Analogue **3j**, possessing an N-methyl substituent and an o-phenolic group at position 3 of the heterocyclic ring, showed the best activity (100%). Changing the position of the hydroxyl group from the o- to the p- position of the aromatic ring leads to compounds with lower interaction with the OH radical, regardless of the substituent on the heterocyclic nitrogen atom (analogues **3f** and **3g**, 67.7%). The presence of the hydroxyl group is crucial for the activity, as proven by replacing the hydroxyl group of carboxamide 3j with a methyl one (carboxamide 3k), which resulted in a totally inactive compound. 

Furthermore, carboxamides **3m** and **3s**, which are both derived from the p-fluoro-aniline, exhibited significant antioxidant activity (94.0% and 100%, respectively). Replacement of the fluoro substituent by a hydroxyl group led to compounds **3f** and **3g**, which showed a moderate activity (67.7%), while replacement by a methyl substituent led to carboxamides **3l** and **3r** with lower activity (53.7% and 50.7%, respectively). Finally, a pyridine group attached to the amide bond resulted in inactive (compound 3t) or weakly active (compound **3u**) antioxidants. 

Overall, it seems that the ability of the tested carboxamide derivatives to scavenge hydroxyl radicals was mostly related to the amide substitution, while it was less affected by the N-substituent of the quinolinone moiety. The only case in which this observation was not verified refers to the **3p** and **3q** analogues, which carry the same amide part; however, they presented an extremely opposite activity. The N-phenyl derivative **3p** exhibited potent activity (98.0%), while the corresponding N-methyl analogue **3q** showed very weak interaction with the hydroxyl radical (16.9%).

#### 2.2.4. ABTS Radical Cation Decolorization Assay

In the ABTS radical cation (ABTS+) decolorization assay, carboxamide derivatives **3a**–**3u** were examined, and the results are presented in Table 2. Compounds **3f** and **3g**, which are both derived from 4-amino-phenol, showed the best activity with 77.3% and 72.4% values, respectively. Replacement of the aromatic ring by an aliphatic chain of two carbons led to the corresponding N-methyl and N-phenyl derivatives **3c** and **3d**, which are inactive (25.6% and had no activity, respectively). 

#### 2.2.5. Superoxide Anion Radical Scavenging Ability

All the synthesized carboxamides **3a**–**3u** were examined in terms of their ability to scavenge superoxide anion radicals, and the results are presented in Table 2. N-phenyl carboxamide derivative **3b**, which possesses an aliphatic chain of three carbons attached to the amide bond, revealed the best antioxidant activity (84.6%), whereas its N-methyl derivative **3a** exhibited very weak activity (23.1%). The insertion of a longer aliphatic chain as a substituent in the case of the N-methyl derivative **3f** resulted in an enhanced scavenging ability (66.6%). Carboxamides **3c** and **3d**, which both possess a hydroxyethyl group, exhibited very satisfactory activity (76.9% and 66.6%, respectively) (Supplementary Materials).

#### 2.2.6. Soybean LOX Inhibitory Activity

Results obtained from the evaluation of the LOX inhibitory activity of the synthesized and tested compounds are presented in Table 3. 

Among all the examined carboxamides, analogues **3h** and **3s** exhibited the best lipoxygenase inhibitory activity, with IC_50_ = 10 μM. Carboxamide **3h** possesses an N-methyl substituent to the quinolinone moiety and a 2-methyl-cyclohexane group attached to the amide bond. Replacement of the N-methyl substituent with an N-phenyl one led to the inactive derivative **3i**. Moreover, the replacement of the alicyclic 2-methyl-cyclohexane substituent by an aliphatic one with the same number of carbon atoms (carboxamide **3e**) was detrimental for the LOX inhibitory activity as **3e** showed no activity. 

N-phenyl carboxamide **3s** possesses a 4-fluoro-phenyl group attached to the amide bond. Replacement of the N-phenyl substituent by an N-methyl one resulted in the weak inhibitor **3m** (15% at 0.1 mM). Keeping the N-phenyl substituent and replacing the fluoro group by a hydroxyl one (**3g** analogue) led to a better inhibitor (IC_50_ = 27.5 μM), while replacement by a methyl group led to an inactive agent (**3r** analogue, 37.5% at 100 μM). 

Furthermore, the N-methyl analogue **3f** and the N-phenyl analogue **3g**, which both share a phenolic group as a common substituent attached to the amide bond, were also evaluated as potent LOX inhibitors, with IC_50_ = 49.0 μM and 27.5 μM, respectively. Replacement of the aromatic group with an aliphatic chain of two carbons resulted in the less potent N-methyl derivative **3c** (IC_50_ = 52.0 μM) and the inactive N-phenyl derivative **3d**. 

N-Methyl derivatives **3a** and **3l** were characterized as good inhibitors (IC_50_ = 60.0 μM and 45.0 μM, respectively), while their N-phenyl analogues **3b** and **3r** exhibited very weak inhibitory activity (45% at 0.1 mM and 37.4% at 0.1 mM, respectively). N-methyl and N-phenyl pyridine derivatives **3t** and **3u** were inactive against the soybean lipoxygenase enzyme. 

Among all the synthesized and tested quinolinone–cinnamic or benzoic acid hybrids, the acetyloxy-ferulic acid derivative **11e** exhibited the best LOX inhibitory activity, with IC_50_ = 52.5 μM. Moreover, the 4-methoxy-cinnamic acid derivative **11f** showed satisfying activity (IC_50_ = 70.0 μM), while the cinnamic acid derivative **11g** presented a slightly weaker activity (IC_50_ = 85.5 μM). The 4-acetyloxy-cinnamic acid derivative was evaluated as an inactive agent (5.0% at 100 μM). Overall, it seems that the combined presence of the methoxy and acetyloxy groups (case of **11e** hybrid) enhances the LOX inhibitory activity, while the presence of each group separately is not favorable for the activity (cases of **11d** and **11f** analogues). 

In addition, comparing the cinnamic acid analogues **11g** (IC_50_ = 85.5 μM) and **11d** (5.0% at 100 μM), with their corresponding benzoic acid derivatives **11c** (IC_50_ = 57.0 μM) and **11a** (IC_50_ = 61.0 μM), it seems that the absence of the unsaturated system seems to be favorable for LOX inhibitory activity.

As far as the LOX inhibitory activity of the reverse amides is concerned, analogue **16a** emerged as the most potent (IC_50_ = 81.0 μM) among this subgroup. Comparison of the reverse analogue **16c** with its amide derivative **3j**, shows that the reverse bond slightly enhances the LOX inhibitory activity of the compounds (IC_50_ = 100 μM for the **3j** analogue and IC_50_ = 82.5 μM for the **16c** analogue).

### 2.3. Computational Studies–Docking Simulation Soybean Lipoxygenase

#### Docking Studies of the Synthesized Derivatives in Soybean LOX

All the synthesized derivatives were studied in silico. For the docking studies, soybean lipoxygenase-1 (PDB: 3PZW) was selected in order to be in accordance with the biological assay. As already mentioned, lipoxygenases are hyperoxidases catalyzing the oxygenation of free and esterified polyunsaturated fatty acids to hydroperoxides. Based on recent findings, apart from the substrate-binding site (iron-binding site), lipoxygenases present additional potential allosteric binding sites [64,74]. First of all, molecular docking was carried out for all the synthesized derivatives, setting a catalytic pocket around the iron with no promising results. Aiming to explore the potential binding mode of the novel derivatives in a detailed manner, blind docking to the whole protein was accomplished to encompass all the potential binding sites. Additionally, docking studies were carried out to NDGA (IC_50_ = 0.45 μM), a strong LOX inhibitor standard, used herein as a reference compound for comparison purposes. The docking studies revealed that compounds interact with the soybean LOX-1 through allosteric interactions.

**Figure 5:**
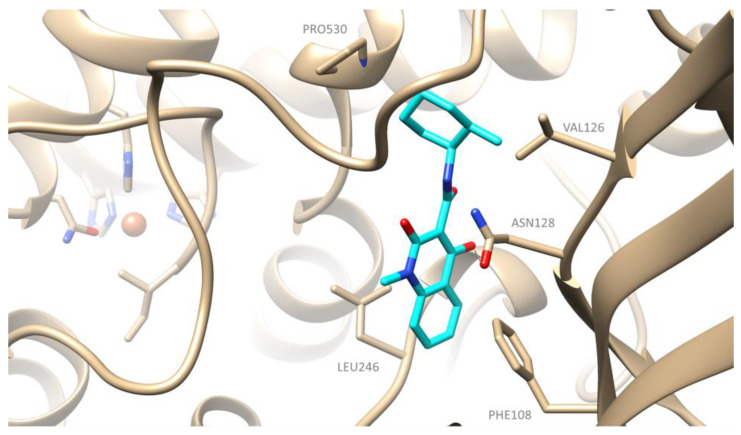
The 3D preferred docking pose of **3h** (depicted in cyan) bound to soybean lipoxygenase (ID: 3PZW). Nitrogen appears in blue colour while oxygen in red. The one hydrogen bond is illustrated with dashed grey lines. Iron is depicted as an orange sphere.

**Figure 6:**
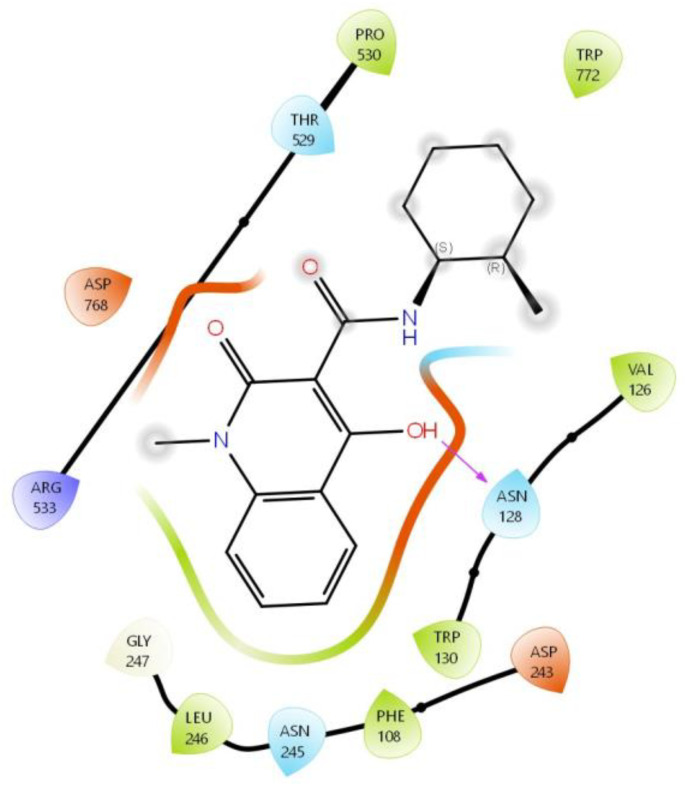
Ligand interaction diagram of compound **3h** soybean lipoxygenase (ID: 3PZW). The hydrogen bond interaction with residues is illustrated by a purple dashed arrow. The hydrogen bond interaction with residues is illustrated by a purple dashed arrow. The hydrophobic residues are shown in green, the polar ones in cyan, the positively charged in blue, and the negatively charged in red. The Figure was made with free Maestro (Free Maestro academic license, Version 13.8—**Schrödinger Release 2023-4**: Maestro, Schrödinger, LLC, New York, NY, USA, 2023) [75].

**Figure 7:**
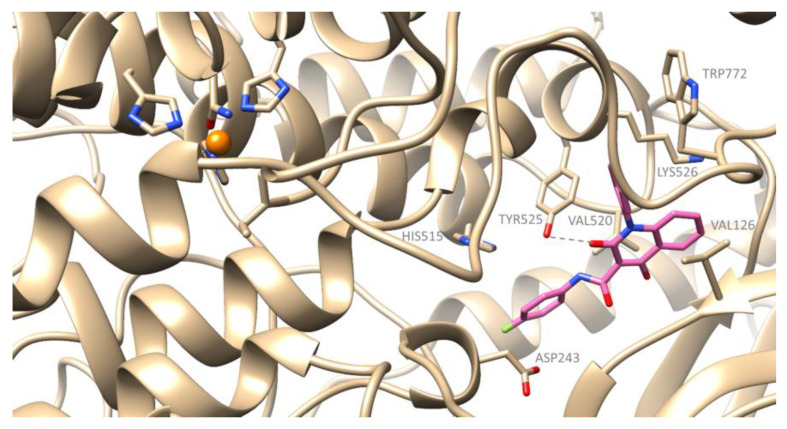
The 3D preferred docking pose of **3s** (depicted in pink) bound to soybean lipoxygenase (ID: 3PZW). Light green colour corresponds to fluorine, blue to nitrogen and red to oxygen. The one hydrogen bond is illustrated with dashed grey lines. Iron is depicted as an orange sphere.

**Figure 8:**
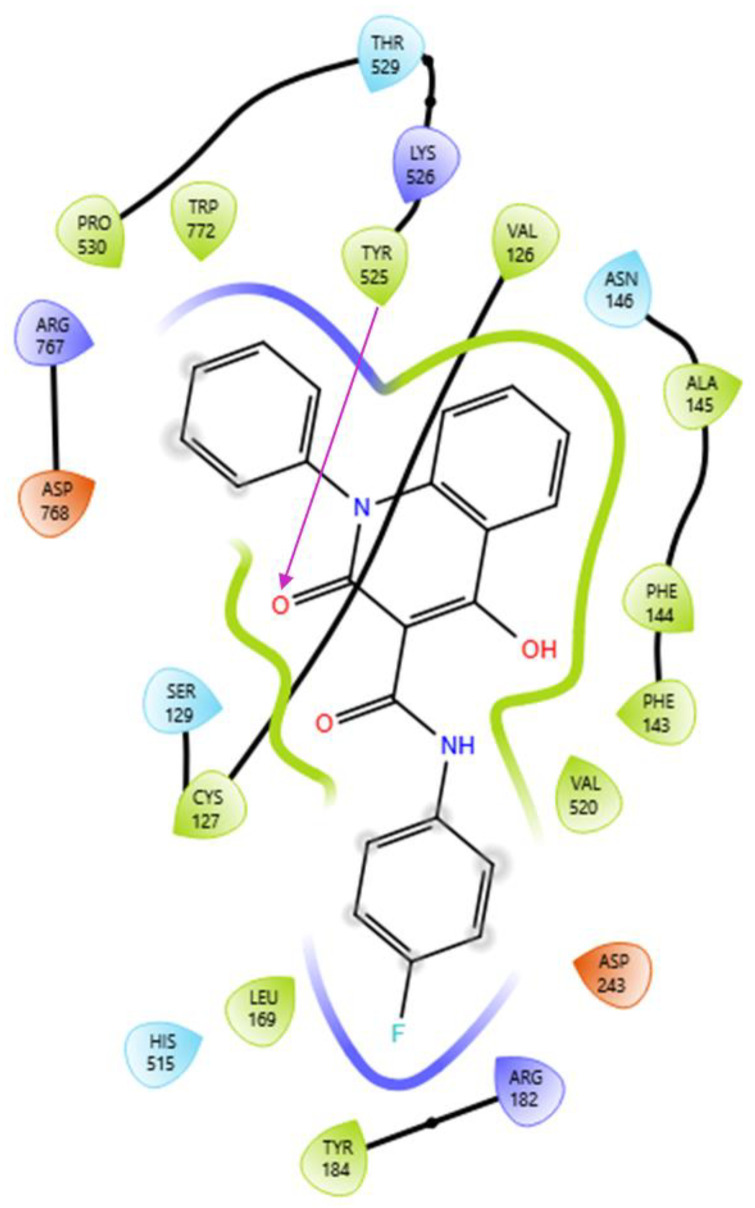
Ligand interaction diagram of compound **3s** to soybean lipoxygenase (ID: 3PZW). The hydrogen bond interaction with residues is illustrated by a purple dashed arrow. The hydrophobic residues are shown in green, the polar ones in cyan, the positively charged in blue, and the negatively charged in red. Figure made with free Maestro (Free Maestro academic license-Version 13.8—**Schrödinger Release 2023-4**: Maestro, Schrödinger, LLC, New York, NY, USA, 2023) [75].

**Figure 9:**
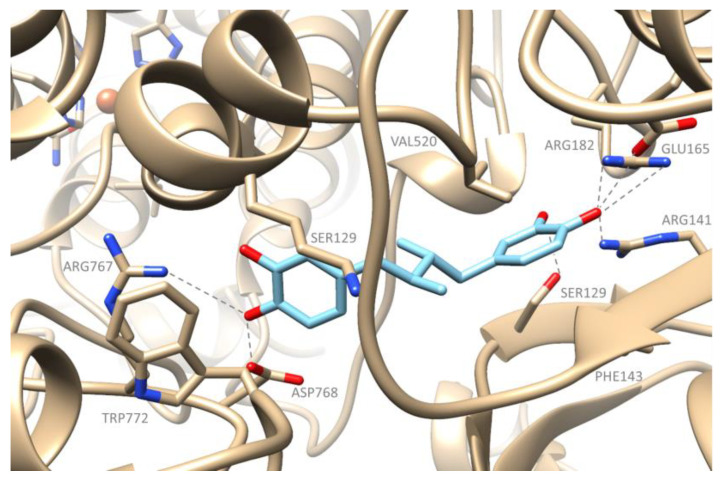
The 3D preferred docking pose of NDGA (depicted in light blue) bound to soybean lipoxygenase (ID: 3PZW). Blue colour corresponds to nitrogen and red to oxygen. Hydrogen bonds are illustrated with dashed grey lines. Iron is depicted as an orange sphere.

**Figure 10:**
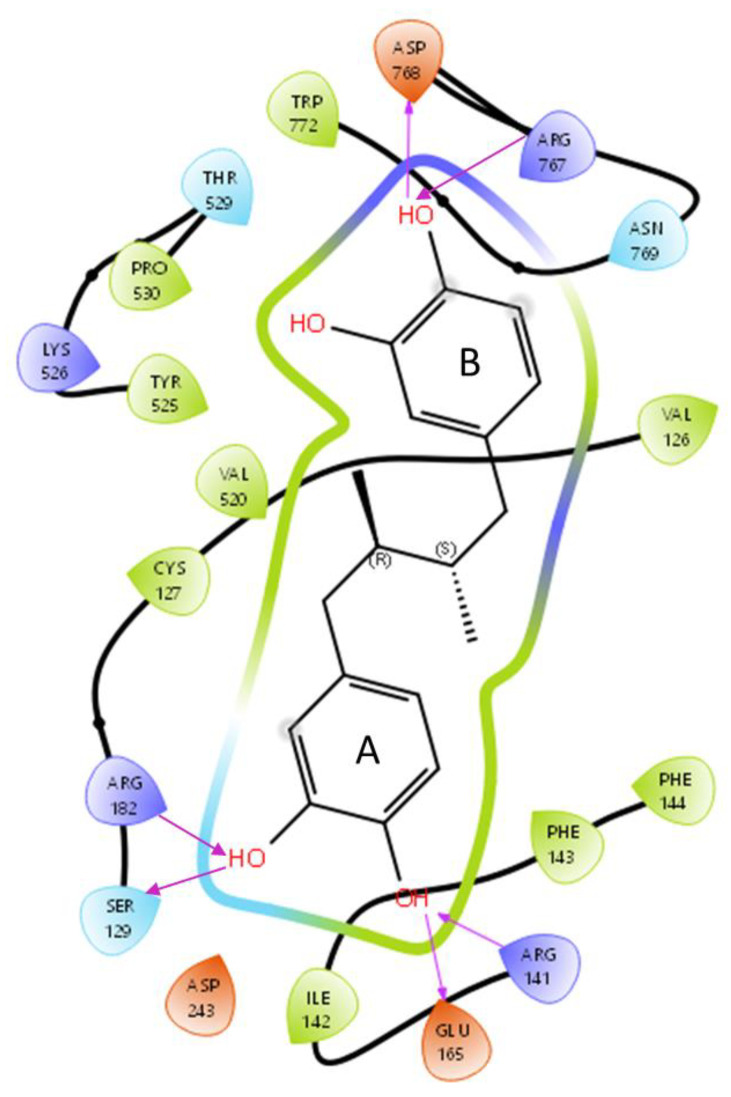
Ligand interaction diagram of NDGA soybean lipoxygenase (ID: 3PZW). The hydrogen bond interaction with residues is illustrated by a purple dashed arrow. Figure made with free Maestro (Free Maestro academic license-Version 13.8—**Schrödinger Release 2023-4**: Maestro, Schrödinger, LLC, New York, NY, USA, 2023) [75].

The most biologically active derivatives were further investigated, including a visual examination of intermolecular interactions with soybean LOX (PDB ID: 3PZW) (**3h**; Figure 5 and Figure 6 and **3s**; Figure 7 and Figure 8, respectively). Compound **3h** had an AutoDockVina score of −8.8 kcal/mol while **3s** −8.4 kcal/mol binding to soybean LOX (PDB ID: 3PZW). It is well known that a one-to-one correlation is difficult to reach between the obtained results from the in vitro inhibition of soybean lipoxygenase that represents experimental values and docking scores that are based on algorithms and scoring function calculations. Docking describes the preferred orientation of the ligand bound to the protein. Compound **3h** presents hydrophobic interactions with Phe108, Val126, Asn128, Leu246, and Pro530 and a hydrogen bond with Asn128, while compound **3s** presents hydrophobic interactions with Val126, Asp243, Val520, Lys526, and Trp772, a hydrogen bond with Tyr525 and π-cation interactions with His515. Moreover, NDGA forms hydrogen bonds between the –OH groups of ring A with Ser129, Arg141, Arg142, and Glu165 and hydrogen bonds between the –OH groups of ring B with Arg767 and Asp 768 (Figure 9 and Figure 10). Additionally, it develops hydrophobic interactions with residues Phe143, Val520, Lys526, and Trp772, resembling the hydrophobic binding motif of **3s**. It is well known that most LOX inhibitors act as antioxidants or by scavenging free radicals, oxidizing the enzyme via a carbon-centered radical on a lipid chain. It is possible that compounds **3h** and **3s** extend into the hydrophobic domain and block the substrates to the binding site, thus preventing oxidation [69].

## 3. Materials and Methods

NMR spectroscopy: synthesized compounds were structurally elucidated using Varian Gemini 300 MHz (Palo Alto, CA, USA) at the School of Chemical Engineering, NTUA and Varian 600 MHz (Palo Alto, CA, USA) at the National Hellenic Research Foundation, NMR spectrometers using DMSO-d_6_ and CDCl_3_ 99.9 atom % D.

Melting points were determined on a Gallenkamp MFB-595 melting point apparatus (London, UK) and are uncorrected. High-resolution mass spectra were obtained on an ultra-high-pressure liquid chromatography mass spectrometer mass accuracy and ultra-high resolution (UHPLC—LTQ Orbitrap Velos, Thermo Scientific, Waltham, MA, USA). Mass spectra were obtained on an ESI-MS (HPLC-LCQ Fleet/Thermo Scientific). For the in vitro tests, a Lambda 20 (Perkin–Elmer, Norwalk, CT, USA) UV–Vis double beam spectrophotometer was used. Soybean lipoxygenase, sodium linoleate, 2,2-azobis-(2-amidinopropane) dihydrochloride (AAPH), and DPPH were obtained from Sigma Chemical, Co. (St. Louis, MO, USA).

All commercially available starting materials were used without further purification. Commercially available tetrahydrofuran (THF) was dried prior to use by refluxing over Na. All other solvents (puriss. quality) were used without further purification. 

Column chromatography was performed with silica gel 60.

### 3.1. Synthesis and General Procedures

#### 3.1.1. General Procedure for the Synthesis of Quinolinone–Carboxamides **3a–3x**

1. Method A:

Equimolar amounts of 1-methyl-4-hydroxy-2-oxo-1,2-dihydro-quinoline-3-carboxylic acid methyl ester (**2a**)**,** or 1-phenyl-4-hydroxy-2-oxo-1,2-dihydro-quinoline-3-carboxylic acid methyl ester (**2b**) (1.07 mmol), and the appropriate amine (1.07 mmol) were added to 5 mL of dry toluene. The reaction mixture was refluxed for 24 h, while the reaction was monitored by thin-layer chromatography (TLC). After the completion of the reaction, the mixture was diluted with CH_2_Cl_2_ and washed with saturated aqueous NaHCO_3_. The organic layer was collected, dried with anhydrous sodium sulfate (Na_2_SO_4_), and evaporated in vacuo.

2. Method B:

Equimolar amounts of 1-methyl-4-hydroxy-2-oxo-1,2-dihydro-quinoline-3-carboxylic acid methyl ester (quinolinone **2a**) or 1-phenyl-4-hydroxy-2-oxo-1,2-dihydro-quinoline-3-carboxylic acid methyl ester (quinolinone **2b**) (1.07 mmol) and the appropriate amine (1.07 mmol) were added to 5mL of dry toluene. The reaction mixture was refluxed for 24 h, while the reaction was monitored by TLC. After the completion of the reaction, the product precipitated after cooling at ambient temperature, the solid was filtered off and washed with diethylether (Et_2_O) or hexane.

*4-hydroxy-1-methyl-3-propylquinolin-2(1H)-one* (**3a**); The compound was synthesized according to the general procedure (Method A), starting from quinolinone **2a** (250 mg, 1.07 mmol) and propylamine (63 mg, 1.07 mmol). After the work-up procedure, the product was obtained upon recrystallization from methanol as a colorless powder. Yield: 65%; ^1^H NMR (300 MHz, CDCl_3_) *δ* 17.29 (s, 1H, -OH), 10.33 (s, 1H, -NH), 8.23 (dd, *J* = 8.1, 1.8 Hz, 1H, Ar-H), 7.68 (ddd, *J* = 8.6, 7.2, 1.5 Hz, 1H, Ar-H), 7.36 (d, *J* = 8.7 Hz, 1H, Ar-H), 7.30 (ddd, *J* = 8.0, 7.2, 1.2 Hz, 1H, Ar-H), 3.69 (s, 3H, N-CH_3_), 3.42 (m, 2H, -NHCH_2_-), 1.68 (m, 2H, -CH_2_CH_3_), 1.02 (t, *J* = 7.2 Hz, 3H, -CH_2_CH_3_); ^13^C NMR (75 MHz, CDCl_3_) δ 172.2, 171.2, 163.0, 140.0, 133.8, 125.7, 122.5, 116.6, 114.3, 96.9, 41.0, 29.3, 22.8, and 11.7; HR-MS *m*/*z* (pos): 260.1161 C_14_H_16_O_3_N_2_ (calcd. 261.1234).

*4-hydroxy-2-oxo-1-phenyl-N-propyl-1,2-dihydroquinoline-3-carboxamide* (**3b**); the compound was synthesized according to the general procedure (Method A) starting from quinolinone **2b** (316 mg, 1.07 mmol) and propylamine (63 mg, 1.07 mmol). After the work-up procedure, the product was obtained as a colorless powder without any further purification. Yield: 62%; ^1^H NMR (300 MHz, CDCl_3_) *δ* 17.53 (s, 1H, -OH), 10.11 (s, 1H, -NH), 8.23 (dd, *J =* 7.8, 1.2 Hz, 1H, Ar-H), 7.58 (m, 3H, Ar-H), 7.44 (t, *J =* 8.0 Hz, 1H, Ar-H), 7.26 (m, 3H, Ar-H), 6.62 (d, *J =* 8.7 Hz, 1H, Ar-H), 3.37 (m, 2H, -NHCH_2_-), 1.61 (m, 2H, -CH_2_CH_3_), 0.96 (t, *J =* 7.5 Hz, 3H, -CH_2_CH_3_); ^13^C NMR (75 MHz, CDCl_3_) *δ* 173.0, 171.0, 163.1, 140.8, 137.2, 133.1, 130.3, 129.1, 129.0, 125.2, 122.6, 116.2, 116.0, 96.7, 40.9, 22.5, and 11.6; HR-MS *m*/*z* (pos): 322.1317 C_19_H_18_O_3_N_2_ (calcd. 323.1390).

*4-hydroxy-N-(2-hydroxyethyl)-1-methyl-2-oxo-1,2-dihydroquinoline-3-carboxamide* (**3c**); the compound was synthesized according to the general procedure (Method A) starting from quinolinone **2a** (250 mg, 1.07 mmol) and ethanolamine (65 mg, 1.07 mmol). After the work-up procedure, the product was obtained pure after flash column chromatography (dichloromethane:methanol/98:2) as a colorless powder. Yield: 60%; ^1^H NMR (300 MHz, CDCl_3_) δ 16.75 (s, 1H, -OH), 10.60 (s, 1H, -NH), 8.19 (dd, *J =* 7.8, 1.5 Hz, 1H, Ar-H), 7.69 (ddd, *J =* 8.6, 7.2, 1.5 Hz, 1H, Ar-H), 7.35 (d, *J =* 8.4 Hz, 1H, Ar-H), 7.30 (t, *J =* 7.8 Hz, 1H, Ar-H), 5.30 (s, 1H, -CH_2_OH), 3.86 (m, 2H, -CH_2_OH), 3.68 (s, 3H, N-CH_3_), 3.64 (m, 2H, -NHCH_2_-); ^13^C NMR (75 MHz, CDCl_3_) δ 172.1, 172.0, 162.8, 140.1, 134.0, 125.7, 122.6, 116.3, 114.4, 97.0, 62.4, 42.2, and 29.3; HR-MS *m*/*z* (pos): 262.0954 C_13_H_14_O_4_N_2_ (calcd. 263.1026).

*4-hydroxy-N-(2-hydroxyethyl)-2-oxo-1-phenyl-1,2-dihydroquinoline-3-carboxamide* (**3d**); The compound was synthesized according to the general procedure (Method B) starting from quinolinone **2b** (316 mg, 1.07 mmol) and ethylenediamine (64 mg, 1.07 mmol) after 2 h stirring at 110 °C. After the work-up procedure, the product was obtained upon recrystallization from methanol as white transparent crystals. Yield: 73%; ^1^H NMR (300 MHz, CDCl_3_) *δ* 17.00 (s, 1H, -OH), 10.40 (s, 1H, -NH), 8.24 (dd, *J* = 8.1, 1.5 Hz, 1H, Ar-H), 7.58 (m, 3H, Ar-H), 7.46 (ddd, *J* = 8.6, 7.2, 1.5 Hz, 1H, Ar-H), 7.27 (m, 3H, Ar-H), 6.65 (d, *J* = 8.4 Hz, 1H, Ar-H), 3.79 (m, 2H, -CH_2_OH), 3.59 (m, 2H, -NHCH_2_-), 2.41 (brs, 1H, -CH_2_OH); ^13^C NMR (75 MHz, CDCl_3_) *δ* 172.9, 172.0, 163.2, 141.0, 137.2, 133.5, 130.5, 129.3, 129.1, 125.4 122.9, 116.2, 116.1, 96.9, 62.3, and 42.2; HR-MS *m*/*z* (pos): 324.1110 C_18_H_16_O_4_N_2_ (calcd. 325.1183).

*N-heptyl-4-hydroxy-1-methyl-2-oxo-1,2-dihydroquinoline-3-carboxamide* (**3e**); the compound was synthesized according to the general procedure (Method A) starting from quinolinone **2a** (250 mg, 1.07 mmol) and 1-heptylamine (123 mg, 1.07 mmol). After the work-up procedure, the product was obtained pure after flash column chromatography (petroleum ether:ethyl acetate/98:2), as a white powder. Yield: 70%; ^1^H NMR (300 MHz, CDCl_3_) *δ* 17.11 (s, 1H, -OH), 10.12 (s, 1H, -NH), 8.01 (dd, *J =* 8.1, 1.5 Hz, 1H, Ar-H), 7.49 (ddd, *J =* 8.4, 7.2, 1.5 Hz, 1H, Ar-H), 7.13 (m, 2H, Ar-H), 3.49 (s, 3H, N-CH_3_), 3.27 (m, 2H, H-11), 1.49 (quint, *J =* 6.9 Hz, 2H, H-12), 1.21 (m, 8H, H-13, H-14, H-15, H-16), 0.73 (t, *J =* 6.3 Hz, 3H, H-17); ^13^C NMR (75 MHz, CDCl_3_) *δ* 172.2, 171.1, 163.0, 140.0, 133.7, 125.7, 122.5, 116.6, 114.3, 99.4, 39.3, 31.9, 29.5, 29.3, 29.1, 27.2, 22.8, and 14.2; HR-MS *m*/*z* (pos): 316.1787 C_18_H_24_O_3_N_2_ (calcd. 317.1860).

*4-hydroxy-N-(4-hydroxyphenyl)-1-methyl-2-oxo-1,2-dihydroquinoline-3-carboxamide* (**3f**); the compound was synthesized according to the general procedure (Method B) starting from quinolinone **2a** (250 mg, 1.07 mmol) and 4-aminophenol (117 mg, 1.07 mmol). After the work-up procedure, the product was obtained upon recrystallization from methanol and dichloromethane as beige crystals. Yield: 49%; ^1^H NMR (300 MHz, CDCl_3_) *δ* 16.66 (s, 1H, -OH), 12.05 (s, 1H, -NH), 8.64 (s, 1H, Ar-OH), 7.95 (dd, *J =* 8.1, 1.5 Hz, 1H, Ar-H), 7.47 (ddd, *J* = 8.1, 7.5, 1.5 Hz, 1H, Ar-H), 7.19 (m, 3H, Ar-H), 7.07 (t, *J =* 7.8 Hz, 1H, Ar-H), 6.57 (d, *J =* 8.7 Hz, 2H, Ar-H), 3.47 (s, 3H, N-CH_3_); ^13^C NMR (75 MHz, CDCl_3_) *δ* 171.3, 168.1, 162.0, 154.2, 139.2, 133.4, 128.2, 124.6, 122.1, 122.0, 115.4, 115.1, 113.9, 96.3, and 28.7; HR-MS m/z (pos): 310.0954 C_17_H_14_O_4_N_2_ (calcd. 311.1026).

*4-hydroxy-N-(4-hydroxyphenyl)-2-oxo-1-phenyl-1,2-dihydroquinoline-3-carboxamide* (**3g**); the compound was synthesized according to the general procedure (Method B) starting from quinolinone **2b** (316 mg, 1.07 mmol) and 4-aminophenol (117 mg, 1.07 mmol). After the work-up procedure, the product was obtained upon recrystallization from methanol and dichloromethane as a purple solid. Yield: 33%; ^1^H NMR (300 MHz, DMSO-*d_6_/*CDCl_3_) δ 17.08 (s, 1H, -OH), 12.01 (s, 1H, -NH), 8.99 (s, 1H, Ar-OH), 8.14 (d, *J* = 8.4 Hz, 1H, Ar-H), 7.51 (m, 4H, Ar-H), 7.33 (d, *J* = 8.7 Hz, 2H, Ar-H), 7.25 (m, 3H, Ar-H), 6.71 (d, *J* = 8.7 Hz, 2H, Ar-H), 6.58 (d, *J* = 8.4 Hz, 1H, Ar-H); ^13^C NMR (75 MHz, DMSO-*d*_6_/CDCl_3_) *δ* 172.2, 168.0, 162.3, 154.3, 140.1, 136.4, 134.3, 133.1, 129.7, 128.7, 128.4, 128.0, 124.3, 122.3, 122.0, 115.6, 115.1, and 96.3; HR-MS *m*/*z* (pos): 372.1110 C_22_H_16_O_4_N_2_ (calcd. 373.1183).

*4-hydroxy-1-methyl-N-(2-methylcyclohexyl)-2-oxo-1,2-dihydroquinoline-3-carboxamide* (**3h**); the compound was synthesized according to the general procedure (Method A) starting from quinolinone **2a** (250 mg, 1.07 mmol) and 2-methylcyclohexanamine (120 mg, 1.07 mmol). After the work-up procedure, the product was obtained upon recrystallization from methanol as white crystals. Yield: 54%; ^1^H NMR (300 MHz, CDCl_3_) *δ* 17.44 (s, 1H, -OH), 10.24 (br, 1H, -NH), 8.22 (dd, *J =* 8.1, 1.2 Hz, 1H, Ar-H), 7.67 (ddd, *J* = 8.6, 7.2, 1.5 Hz, 1H, Ar-H), 7.35 (d, *J* = 8.4 Hz, 1H, Ar-H), 7.29 (m, 1H, Ar-H), 3.62 (m, 1H, NH-CH), 3.68 (s, 3H, N-CH_3_), 2.06 (m, 1H, aliphatic), 1.76 (m, 3H, aliphatic), 1.31 (m, 5H, aliphatic), 0.99 (d, *J =* 6.6 Hz, 3H, -CH_3_); ^13^C NMR (75 MHz, CDCl_3_) *δ* 172.3, 170.6, 163.0, 140.0, 133.7, 125.7, 122.5, 116.7, 114.3, 96.8, 54.3, 38.3, 34.4, 33.4, 29.2, 25.8, 25.5, and 19.5; HR-MS *m*/*z* (pos): 314.1630 C_18_H_22_O_3_N_2_ (calcd. 315.1703).

*4-hydroxy-N-(2-methylcyclohexyl)-2-oxo-1-phenyl-1,2-dihydroquinoline-3-carboxamide* (**3i**); the compound was synthesized according to the general procedure (Method A) starting from quinolinone **2b** (316 mg, 1.07 mmol) and 2-methylcyclohexanamine (120 mg, 1.07 mmol). After the work-up procedure, the product was obtained as white crystals upon recrystallization from methanol and dichloromethane. Yield: 49%; ^1^H NMR (300 MHz, CDCl_3_) *δ* 17.72 (s, 1H, -OH), 10.01 (d, *J =* 8.6 Hz, 1H, -NH), 8.24 (m, 1H, Ar-H), 7.45 (m, 4H, Ar-H), 7.44 (m, 1H, Ar-H), 7.30 (m, 2H, Ar-H), 6.62 (d, *J* = 8.7 Hz, 1H, Ar-H), 3.63 (m, 1H, NH-CH), 2.02 (m, 1H, aliphatic), 1.72 (m, 3H, aliphatic), 1.26 (m, 5H, aliphatic), 0.96 (d, *J* = 6.6Hz, 3H, -CH_3_); ^13^C NMR (75 MHz, CDCl_3_) *δ* 173.4, 170.6, 163.3, 141.0, 137.4, 133.3, 130.5, 129.3, 129.2, 125.3, 122.7, 116.1, 96.7, 54.4, 38.2, 34.4, 34.5, 33.50, 25.9, 25.6, and 19.5; HR-MS *m*/*z* (pos): 376.1787 C_23_H_22_O_4_N_3_ (calcd. 377.1860).

*4-hydroxy-N-(2-hydroxyphenyl)-1-methyl-2-oxo-1,2-dihydroquinoline-3-carboxamide* (**3j**); the compound was synthesized according to the general procedure (Method B) starting from quinolinone **2a** (250 mg, 1.07 mmol) and aminophenol (117 mg, 1.07 mmol). After the work-up procedure, the product was obtained as white solid without any further purification. Yield: 69%; ^1^H NMR (300 MHz, DMSO-*d*_6_) *δ* 16.88 (s, 1H, OH), 12.70 (s, 1H, OH phenolic), 10.19 (s, 1H, NH), 8.23 (d, *J =* 7.5 Hz, 1H, aromatic), 7.82 (dt, *J =* 7.8, 1.5 Hz, 1H, aromatic), 7.65 (d, *J =* 8.4 Hz, 1H, aromatic), 7.40 (t, *J =* 7.5 Hz, 1H, aromatic), 6.99 (m, 3H, aromatic), 6.84 (t, *J =* 7.5 Hz, 1H, aromatic), 3.67 (3H, s, N-CH_3_); ^13^C NMR (75 MHz, DMSO-*d*_6_) δ (ppm) 171.3, 168.8, 161.7, 147.5, 139.8, 134.5, 125.5, 125.0, 124.6, 122.7, 121.2, 119.2, 115.5, 115.1, 115.0, 96.8, and 29.3; HR-MS *m*/*z* (pos): 310.0954 C_17_H_14_O_4_N_2_ (calcd. 311.1024).

*4-hydroxy-1-methyl-2-oxo-N-(o-tolyl)-1,2,dihydroquinoline-3carboxamide* (**3k**); the compound was synthesized according to the general procedure (Method B) starting from quinolinone **2a** (250 mg, 1.07 mmol) and o-toluidine (115 mg, 1.07 mmol). After the work-up procedure, the product was obtained as a white solid without any further purification. Yield: 70%; ^1^H NMR (300 MHz, CDCl_3_) *δ* 16.84 (s, 1H, -OH), 12.36 (s, 1H, -NH), 8.26 (d, *J =* 7.5 Hz, 1H, Ar-H), 8.09 (d, *J =* 8.1 Hz, 1H, Ar-H), 7.71 (t, *J =* 8.1 Hz, 1H, Ar-H), 7.32 (m, 4H, Ar-H), 7.11 (t, *J =* 7.2 Hz, 1H, Ar-H), 3.74 (s, 3H, N-CH_3_), 2.45 (s, 3H, Ar-CH_3_); ^13^C NMR (75 MHz, CDCl_3_) *δ* 172.4, 169.6, 163.1, 140.1, 135.8, 134.1, 130.1, 129.7, 126.7, 125.8, 125.3, 123.0, 122.7, 116.4, 114.4, 97.5, 29.5, and 18.5; HR-MS *m*/*z* (pos): 308.1161 C_18_H_16_O_3_N_2_ (calcd. 309.1234).

*4-hydroxy-1-methyl-2-oxo-N-(p-tolyl)-1,2-dihydroquinoline-3-carboxamide* (**3l**); the compound was synthesized according to the general procedure (Method B) starting from quinolinone **2a** (250 mg, 1.07 mmol) and p-toluidine (115 mg, 1.07 mmol). After the work-up procedure, the product was obtained upon recrystallization from methanol and dichloromethane as a white solid. Yield: 37%; ^1^H NMR (300 MHz, CDCl_3_) *δ* 16.83 (s, 1H, -OH), 12.42 (s, 1H, -NH), 8.24 (dd, *J =* 7.5, 1.5 Hz, 1H, Ar-H), 7.70 (ddd, *J =* 8.2, 7.5, 1.5 Hz, 1H, Ar-H), 7.57 (d, *J =* 8.4 Hz, 2H, H-12, H-16), 7.38 (d, *J* = 8.7 Hz, 1H, Ar-H), 7.32 (t, *J* = 8.1 Hz, 1H, Ar-H), 7.18 (d, *J =* 8.4 Hz, 2H, H-13, H-15), 3.72 (s, 3H, N-CH_3_), 2.35 (s, 3H, Ar-CH_3_); ^13^C NMR (75 MHz, CDCl_3_) *δ* 172.2, 169.2, 162.8, 139.8, 134.6, 134.5, 133.9, 129.5, 125.6, 122.6, 121.2, 116.3, 114.3, 97.2, 29.3, and 20.9; HR-MS *m*/*z* (pos): 308.1161 C_18_H_16_O_3_N_2_ (calcd. 309.1234).

*N-(4-fluorophenyl)-4-hydroxy-1-methyl-2-oxo-1,2-dihydroquinoline-3-carboxamide* (**3m**); the compound was synthesized according to the general procedure (Method B) starting from quinolinone **2a** (250 mg, 1.07 mmol) and 4-fluoroaniline (119 mg, 1.07 mmol). After the work-up procedure, the product was obtained as a white solid without any further purification. Yield: 66%; ^1^H NMR (300 MHz, CDCl_3_/DMSO-*d*_6_) *δ* 16.18 (s, 1H, -OH), 12.22 (S, 1H, -NH), 7.85 (dd, *J* = 8.1, 1.5 Hz, 1H, Ar-H), 7.39 (ddd, *J* = 8.4, 7.2, 1.5 Hz, 1H, Ar-H), 7.31 (m, 2H, Ar-H), 7.09 (d, *J* = 8.4 Hz, 1H, Ar-H), 6.99 (t, *J* = 7.8 Hz, 1H, Ar-H), 6.74 (m, 2H, Ar-H), 3.38 (s, 3H, N-CH_3_); ^13^C NMR (75 MHz, CDCl_3_/DMSO-*d*_6_) *δ* 171.3, 168.5, 161.9, 160.4, 139.1, 133.6, 132.7, 124.6, 122.1, 122.0, 115.1, 114.8, 113.9, 96.2, and 28.7; HR-MS *m*/*z* (pos): 312.0910 C_17_H_13_O_3_FN_2_ (calcd. 313.0983).

*4-hydroxy-N-(4-hydroxyphenethyl)-2-oxo-1-phenyl-1,2-dihydroquinoline-3-carboxamide* (**3n**); the compound was synthesized according to the general procedure (Method B) starting from quinolinone **2b** (316 mg, 1.07 mmol) and 4-(2-aminoethyl)phenol (147 mg, 1.07 mmol). After the work-up procedure, the product was obtained as a white solid without any further purification. Yield: 62%; ^1^H NMR (300 MHz, CDCl_3_) *δ* 17.36 (s, 1H, -OH), 10.19 (s, 1H, -NH), 8.24 (dd, *J =* 8.1, 1.2 Hz, 1H, Ar-H), 7.56 (m, 3H, Ar-H), 7.45 (ddd, *J* = 8.4, 7.2, 1.5 Hz, 1H, Ar-H), 7.28 (m, 3H, Ar-H), 7.05 (d, *J =* 8.4 Hz, 2H, H-15, H-17), 6.69 (d, *J =* 8.4 Hz, 2H, H-14, H-18), 6.64 (d, *J =* 8.4 Hz, 1H, Ar-H), 3.61 (m, 2H, NH-CH_2_), 2.82 (t, *J =* 7.8 Hz, 2H, CH_2_-Ar); ^13^C NMR (75 MHz, CDCl_3_) δ 173.0, 171.0, 163.1, 154.3, 140.8, 137.1, 133.3, 130.6, 130.3, 129.7, 129.2, 129.0, 125.2, 122.7, 116.2, 116.1, 115.4, 96.7, 40.9, and 34.8; HR-MS *m*/*z* (pos): 400.1423 C_24_H_20_O_4_N_2_ (calcd. 401.1496).

*4-hydroxy-N-(4-hydroxyphenethyl)-1-methyl-2-oxo-1,2-dihydroquinoline-3-carboxamide* (**3o**); the compound was synthesized according to the general procedure (Method B) starting from quinolinone **2a** (250 mg, 1.07 mmol) and 4-(2-aminoethyl)phenol (147 mg, 1.07 mmol). After the work-up procedure, the product was obtained as a white solid without any further purification. Yield: 65%; ^1^H NMR (300 MHz, CDCl_3_) *δ* 16.92 (s, 1H, -OH), 10.09 (s, 1H, -NH), 7.88 (dd, *J =* 8.2, 1.5 Hz, 1H, Ar-H), 7.42 (t, *J* = 7.8 Hz, 1H, Ar-H), 7.11 (d, *J* = 9.0 Hz, 1H, Ar-H), 7.02 (t, *J* = 7.8 Hz, 1H, Ar-H), 6.77 (d, *J =* 8.1 Hz, 2H, H-15, H-17), 6.48 (d, *J =* 8.7 Hz, 2H, H-14, H-18), 3.38 (s, 3H, N-CH_3_), 3.32 (m, 2H, NH-CH_2_), 2.55 (t, *J =* 7.2 Hz, 2H, CH_2_-Ar), 2.30 (br, 1H, Ar-OH); ^13^C NMR (75 MHz, CDCl_3_) *δ* 171.1, 170.3, 161.7, 155.2, 153.2, 139.2, 133.1, 128.9, 128.5, 124.5, 121.6, 118.8, 114.9, 113.7, 98.7, 30.0, and 28.4; HR-MS *m*/*z* (pos): 338.1267 C_19_H_18_O_4_N_2_ (calcd. 339.1339).

*4-hydroxy-N-(2-hydroxy-1-phenylethyl)-1-methyl-2-oxo-1,2-dihydroquinoline-3-carboxamide* (**3p**); the compound was synthesized according to the general procedure (Method B) starting from quinolinone **2a** (250 mg, 1.07 mmol) and 2-amino-2-phenylethan-1-ol (147 mg, 1.07 mmol). After the work-up procedure, the product was obtained as a white solid without any further purification. Yield: 70%; ^1^H NMR (300 MHz, CDCl_3_) *δ* (ppm) 10.72 (s, 1H, -NH), 8.23 (d, *J* = 7.8 Hz, 1H, Ar-H), 7.70 (ddd, *J* = 8.4, 7.2, 0.9Hz, 1H, Ar-H), 7.37 (m, 7H, Ar-H), 7.98 (dd, *J* = 7.8, 2.4 Hz, 1H, NH-CH), 3.82 (br, 1H, H-12a), 3.69 (s, 3H, N-CH_3_), 3.62 (br, 1H, H-12b), 3.06 (brs, 1H, Ar-OH); ^13^C NMR (75 MHz, CDCl_3_) *δ* (ppm) 172.1, 172.0, 162.8, 141.9, 140.2, 134.0, 128.7, 128.2, 126.0, 125.8, 122.6, 114.4, 74.0, 47.4, and 29.4; HR-MS *m*/*z* (pos): 338.1267 C_19_H_18_O_4_N_2_ (calcd. 339.1339).

*4-hydroxy-N-(2-hydroxy-1-phenylethyl)-2-oxo-1-phenyl-1,2-dihydroquinoline-3-carboxamide* (**3q**); the compound was synthesized according to the general procedure (Method B) starting from quinolinone **2b** (316 mg, 1.07 mmol) and 2-amino-2-phenylethan-1-ol (147 mg, 1.07 mmol). After the work-up procedure, the product was obtained as a white solid without any further purification. Yield: 72%; ^1^H NMR (300 MHz, CDCl_3_) *δ* (ppm) 10.51 (br, 1H, -NH), 8.23 (br, 1H, Ar-H), 7.57 (m, 3H, Ar-H), 7.35 (m, 9H, Ar-H), 6.64 (br, 1H, Ar-H), 4.91 (br, 1H, NH-CH), 3.71 (br, 1H, H-12a), 3.58 (br, 1H, H-12b), 3.00 (brs, 1H, CH_2_-OH); ^13^C NMR (75 MHz, CDCl_3_) *δ* (ppm) 172.7, 171.9, 162.9, 141.7, 140.9, 137.1, 133.4, 130.3, 130.2, 129.1, 129.0, 128.5, 127.9, 125.8, 125.2, 122.7, 116.1, 73.8, and 47.5; HR-MS *m*/*z* (pos): 400.1423 C_24_H_20_O_4_N_2_ (calcd. 401.1496).

*4-hydroxy-2-oxo-1-phenyl-N-(p-tolyl)-1,2-dihydroquinoline-3-carboxamide* (**3r**); the compound was synthesized according to the general procedure (Method B) starting from quinolinone **2b** (316 mg, 1.07 mmol) and p-toluedine (115 mg, 1.07 mmol). After the work-up procedure, the product was obtained as a white solid without any further purification. Yield: 62%; ^1^H NMR (300 MHz, CDCl_3_) δ (ppm) 12.18 (s, 1H, -NH), 8.28 (d, *J* = 8.1 Hz, 1H, Ar-H), 7.57 (m, 6H, Ar-H), 7.30 (m, 3H, Ar-H), 7.14 (d, *J* = 8.1 Hz, 2H, Ar-H), 6.66 (d, *J* = 8.4 Hz, 1H, Ar-H), 2.33 (s, 3H, N-CH_3_); ^13^C NMR (75 MHz, CDCl_3_) δ (ppm) 173.3, 169.3, 163.3, 140.9, 137.2, 134.7, 134.6, 133.6, 130.6, 129.6, 129.5, 129.1, 125.4, 123.0, 121.4, 116.3, and 21.1; HR-MS *m*/*z* (pos): 370.1317 C_23_H_18_O_3_N_2_ (calcd. 371.1390).

*N-(4-fluorophenyl)-4-hydroxy-2-oxo-1-phenyl-1,2-dihydroquinoline-3-carboxamide* (**3s**); the compound was synthesized according to the general procedure (Method B) starting from quinolinone **2b** (316 mg, 1.07 mmol) and 4-fluoroaniline (119 mg, 1.07 mmol). After the work-up procedure, the product was obtained as a white solid without any further purification. Yield: 62%; ^1^H NMR (300 MHz, CDCl_3_) *δ* 16.85 (s, 1H, -OH), 12.27 (s, 1H, -NH), 8.27 (d, *J* = 8.1 Hz, 1H, Ar-H), 7.64 (m, 5H, Ar-H), 7.49 (t, *J* = 7.5 Hz, 1H, Ar-H), 7.30 (m, 3H, Ar-H), 7.03 (m, 2H, Ar-H), 6.67 (d, *J* = 8.4 Hz, 1H, Ar-H); ^13^C NMR (75 MHz, CDCl_3_) *δ* 173.1, 169.1, 163.1, 161.3, 158.1, 140.8, 137.0, 133.6, 130.4, 129.4, 128.9, 125.2, 122.9, 122.7, 116.2, 115.7, 115.5, 97.0, and 67.1; HR-MS *m*/*z* (pos): 374.1067 C_22_H_15_O_3_FN_2_ (calcd. 375.1139).

*4-hydroxy-1-methyl-2-oxo-N-(pyridin-4-yl)-1,2-dihydroquinoline-3-carboxamide* (**3t**); the compound was synthesized according to the general procedure (Method B) starting from quinolinone **2a** (250 mg, 1.07 mmol) and 2-aminopyridine (101 mg, 1.07 mmol). After the work-up procedure, the product was obtained as a white solid without any further purification. Yield: 67%; ^1^H NMR (300 MHz, CDCl_3_) *δ* (ppm) 15.98 (s, 1H, -OH), 12.96 (s, 1H, -NH), 8.55 (d, *J* = 5.4 Hz, 2H, H-13, H-15), 8.25 (d, *J* = 8.1 Hz, 1H, Ar-H), 7.75 (br, 1H, Ar-H), 7.68 (d, *J* = 5.4 Hz, 2H, H-12, H-16), 7.37 (m, 2H, Ar-H), 3.73 (s, 3H, N-CH_3_); HR-MS *m*/*z* (pos): 295.0957 C_16_H_13_O_3_N_3_ (calcd. 296.1030).

*4-hydroxy-2-oxo-1-phenyl-N-(pyridin-4-yl)-1,2-dihydroquinoline-3-carboxamide* (**3u**); the compound was synthesized according to the general procedure (Method B) starting from quinolinone **2b** (316 mg, 1.07 mmol) and 2-aminopyridine (101 mg, 1.07 mmol). After the work-up procedure, the product was obtained as a white solid without any further purification. Yield: 62%; ^1^H NMR (600 MHz, CDCl_3_) *δ* 12.98 (s, 1H, -NH), 8.53 (m, 2H, H-13, H-15), 8.29 (d, *J* = 7.8 Hz, 1H, Ar-H), 7.77 (m, 2H, H-12, H-16), 7.67 (m, 2H, Ar-H), 7.62 (d, *J* = 7.8 Hz, 1H, Ar-H), 7.54 (t, *J* = 7.8 Hz, 1H, Ar-H), 7.35 (t, *J* = 7.8 Hz, 1H, Ar-H), 7.31 (m, 2H, Ar-H), 6.69 (d, *J* = 9.0 Hz, 1H, Ar-H); ^13^C NMR (150 MHz, CDCl_3_) *δ* 173.7, 170.8, 163.2, 148.0, 141.2, 136.7, 134.6, 130.7, 129.8, 128.9, 125.6, 123.5, 116.5, 115.7, 115.4, and 97.2; HR-MS *m*/*z* (pos): 357.1113 C_21_H_15_O_3_N_3_ (calcd. 358.1186).

#### 3.1.2. Synthesis of 1-Ethyl-2,4-dihydro-1H-3,1-benzoxazine-2,4-dione (**5**)

To a stirred solution of isatoic anhydride (**4**) (2 g, 12.3 mmol) and sodium hydride (NaH, 60% *w*/*w* in oil, 444 mg, 18.5 mmol) in dimethylformamide (DMF, 50 mL), iodoethane (1.2 mL, 14.8 mmol) is added under cooling and the reaction mixture is refluxed and stirred at room temperature for 24 h, under inert atmosphere. The reaction is monitored by TLC. At the end of the reaction, the mixture is poured into a conical flask containing water and ice and then extracted three times with Et_2_O. The organic phase is collected, dried over anhydrous Na_2_SO_4,_ and concentrated under reduced pressure. The final product (**5**) is obtained as a beige solid after additional rinsing with Et_2_O and used in the next step without further purification. Yield: 36%; ^1^H NMR (300 MHz, CDCl_3_) *δ* 8.11 (dd, *J* = 9.0 Hz, *J* = 3.0 Hz, 1H, Ar-H), 7.71 (td, *J* = 9.0 Hz, *J* = 3.0 Hz, 1H, Ar-H), 7.22 (d, *J* = 9.0 Hz, 1H, Ar-H), 7.14 (d, *J* = 9.0 Hz, 1H, Ar-H), 4.09 (q, *J* = 6.0 Hz, 2H, N-CH_2_), 1.32 (t, *J* = 9.0 Hz, 3H, N-CH_2_-CH_3_).

#### 3.1.3. Synthesis of Methyl 1-Ethyl-4-hydroxy-2-oxo-1,2-dihydroquinoline-3-carboxylate (**6**)

To a stirred solution of NaH (60% dispersion in mineral oil) (235 mg, 5.9 mmol) and 25 mL DMF, 1 eq. of N-substituted isatoic anhydride (**5**) (560 mg, 2.9 mmol) was added, followed by the addition of 5 eq. of dimethyl malonate (1.70 mL, 14.8 mmol) at 0 °C. The reaction mixture was refluxed at 80 °C under an inert atmosphere for 3 h, and the reaction was monitored by TLC. After completion of the reaction, the mixture was cooled in an ice-water bath, acidified with HCl (10% aqueous solution), and then extracted with Et_2_O (3 × 25 mL); the combined organic layers were collected, dried over NaSO_4_, and evaporated under reduced pressure. The desired quinolinone product **6** was obtained as a beige powder, and it was further purified by recrystallization from methanol/dichloromethane. Yield: 220 mg (35%); m.p. 138 °C; ^1^HNMR (600 MHz, CDCl_3_,) *δ* (ppm) 14.01 (s, 1H, -OH), 8.20 (dd, *J* = 7.8, 0.6 Hz, 1H, Ar–H), 7.68 (td, *J* = 7.2, 1.8 Hz, 1H, Ar–H), 7.33 (d, *J* = 8.4 Hz, 1H, Ar–H), 7.25 (t, *J* = 7.8 Hz, 1H, Ar–H), 4.31 (q, *J* = 7.2 Hz, 2H, N–CH_2_–CH_3_), 4.04 (s, 2H, COOCH_3_), 1.33 (t, *J* = 6.6 Hz, 3H, N–CH_2_–CH_3_).

#### 3.1.4. Synthesis of the N-(2-Aminoethyl)-1-ethyl-4-hydroxy-2-oxo-1,2-dihydroquinoline-3-carboxamide (**7**) 

A sample of 1 eq. of **6** (150 mg, 0.61 mmol) and 2 eq. of ethylenediamine (81.1 μL, 1.21 mmol) were added to 10 mL of toluene. The reaction mixture was refluxed under an inert atmosphere for 2.5 h, and the reaction was monitored by TLC. After completion of the reaction, the mixture was cooled in an ice-water bath, and the precipitate was formed, filtered, and washed with Et_2_O. The desirable amide product (**7**) was obtained as a white powder further purified by recrystallization from methanol/dichloromethane. Yield: 93 mg (55%); m.p. 161 °C; ^1^HNMR (300 MHz, DMSO-*d*_6_) *δ* (ppm) 10.44 (s, 1H, NH), 8.09 (d, *J* = 7.2 Hz, 1H, Ar-H), 7.70 (t, *J* = 7.5 Hz, 1H, Ar-H), 7.55 (d, *J* = 8.4 Hz, 1H, Ar-H), 7.27 (t, *J* = 7.2 Hz, 1H, Ar-H), 4.25 (q, *J* = 4.5 Hz, 2H, N-CH_2_-CH_3_), 3.36 (br, 2H, CONH-CH_2_- CH_2_-NH_2_), 2.77 (br, 2H, CONH-CH_2_-CH_2_-NH_2_), 1.19 (t, *J* = 6.3 Hz, 3H, N-CH_2_-CH_3_); ^13^C NMR (75 MHz, DMSO-*d*_6_) *δ* 171.9, 170.5, 161.7, 138.7, 133.2, 125.1, 121.5, 117.7, 114.5, 96.9, 40.4, 40.3, 36.2, and 12.9.

#### 3.1.5. General Procedure for the Synthesis of Acetyl Phenolic Acid Derivatives (**9a**, **9b**, **9e**, and **9f**)

A sample of 1 eq. of the appropriate phenolic acid and 2 eq. of acetic anhydride were added to the pyridine. The mixture was then stirred overnight at 80 °C, under an inert atmosphere and monitored by TLC. After completion of the reaction, pyridine was evaporated under reduced pressure, resulting in a solid residue, which was then washed with diethyl ether solvent and filtered. The desirable acetyl derivatives (**9a**, **9b**, **9e**, and **9f**) were obtained and used without further purification.

*4-acetoxybenzoic acid* (**9a**): the compound was synthesized according to the general procedure, starting from 4-hydroxybenzoic acid (**8a**) (400 mg, 2.22 mmol) and acetic anhydride (419.0 μL, 4.44 mmol) in 6 mL pyridine. After the work-up procedure, the product was obtained as a white solid. Yield: 95%.

*4-acetoxy-3,5-dimethoxybenzoic acid* (**9b**): the compound was synthesized according to the general procedure, starting from 4-hydroxy-3,5-dimethoxybenzoic acid (**8b**) (400 mg, 1.67 mmol) and acetic anhydride (314.2 μL, 3.33 mmol) in 5 mL pyridine. After the work-up procedure, the product was obtained as a white solid. Yield: 70%.

*(E)-3-(4-acetoxyphenyl)acrylic acid* (**9e**): the compound was synthesized according to the general procedure, starting from (E)-3-(4-hydroxyphenyl)acrylic acid (**8e**) (400 mg, 1.94 mmol) and acetic anhydride (366.1 μL, 3.88 mmol) in 5 mL pyridine. After the work-up procedure, the product was obtained as a white solid. Yield: 76%; ^1^HNMR (600 MHz, DMSO-*d*_6_) δ (ppm) 12.40 (s, 1H, CO**OH**), 7.74 (d, *J* = 8.4 Hz, 2H, Ar-H), 7.59 (d, *J* = 16.2 Hz, 1H, H-a), 7.18 (d, *J* = 8.4 Hz, 2H, Ar-H), 6.51 (d, *J* = 15.9 Hz, H-b), 2.28 (s, 3H, Ar-OCOCH3).

*(E)-3-(4-acetoxy-3-methoxyphenyl)acrylic acid* (**9f**): the compound was synthesized according to the general procedure, starting from (E)-3-(4-hydroxy-3-methoxyphenyl)acrylic acid (**8f**) (400 mg, 1.69 mmol) and acetic anhydride (319.5 μL, 3.38 mmol) in 5 mL pyridine. After the work-up procedure, the product was obtained as a white solid. Yield: 95%.

#### 3.1.6. General Procedure for the Synthesis of the Acetyl Chlorides **10a–10g**

A sample of 1 eq. of the appropriate carboxylic acid and 4 eq. of thionyl chloride (SOCl_2_) were added to toluene. The mixture was stirred at 90 °C for 45 min under an inert atmosphere. After completion of the reaction, toluene was evaporated under reduced pressure, and the residue was dried in a high vacuum pump. The desirable acyl chlorides **10a**–**10g** were obtained and used without purification directly in the next reaction, assuming a 100% yield.

*4-(chlorocarbonyl)phenyl acetate* (**10a**): the compound was synthesized according to the general procedure, starting from 4-acetoxybenzoic acid (**9a**) (264 mg, 1.47 mmol) and SOCl_2_ (425.2 μL, 5.86 mmol) in 4.0 mL toluene. 

*4-(chlorocarbonyl)-2,6-dimethoxyphenyl acetate* (**10b**): the compound was synthesized according to the general procedure, starting from 4-acetoxy-3,5-dimethoxybenzoic acid (**9a**) (280 mg, 1.17 mmol) and SOCl_2_ (338.2 μL, 4.66 mmol) in 3.5 mL toluene.

*Benzoyl chloride* (**10c**): the compound was synthesized according to the general procedure, starting from benzoic acid (**8c**) (300 mg, 2.46 mmol) and SOCl_2_ (712.8 μL, 9.83 mmol) in 5.5 mL toluene.

*2-hydroxybenzoyl chloride* (**10d**): the compound was synthesized according to the general procedure, starting from 2-hydroxybenzoic acid (**8d**) (300 mg, 2.17 mmol) and SOCl_2_ (630.3 μL, 8.69 mmol) in 6.0 mL toluene.

*(E)-4-(3-chloro-3-oxoprop-1-en-1-yl)phenyl acetate* (**10e**): the compound was synthesized according to the general procedure, starting from (E)-3-(4-acetoxyphenyl)acrylic acid (**9e**) (300 mg, 1.45 mmol) and SOCl_2_ (422.2 μL, 5.82 mmol) in 3.5 mL toluene.

*(E)-4-(3-chloro-3-oxoprop-1-en-1-yl)-2-methoxyphenyl acetate* (**10f**): the compound was synthesized according to the general procedure, starting from (E)-3-(4-acetoxy-3-methoxyphenyl)acrylic acid (**9f**) (300 mg, 1.27 mmol) and SOCl_2_ (368.5 μL, 5.08 mmol) in 3.0 mL toluene.

*(E)-3-(4-methoxyphenyl)acryloyl chloride* (**10g**): the compound was synthesized according to the general procedure, starting from (E)-3-(4-methoxyphenyl)acrylic acid (**8g**) (200 mg, 1.12 mmol) and SOCl_2_ (325.7 μL, 4.49 mmol) in 3.0 mL toluene.

#### 3.1.7. General Procedure for the Synthesis of Hybrid Compounds **11a**–**11g**

The appropriate chloride (1.5 eq.) **10a–10c**, **10e–10h**, and carboxamide **7** (1 eq.) were diluted in THF, and Et_3_N was added. The mixture was then stirred overnight at 50 °C under an inert atmosphere and monitored by TLC. After completion of the reaction, the mixture was cooled to room temperature and then extracted three times with ethyl acetate; the organic layer was collected, dried over Na_2_SO_4_, and evaporated under reduced pressure. The desirable final hybrid compounds (**11a–11g**) were obtained in a solid form after the suitable purification process. 

*4-((2-(1-ethyl-4-hydroxy-2-oxo-1,2-dihydroquinoline-3-carboxamido)ethyl)carbamoyl)phenyl acetate* (**11a**): the compound was synthesized according to the general procedure, starting from 4-(chlorocarbonyl)-phenyl acetate (**10a**) (290.6 mg, 1.46 mmol) and carboxamide **7** (268.5 mg, 0.97 mmol), in 5 mL of THF and 0.44 mL of Et_3_N. After the work-up procedure, the product was obtained upon recrystallization from ethyl acetate, as a white solid. Yield: 95%; m.p. 137–145 °C; ^1^HNMR (600 MHz, DMSO-*d*_6_) *δ* (ppm) 17.35 (s, 1H, OH), 10.48 (s, 1H, CONHCH_2_), 8.68 (s, 1H, CH_2_NHCO), 8.11 (d, *J* = 7.8 Hz, 1H, Ar-H), 7.88 (d, *J* = 8.4 Hz, 2H, H-3′ & H-5′), 7.80 (t, *J* = 7.8 Hz, 1H, Ar-H), 7.66 (d, *J* = 8.4 Hz, 1H, Ar-H), 7.37 (t, *J* = 7.8 Hz, 1H, Ar-H), 7.22 (d, *J* = 8.4 Hz, 2H, H-2′ & H-6′), 4.29 (q, *J* = 7.2 Hz, 2H, NCH_2_CH_3_), 3.59 (br, 2H, H-11), 3.49 (br, 2H, H-12), 2.29 (s, 3H, CH_3_COOAr), 1.21 (t, *J* = 7.2 Hz, 3H, NCH_2_CH_3_);^13^C NMR (150 MHz, DMSO-*d*_6_) *δ* ppm 171.06, 168.95, 165.94, 161.14, 152.60, 138.65, 134.31, 132.06, 128.66, 124.75, 122.32, 121.68, 115.33, 115.00, 95.97, 38.89, 38.19, 36.64, 20.85, and 12.72; HR-MS *m*/*z* (neg): 437.1587 C_23_H_22_O_6_N_3_ (calcd. 436.15055).

*4-((2-(1-ethyl-4-hydroxy-2-oxo-1,2-dihydroquinoline-3-carboxamido)ethyl)carbamoyl)-2,6-dimethoxyphenyl acetate* (**11b**): the compound was synthesized according to the general procedure, starting from 4-(chlorocarbonyl)-2,6-dimethoxyphenyl acetate (**10b**) (305.3 mg, 1.18 mmol) and carboxamide **7** (216.6 mg, 2.79 mmol), in 4 mL of THF and 0.36 mL of Et_3_N. After the work-up procedure, the product was obtained as a white solid upon flash column chromatography. Yield: 80%; m.p. 192–194 °C; ^1^H NMR (600 MHz, DMSO-*d*_6_) *δ* (ppm) 17.36 (s, 1H, OH), 10.49 (s, 1H, CONHCH_2_), 8.70 (s, 1H, CH_2_NHCO), 8.11 (d, *J* = 7.8 Hz, 1H, Ar-H), 7.81 (t, *J* = 8.4 Hz, 1H, Ar-H), 7.67 (d, *J* = 8.4 Hz, 1H, Ar-H), 7.37 (t, *J* = 7.2 Hz, 1H, Ar-H), 7.21 (s, 2H, H-3′ & H-5′), 4.29 (q, *J* = 7.2 Hz, 2H, NCH_2_CH_3_), 3.80 (s, 6H, 2 x Ar-OCH_3_), 3.61 (br, 2H, H-11), 3.50 (br, 2H, H-12), 2.26 (s, 3H, CH_3_COOAr), 1.21 (t, *J* = 7.2 Hz, 3H, NCH_2_CH_3_); ^13^C NMR (150 MHz, DMSO-*d_6_*) *δ* ppm 171.08, 171.02, 167.85, 166.02, 161.17, 151.49, 138.67, 134.36, 132.74, 130.12, 124.75, 122.37, 115.32, 115.04, 104.18, 95.98, 56.06, 38.95, 38.14, 36.64, 20.14, and 12.71; HR-MS *m*/*z* (neg): 497.1798 C_25_H_26_O_8_N_3_ (calcd. 496.17166).

*N-(2-benzamidoethyl)-1-ethyl-4-hydroxy-2-oxo-1,2-dihydroquinoline-3-carboxamide* (**11c**): the compound was synthesized according to the general procedure, starting from benzoyl chloride (**10c**) (275.0 mg, 1.95 mmol) and carboxamide **7** (359.1 mg, 1.30 mmol), in 6.5 mL of THF and 0.59 mL of Et_3_N. After the work-up procedure, the product was obtained as a white solid upon flash column chromatography. Yield: 56%; m.p. 145–150 °C; ^1^H NMR (300 MHz, DMSO-*d_6_*) *δ* (ppm) 17.28 (s, 1H, OH), 10.44 (br, 1H, CONHCH_2_), 8.63 (br, 1H, CH_2_NHCO), 8.08 (dd, *J* = 8.1, 1.2 Hz, 1H, Ar-H), 7.82 (m, 2H, Ar-H), 7.77 (d, *J* = 7.2 Hz, 1H, Ar-H), 7.64 (d, *J* = 8.7 Hz, 1H, Ar-H), 7.48 (m, 3H, Ar-H), 7.35 (t, *J* = 7.5 Hz, 1H, Ar-H), 4.28 (q, *J* = 7.2 Hz, 2H, NCH_2_CH_3_), 3.61 (br, 2H, H-11), 3.50 (br, 2H, H-12), 1.21 (t, *J* = 6.9 Hz, 3H, NCH_2_CH_3_); ^13^C NMR (75 MHz, DMSO-*d_6_*) *δ* ppm 171.07, 171.01, 166.68, 161.14, 138.65, 134.52, 134.34, 131.16, 128.26, 127.21, 124.76, 122.35, 115.33, 115.02, 95.97, 38.878, 38.212, 36.64, and 12.74; HR-MS *m*/*z* (neg): 379.1532 C_21_H_20_O_4_N_3_ (calcd. 378.14505).

*(E)-4-(3-((2-(1-ethyl-4-hydroxy-2-oxo-1,2-dihydroquinoline-3-carboxamido)ethyl)amino)-3-oxoprop-1-en-1-yl)phenyl acetate* (**11d**): the compound was synthesized according to the general procedure, starting from (E)-4-(3-chloro-3-oxoprop-1-en-1-yl)-phenyl acetate (**10e**) (334.1 mg, 1.48 mmol) and carboxamide **7** (272.9 mg, 0.99 mmol), in 5 mL of THF and 0.45 mL of Et_3_N. After the work-up procedure, the product was obtained upon recrystallization from hexane as a white solid. Yield: 57%; m.p. 147–150 °C; ^1^H NMR (600 MHz, DMSO-*d*_6_) *δ* (ppm) 17.33 (s, 1H, OH), 10.43 (br, 1H, CONHCH_2_), 8.34 (br, 1H, CH_2_NHCO), 8.10 (d, *J* = 7.8 Hz, 1H, Ar-H), 7.80 (t, *J* = 7.2 Hz, 1H, Ar-H), 7.66 (d, *J* = 8.4 Hz, 1H, Ar-H), 7.61 (d, *J* = 8.4 Hz, 2H, H-2′ & H-6′), 7.44 (d, *J* = 15.6 Hz, 1H, H-16), 7.36 (t, *J* = 7.2 Hz, 1H, Ar-H), 7.17 (d, *J* = 8.4 Hz, 1H, Ar-H), 6.60 (d, *J* = 15.6 Hz, 1H, H-15), 4.29 (q, *J* = 7.2 Hz, 2H, NCH_2_CH_3_), 3.54 (br, 2H, H-11), 3.42 (br, 2H, H-12), 2.27 (s, 3H, CH_3_COOAr), 1.21 (t, *J* = 7.2 Hz, 3H, NCH_2_CH_3_); ^13^C NMR (150 MHz, DMSO-*d*_6_) *δ* ppm 171.07, 170.97, 169.04, 165.26, 161.14, 151.14, 138.66, 137.86, 134.33, 132.51, 128.66, 124.76, 122.35, 122.07, 115.32, 115.01, 95.94, 38.38, 36.64, 20.85, and 12.73; HR-MS *m*/*z* (neg): 463.1743 C_25_H_24_O_6_N_3_ (calcd. 462.16611).

(E)-4-(3-((2-(1-ethyl-4-hydroxy-2-oxo-1,2-dihydroquinoline-3-carboxamido)ethyl)amino)-3-oxoprop-1-en-1-yl)-2-methoxyphenyl acetate (**11e**): the compound was synthesized according to the general procedure, starting from (E)-4-(3-chloro-3-oxoprop-1-en-1-yl)-2-methoxyphenyl acetate (**10f**) (320.9 mg, 1.26 mmol) and carboxamide **7** (231.3 mg, 0.84 mmol), in 4.5 mL of THF and 0.38 mL of Et_3_N. After the work-up procedure, the product was obtained upon recrystallization from ethyl acetate as a white solid. Yield: 59%; m.p. 115–121 °C; ^1^H NMR (600 MHz, DMSO-*d*_6_) *δ* (ppm) 17.33 (s, 1H, OH), 10.43 (br, 1H, CONHCH_2_), 8.32 (br, 1H, CH_2_NHCO), 8.11 (d, *J* = 7.8 Hz, 1H, Ar-H), 7.80 (t, *J* = 7.8 Hz, 1H, Ar-H), 7.66 (d, *J* = 8.7 Hz, 1H, Ar-H), 7.43 (d, *J* = 15.6 Hz, 1H, H-16), 7.36 (t, *J* = 7.2 Hz, 1H, Ar-H), 7.32 (d, *J* = 1.2 Hz, 1H, H-3′), 7.16 (dd, *J* = 8.4, 1.8 Hz, 1H, H-5′), 7.11 (d, *J* = 8.4 Hz, 1H, H-6′), 6.23 (d, *J* = 15.6 Hz, 1H, H-15), 4.29 (q, *J* = 7.2 Hz, 2H, NCH_2_CH_3_), 3.81 (s, 3H, OCH_3_), 3.54 (br, 2H, H-11), 3.42 (br, 2H, H-12), 2.26 (s, 3H, CH_3_COOAr), 1.21 (t, *J* = 7.2 Hz, 3H, NCH_2_CH_3_); ^13^C NMR (150 MHz, DMSO-d_6_) *δ* ppm 171.14, 171.04, 168.54, 165.34, 161.19, 151.09, 140.19, 138.71, 138.32, 134.44, 133.90, 124.84, 123.32, 122.45, 122.29, 120.20, 115.13, 111.53, 95.99, 55.81, 38.45, 36.71, 20.48, and 12.83; HR-MS *m*/*z* (neg): 493.1849 C_25_H_26_O_7_N_3_ (calcd. 492.17709).

*(E)-1-ethyl-4-hydroxy-N-(2-(3-(4-methoxyphenyl)acrylamido)ethyl)-2-oxo-1,2-dihydroquinoline-3-carboxamide* (**11f**): the compound was synthesized according to the general procedure, starting from (E)-3-(4-methoxyphenyl)acryloyl chloride (**10g**) (136.2 mg, 0.69 mmol) and carboxamide **7** (126.6 mg, 0.46 mmol), in 2.5 mL of THF and 0.21 mL of Et_3_N. After the work-up procedure, the product was obtained upon recrystallization from methanol as a white solid. Yield: 55%; m.p. 153–155 °C; ^1^H NMR (600 MHz, DMSO-*d*_6_) *δ* (ppm) 17.30 (s, 1H, OH), 10.42 (br, 1H, CONHCH_2_), 8.24 (br, 1H, CH_2_NHCO), 8.10 (d, *J* = 7.8 Hz, 1H, Ar-H), 7.79 (t, *J* = 7.8 Hz, 1H, Ar-H), 7.65 (d, *J* = 8.7 Hz, 1H, Ar-H), 7.50 (d, *J* = 8.4 Hz, 2H, H-2′ & H-6′), 7.35 (m, 2H, H-16 & Ar-H), 6.96 (d, *J* = 8.4 Hz, 2H, H-3′ & H-5′), 6.48 (d, *J* = 15.6 Hz, 1H, H-15), 4.28 (q, *J* = 7.2 Hz, 2H, NCH_2_CH_3_), 3.78 (s, 3H, ArOCH_3_), 3.53 (br, 2H, H-11), 3.41 (br, 2H, H-12), 1.21 (t, *J* = 7.2 Hz, 3H, NCH_2_CH_3_); ^13^C NMR (150 MHz, DMSO-*d*_6_) *δ* ppm 171.06, 170.95, 165.62, 161.13, 160.29, 138.64, 138.49, 134.30, 129.08, 127.39, 124.74, 122.32, 119.49, 115.32, 114.99, 114.33, 95.93, 55.22, 38.43, 38.33, 36.63, and 12.73; HR-MS *m*/*z* (neg): 435.1794 C_24_H_24_O_5_N_3_ (calcd. 434.17123).

*(E)-N-(2-cinnamamidoethyl)-1-ethyl-4-hydroxy-2-oxo-1,2-dihydroquinoline-3-carboxamide* (**11g**): the compound was synthesized according to the general procedure, starting from cinnamoyl chloride (**10h**) (181.5 mg, 1.09 mmol) and carboxamide **7** (200.0 mg, 0.73 mmol), in 1.5 mL of THF and 0.1 mL of Et_3_N. After the work-up procedure, the product was obtained upon recrystallization from methanol as a white solid. Yield: 90%; m.p. 157-162 ^o^C; ^1^H NMR (600 MHz, DMSO-*d*_6_) *δ* (ppm) 17.33 (s, 1H, OH), 10.43 (br, 1H, CON**H**CH_2_), 8.34 (br, 1H, CH_2_NHCO), 8.10 (d, *J* = 8.4 Hz, 1H, Ar-H), 7.80 (t, *J* = 7.8 Hz, 1H, Ar-H), 7.66 (d, *J* = 8.4 Hz, 1H, Ar-H), 7.56 (d, *J* = 7.2 Hz, 2H, Ar-H), 7.44 (d, *J* = 15.6 Hz, 1H, H-16), 7.38 (m, 3H, Ar-H), 6.63 (d, *J* = 15.6 Hz, 1H, H-15), 4.29 (q, *J* = 7.2 Hz, 2H, NCH_2_CH_3_), 3.54 (br, 2H, H-11), 3.42 (br, 2H, H-12), 1.21 (t, *J* = 7.2 Hz, 3H, NCH_2_CH_3_); ^13^C NMR (150 MHz, DMSO-*d_6_*) *δ* ppm 171.09, 170.99, 165.34, 161.16, 138.82, 138.66, 134.85, 134.33, 129.47, 128.93, 127.54, 124.78, 122.36, 122.00, 115.34, 115.03, 95.95, 38.39, 36.66, and 12.76; HR-MS *m*/*z* (neg): 405.1689 C_23_H_22_O_4_N_3_ (calcd. 404.16068).

#### 3.1.8. Synthesis of 4-Hydroxy-1-methyl-3-nitroquinolin-2(1H)-one (**13**)

HNO_3_ (70%, 9 mmol) is added dropwise to a suspension of 4-hydroxyquinolin-2(1H)-one (**12**) (6 mmol) in glacial acetic acid (10 mL). The mixture was heated at 90 °C for 1–2 h and then cooled to r.t. The solid was collected through vacuum filtration and washed with Et_2_O (3 × 15 mL). Characterization data of the compound were identical to those reported in the literature [76]; Yield: 87%; m.p. 159–161 °C; ^1^H NMR (300 MHz, CDCl_3_) *δ* (ppm) 13.69 (s, 1H, -OH), 8.28 (d, *J* = 7.0 Hz, 1H, H-5), 7.81 (t, *J* = 7.2 Hz, 1H, H-7), 7.36 (m, 2H, *J* = 7.0 Hz, H-6/8), 3.70 (s, 3H, N-CH_3_); ^13^C NMR (CDCl_3_, 75 MHz) *δ* (ppm) 165.2, 154.8, 140.8, 136.4, 126.8, 123.1, 114.6, 113.1, 99.2, and 29.6.

#### 3.1.9. Synthesis of the 3-Amino-4-hydroxy-1-methylquinolin-2(1H)-one Hydrochloride (**14**)

*4-hydroxy-1-methyl-3-nitroquinolinone* (**13**) is added in equimolar amount of aqueous NaOH 1M, and the mixture was stirred until homogeneous. An equimolar amount of sodium dithionite (Na_2_S_2_O_4_) was then added, and the reaction mixture was stirred for 30 min in r.t., while the reaction was monitored by a thin-layer chromatography. After the reaction’s completion, the mixture was acidified with an aqueous HCl 10% in an ice-water bath, and the precipitated solid was collected through vacuum filtration. The product was used in the next step without any further purification. Yield: 96%; m.p. > 250 °C; ^1^H NMR (CDCl_3_/DMSO-*d*_6_, 300 MHz) *δ* (ppm) 7.71 (d, *J* = 7.0 Hz, 1H, Ar-H), 7.19 (t, *J* = 7.8 Hz, 1H, Ar-H), 7.96 (d, *J* = 7.0 Hz, 1H, Ar-H), 6.86 (t, *J* = 7.8 Hz 1H, Ar-H), 3.25 (s, 3H, N-CH_3_ ), 4.70 (s, 2H, ΝH_2_.HCl).

#### 3.1.10. General Procedure for the Preparation of Reverse Amides **16a**–**16d**

The synthesized hydrochloric salt (**14**) was added to a round bottom flask containing THF solvent, and the mixture was stirred for 15 min. An appropriate amount of anhydrous Et_3_N and the corresponding chloride were then added, and the reaction mixture was refluxed at 52 °C for 2–3 h under an inert atmosphere. The reaction is monitored by TLC. After the reaction was complete, a small amount of water was added to the mixture and then acidified with aqueous HCl 10% in an ice-water bath, and the solid precipitated was collected through vacuum filtration. Product **16** was obtained as a solid, and if required, it was further purified by recrystallization from methanol. 

*N-(4-hydroxy-1-methyl-2-oxo-1,2-dihydroquinolin-3-yl)butyramide* (**16a**): the compound was synthesized according to the general procedure, starting from 3-amino-4-hydroxy-1-methylquinolin-2(1H)-one hydrochloride (**14**) (200 mg, 1.7 mmol) in 5 mL THF and proceeding with anhydrous Et_3_N (0.3 mL, 4.25 mmol) and butyryl chloride (**15a**) (266.4 mg, 2.50 mmol). The reaction mixture was refluxed for 2.5 h. After the work-up procedure, the product was obtained without any further purification. Yield: 46%; m.p. 122–123 °C; ^1^H NMR (CDCl_3_, 600 MHz) *δ* (ppm) 12.90 (s,1H, OH), 8.72 (s, 1H, NH), 8.15 (dd, *J* = 7.8, 1.2 Hz, 1H, Ar-H), 7.57 (ddd, *J* = 8.5, 7.3, 1.4 Hz, 1H, Ar-H), 7.32 (m, 2H, Ar-H), 3.75 (s, 3H, N-CH_3_), 2.51 (t, *J* = 7.2 Hz, 2H, COCH_2_), 1.81 (sextep, *J* = 7.2 Hz, 2H, CH_2_CH_2_CH_3_), 1.04 (t, *J* = 7.2 Hz, 3H, CH_2_CH_3_); ^13^C NMR (CDCl_3_, 150 MHz) *δ* (ppm) 173.7, 159.5, 148.8, 136.5, 130.3, 124.6, 122.5, 117.2, 113.6, 109.2, 38.7, 29.9, 19.2, and 13.5; ESI-MS (*m*/*z*): 261.1 [M + H]^+^, 283 [M + 23]^+^, 191 [M − 69]^+^.

*N-(4-hydroxy-1-methyl-2-oxo-1,2-dihydroquinolin-3-yl)-4-methylbenzamide* (**16b**): the compound was synthesized according to the general procedure, starting from 3-amino-4-hydroxy-1-methylquinolin-2(1H)-one hydrochloride (**14**) (490 mg, 2.16 mmol) in 12 mL THF and proceeding with anhydrous Et_3_N (0.73 mL, 5.4 mmol) and 4-methyl-benzoyl chloride (**15b**) (400.4 mg, 2.59 mmol). The reaction mixture was refluxed for 2 h. After the work-up procedure, the product was obtained without further purification. Yield: 30%; m.p. 188–190 °C; ^1^H NMR (CDCl_3_, 300 MHz) *δ* (ppm) 12.83 (s, 1H, OH), 9.27 (s, 1H, NH), 7.85 (dd, *J* = 6.0, 3.0 Hz, 1H, Ar-H), 7.59 (d, *J* = 6.0 Hz, 2H, H-12 & H-16), 7.31 (t, *J* = 9.0 Hz, 1H, Ar-H), 7.11 (d, *J* = 9.0 Hz, 1H, Ar-H), 7.14 (d, *J* = 9.0 Hz, 1H, Ar-H), 7.04 (br, 1H, Ar-H), 7.05 (d, *J* = 6.0 Hz, 2H, H-13 & H-15), 3.51 (s, 3H, N-CH_3)_, 2.19 (s, 3H, Ar-CH_3_); ^13^C NMR (CDCl_3_, 75 MHz) *δ* (ppm) 166.8, 159.5, 152.3, 142.8, 137.9, 131.4, 130.5, 129.6, 128.4, 123.9, 122.5, 116.5, 115.1, 109.3, 29.9, and 21.5; ESI-MS (*m*/*z*): 309.1 [M + H]^+^, 219 [M − 90]^+^.

*2-hydroxy-N-(4-hydroxy-1-methyl-2-oxo-1,2-dihydroquinolin-3-yl)benzamide* (**16c**): the compound was synthesized according to the general procedure, starting from 3-amino-4-hydroxy-1-methylquinolin-2(1H)-one hydrochloride (**14**) (490 mg, 2.16 mmol) in 12 mL THF and proceeding with anhydrous Et_3_N (0.73 mL, 5.4 mmol) and 2-hydroxy-benzoyl chloride (**10d**) (405.5 mg, 2.59 mmol). The reaction mixture was refluxed for 3 h. After the work-up procedure, the product was obtained upon recrystallization from methanol. Yield: 42%; m.p. > 250 °C; ^1^H NMR (DMSO, 300 MHz) *δ* (ppm) 13.28 (s, 1H, Ar-OH), 11.58 (s, 1H, OH), 9.74 (s, 1H, NH), 8.03 (m, 2H, Ar-H), 7.63 (br, 1H, Ar-H), 7.50 (m, 2H, Ar-H), 7.33 (br, 1H, Ar-H), 7.03 (m, 2H, Ar-H), 3.69 (s, 3H, N-CH_3_); ^13^C NMR (DMSO, 75 MHz) *δ* (ppm): 165.2, 159.0, 157.2, 148.9, 136.9, 134.6, 134.5, 131.0, 130.7, 123.7, 122.3, 119.7, 117.0, 116.4, 114.7, 109.6, and 29.7; ESI-MS (*m*/*z*): 309.1 [Μ + H]^+^, 291.3 [Μ − 17]^+^, 215 [Μ − 93]^+^.

### 3.2. Biological In Vitro Assays

#### 3.2.1. Determination of the Reducing Activity of DPPH Radical 

The assay for the determination of the reducing activity of DPPH radical was performed according to the methods of Hadjipavlou-Litina et al., which we have also used in our previous works [65,67,77]. Τhe results presented in Table 1 were averaged and compared with the appropriate standard nordihydroguaiaretic acid (NDGA).

#### 3.2.2. Inhibition of Linoleic Acid Lipid Peroxidation

The assay for the determination of the inhibition of linoleic acid peroxidation induced by the free radical initiator 2,20-Azobis(2-amidinopropane) dihydrochloride (AAPH) was performed according to the methods of Hadjipavlou-Litina et al., which we have also used in our previous works [65,67,77]. 

#### 3.2.3. Competition of the Tested Compounds with DMSO for Hydroxyl Radicals

The assay was performed according to the methods of Pontiki et al. Trolox was used as a reference compound [78].

#### 3.2.4. ABTS^+^—Decolorization Assay for Antioxidant Activity

The experimental technique used in this section was performed according to the methods of Pontiki et al. [78]. The results were compared to the appropriate standard inhibitor Trolox.

#### 3.2.5. Non-Enzymatic Assay of Superoxide Radicals Measurement of Superoxide Radical Scavenging Activity

The experimental technique used for this assay was performed according to our previous works. Caffeic acid was used as an appropriate standard [61,70].

#### 3.2.6. Soybean LOX Inhibition Study In Vitro

The assay for the determination of the inhibition of soybean LOX was performed according to the methods of Hadjipavlou-Litina et al., which we have also used in our previous works [65,67,77]. 

#### 3.2.7. Computational Methods–Molecular Docking Studies on Soybean Lipoxygenase

For the docking studies, soybean lipoxygenase (PDB ID: 3PZW) was used, and the visualization was accomplished through UCSF Chimera (resource for Biocomputing, Visualization, and Informatics at the University of California, San Francisco, CA, USA) [79] and Free Maestro [75]. The protein was prepared: water molecules were removed, missing residues were added with Modeller (10.3) (Departments of Biopharmaceutical Sciences and Pharmaceutical Chemistry, and California Institute for Quantitative Biomedical Research, Mission Bay Byers Hall, University of California San Francisco, San Francisco, CA 94143, USA), hydrogen atoms and AMBER99SB-ILDN charges were added, and the charge on iron was set to +2.0, with no restraint applied to the iron atom and the ligands [80]. Open-Babel (3.1.1) was used to generate and minimize ligand 3D coordinates using the MMFF94 force field [81]. Ligand topologies and parameters were generated by ACPYPE (Ante-ChamberPYthon Parser interfacE) (24 December 2021) [82] using Antechamber (AmberTools 22.10) [83]. Energy minimizations were carried out using the AMBER99SB-ILDN force field [84] with GROMACS (4.6.5). Docking was performed with AutoDockVina (1.2.3) applying a grid box of size 100 Å, 70 Å, 70 Å in the x, y, z dimensions [85]. The generation of docking input files and the analysis of the docking results was accomplished by UCSF-Chimera. Docking was carried out with an exhaustiveness value of 10 and a maximum output of 20 docking modes.

## 4. Conclusions

In conclusion, this work reports the synthesis of three sets of compounds, which share the privileged structural framework of 4-hydroxy-2-quinolinone as a common feature. Among the synthesized analogues, eighteen quinolinone–carboxamide derivatives (**3a**–**3e**, **3g**–**3k**, **3n**–**3s**, **3u**, and **7**), all the seven hybrid compounds (**11a**–**11g**) and two of the reverse amides (**16b** and **16c**), to our knowledge have not been reported in the literature. In order to investigate the multi-target character of the compounds, we evaluated their antioxidant profile via five in vitro tests, as well as their ability to inhibit soybean LOX, as an indication of their anti-inflammatory activity. In this way, we tried to build a structure–activity relationship and determine how the final biological effects are influenced in relation to the different substituents attached to the nitrogen of quinolinone moiety and to the amide bond. Results revealed carboxamides **3h** and **3s** as the most potent LOX inhibitors (IC_50_ = 10 μM). Both could be used as lead compounds for further rational design. The **3g** analogue is the compound with the best-combined activity, exhibiting good antioxidant and anti-inflammatory activity (LOX inhibition IC_50_ = 27.5 μM, 100% inhibition of lipid peroxidation, 67.7% ability to scavenge hydroxyl radicals and 72.4% in ABTS radical cation decolorization assay). The in vitro results were supported by the in silico studies on soybean LOX for the most potent synthesized quinolinone–carboxamides (**3h** and **3s**), indicating interactions in an alternative binding site than the catalytic site already validated by the binding mode of NDGA, a potent well known LOX inhibitor. 

## Figures and Tables

**Table 1:** In vitro antioxidant evaluation of all the synthesized compounds via the DPPH scavenging ability and the inhibition of linoleic acid assays.

Compound	Interaction with the Free Radical DPPH (%)		Inhibition of Lipid Peroxidation of Linoleic Acid Induced by AAPH Radical (%) 100 μM
	100 μM 20 min	100 μM 60 min	
**3a**	2.4	12.4	100
**3b**	2.7	11.8	100
**3c**	3.3	10.7	100
**3d**	15.9	20.8	32.8
**3e**	6.6	12.2	no
**3f**	39.3	48.6	100
**3g**	40.7	51.7	100
**3h**	6.0	8.1	31
**3i**	5.2	3.7	36
**3j**	32.5	46.5	32.8
**3k**	1.4	11.8	41.4
**3l**	2.6	2.6	no
**3m**	3.1	1.2	11.3
**3n**	13.9	19.2	100
**3o**	20.8	8.0	100
**3p**	19.5	6.6	100
**3q**	23.8	10.0	100
**3r**	12.1	1.2	17.1
**3s**	21.2	8.1	28.9
**3t**	19.7	9.5	100
**3u**	19.3	8.3	16.4
**11a**	1.0	4.4	89.0
**11b**	3.0	no	93.8
**11c**	2.0	2.0	91.0
**11d**	3.0	2.0	94.0
**11e**	3.0	5.0	97.0
**11f**	2.0	4.0	93.0
**11g**	no	2.0	97.0
**16a**	24.0	31.0	83.0
**16b**	27.0	32.0	88.0
**16c**	25.0	28.0	88.0
NDGA	83.0	94.0	
Trolox			88.0

no: no action under the experimental conditions.

**Table 2:** Antioxidant activity of carboxamide derivatives via their ability to scavenge hydroxyl radicals and the ABTS radical cation decolorization assay.

Compound	OH% 0.1 mM	ABTS% 0.1 mM	PMS% 0.1 mM
**3a**	39.3	33.8	23.1
**3b**	40.8	35.5	84.6
**3c**	39.8	25.6	76.9
**3d**	no	no	66.6
**3e**	86.1	2.6	66.6
**3f**	67.7	77.3	no
**3g**	67.7	72.4	no
**3h**	78.6	no	no
**3i**	78.1	no	no
**3j**	100	49.7	no
**3k**	no	54.5	61.5
**3l**	53.7	no	no
**3m**	94.0	12.4	no
**3n**	14.9	no	no
**3o**	44.3	20.6	no
**3p**	16.9	no	no
**3q**	98.0	no	no
**3r**	50.7	no	no
**3s**	100	no	no
**3t**	no	no	no
**3u**	10.3	no	no
Trolox	82.0	93.0	
Caffeic acid			23

no: no action under the experimental conditions.

**Table 3:** In vitro determination of soybean LOX inhibition activity.

Compound	Inhibition of Soybean Lipoxygenase	
	% at 0.1 mM	IC_50_ (μM) *
**3a**		60.0
**3b**	45.0	
**3c**		52.0
**3d**	no	
**3e**	no	
**3f**		49.0
**3g**		27.5
**3h**		10.0
**3i**	no	
**3j**		100.0
**3k**		315.0
**3l**		45.0
**3m**	15.0	
**3n**		85.0
**3o**	no	
**3p**	no	
**3q**	24.0	
**3r**	37.4	
**3s**		10.0
**3t**	no	
**3u**	no	
**11a**		61.0
**11b**		70.0
**11c**		57.5
**11d**	5.0	
**11e**		52.5
**11f**		70.0
**11g**		85.5
**16a**		81.0
**16b**		100.0
**16c**		82.5
NDGA		0.45

*: IC_50_ was calculated for the compounds that exhibited promising inhibition percentage at 0.1 mM; no: no action under experimental conditions.

## Data Availability

Data are contained within the article and Supplementary Materials.

## Supplementary Materials

The following supporting information can be downloaded at: https://www.mdpi.com/article/10.3390/molecules29010190/s1: The ^1^H-NMR spectra of all the synthesized compounds and ^13^C-NMR and HRMS spectra of the novel synthesized compounds.
