# Supplementary material for: Novel Multi-Target Agents Based on the Privileged Structure of 4-Hydroxy-2-quinolinone
Source: Molecules. 2023 Dec 28;29(1):190. doi: 10.3390/molecules29010190 (PMC10780633; doi:10.3390/molecules29010190)
Supplement: Supplementary file 1 [file molecules-29-00190-s001.zip › molecules-2700691-supplementary.pdf]

Novel multi-target agents based on the privileged structure of 4-hydroxy-2-quinolinone

Molecules, Special Issue: "Design and Synthesis of Novel Anti-Inflammatory Agents"

Ioanna Kostopoulou [1], Andromachi Tzani [1], Konstantina Chronaki [1], Kyriakos C. Prousis [2], Eleni Pontiki [3], Dimitra Hadjiplavlou-Litina [3], and Anastasia Detsi [1,*]

1. Laboratory of Organic Chemistry, Department of Chemical Sciences, School of Chemical Engineering, National Technical University of Athens, Heroon Polytechniou 9, Zografou Campus, 15780 Athens, Greece; ioanna.th.kostopoulou@gmail.com (I.K.); atzani@mail.ntua.gr (A.T.); konstantina163264@hotmail.gr (K.C.);
2. Institute of Chemical Biology, National Hellenic Research Foundation, 48 Vassileos Constantinou Avenue, 11635 Athens, Greece; kyrprous@eie.gr (K.C.P.)
3. Laboratory of Pharmaceutical Chemistry, School of Pharmacy, Faculty of Health Sciences, Aristotle University of Thessaloniki, 54124 Thessaloniki, Greece; epontiki@pharm.auth.gr (E.P.); hadjipav@pharm.auth.gr (D.H.-L.)

*Correspondence: Correspondence: adetsi@chemeng.ntua.gr; Tel.: +30-210-7724126

¹H NMR of compound 3a

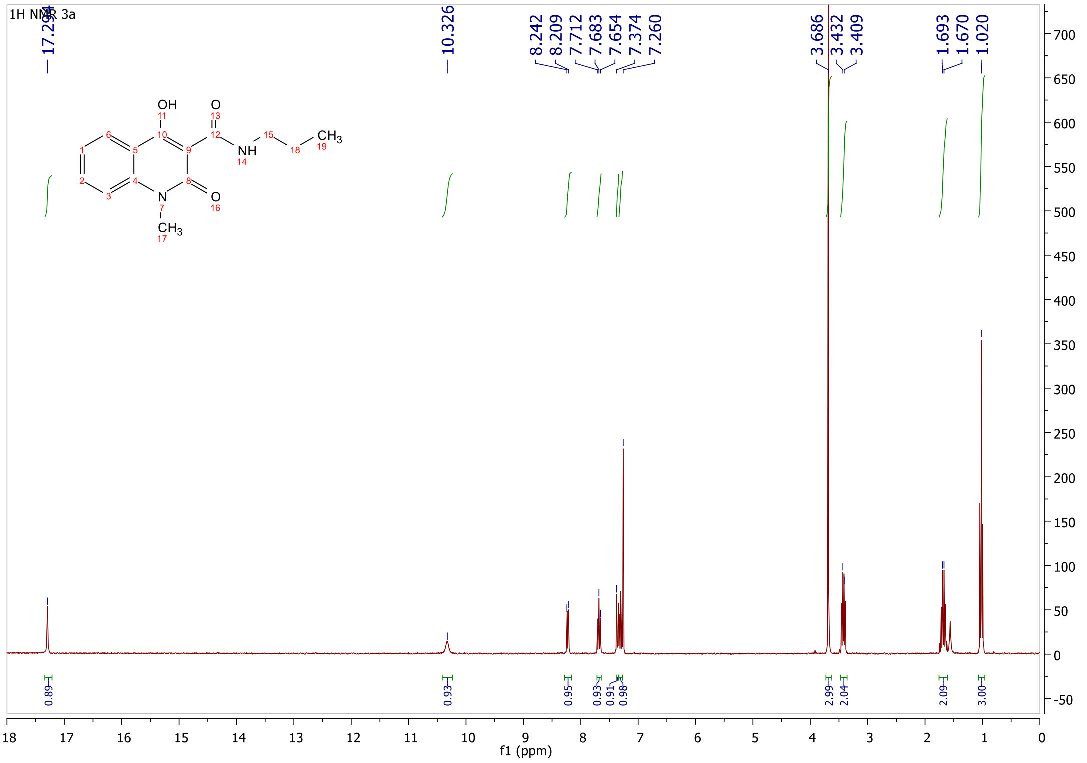

¹H NMR of compound 3b

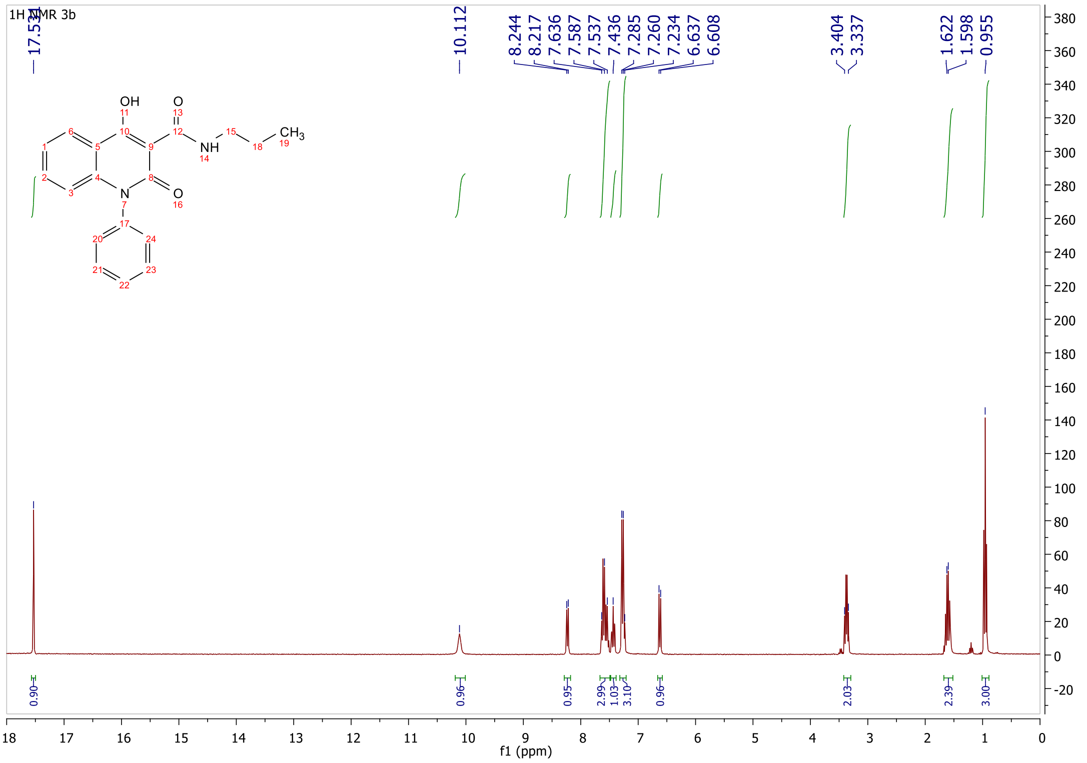

¹H NMR of compound 3c

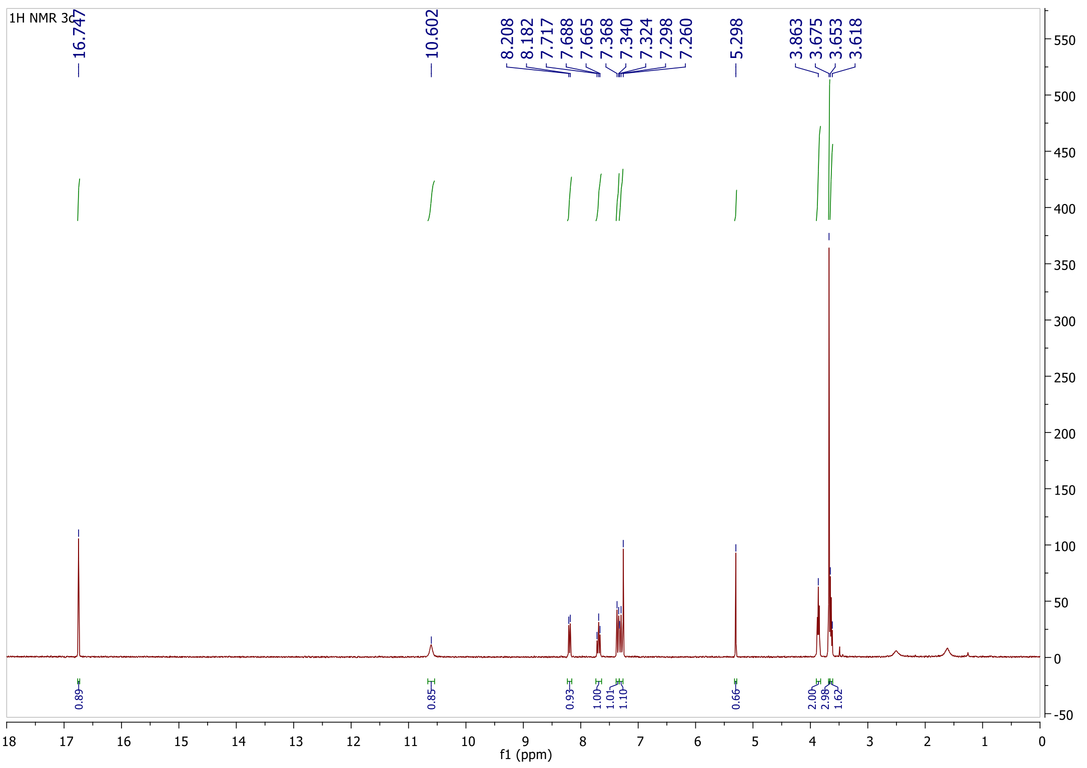

¹H NMR of compound 3d

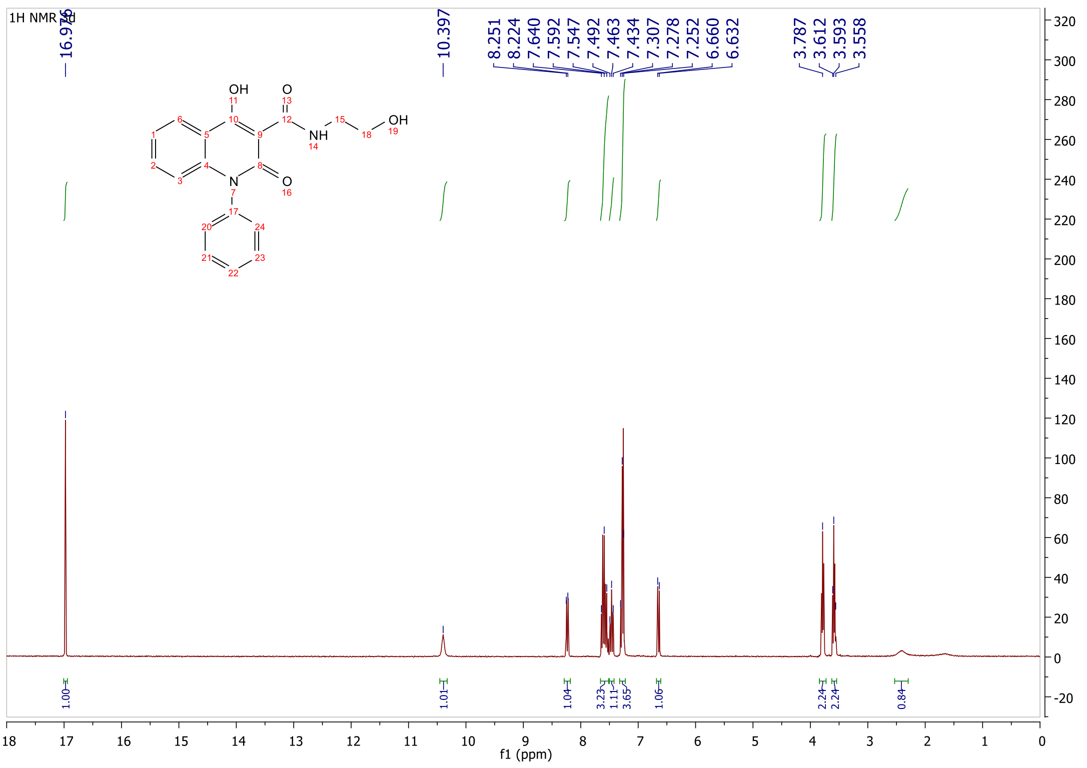

^{1}H NMR of compound 3e

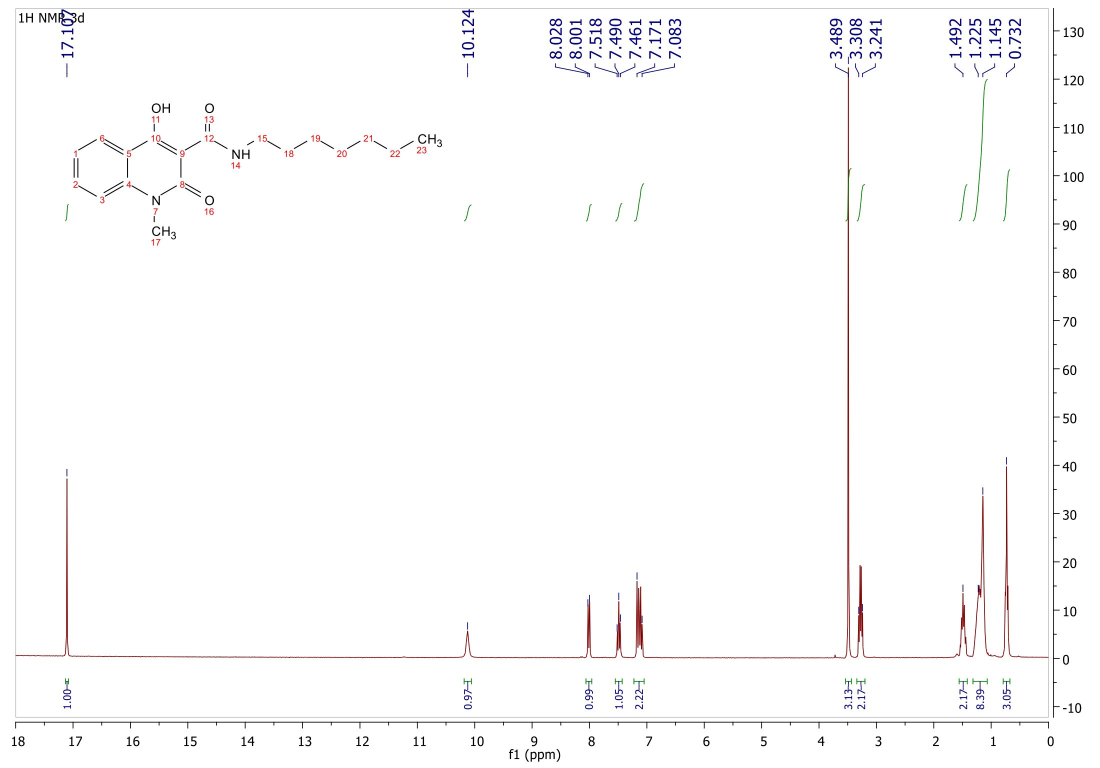

^{1}H NMR of compound 3f

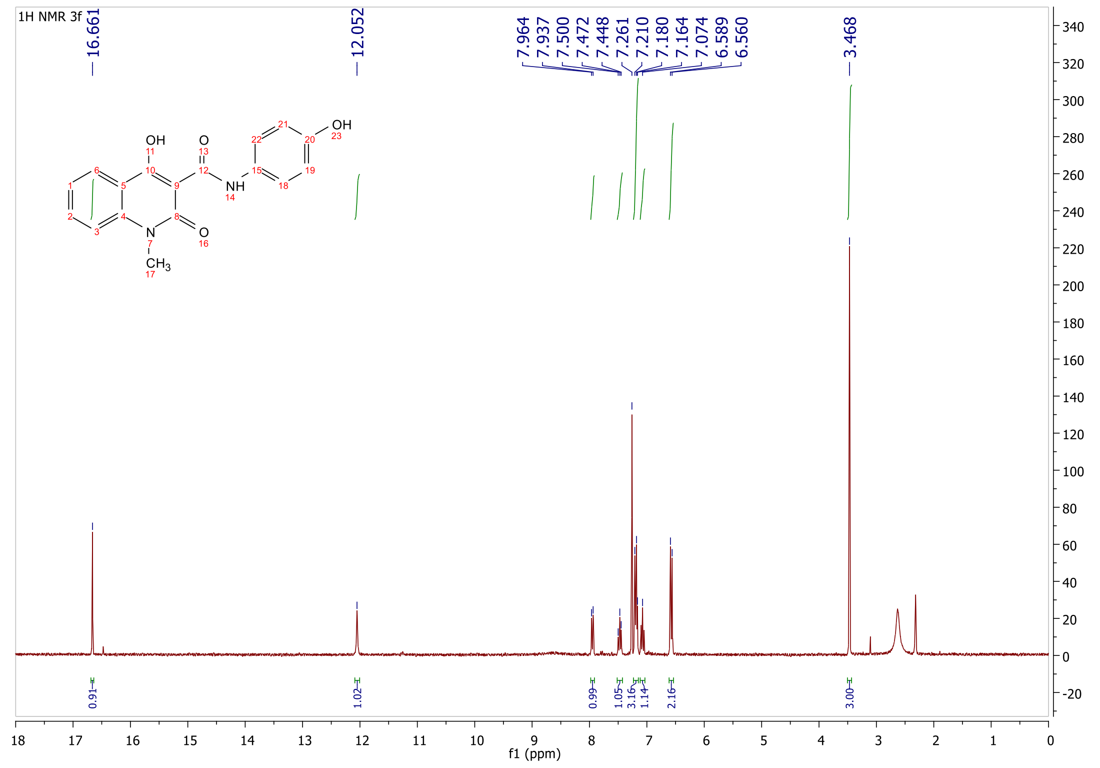

¹H NMR of compound 3g

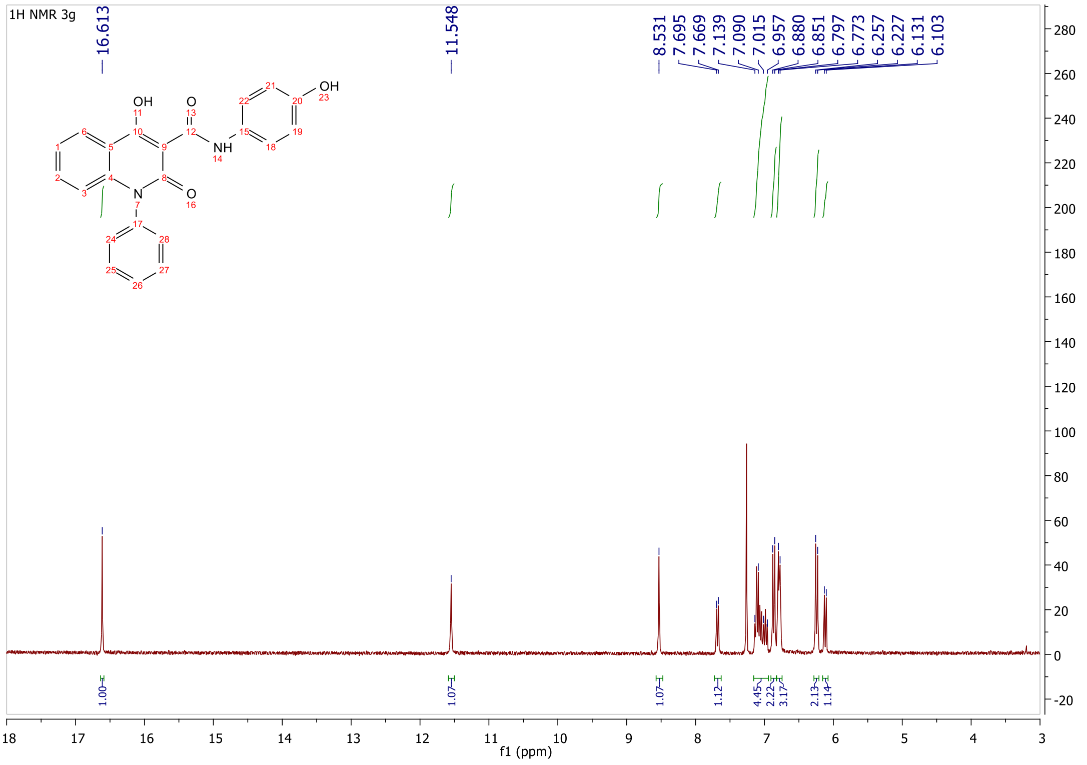

¹H NMR of compound 3h

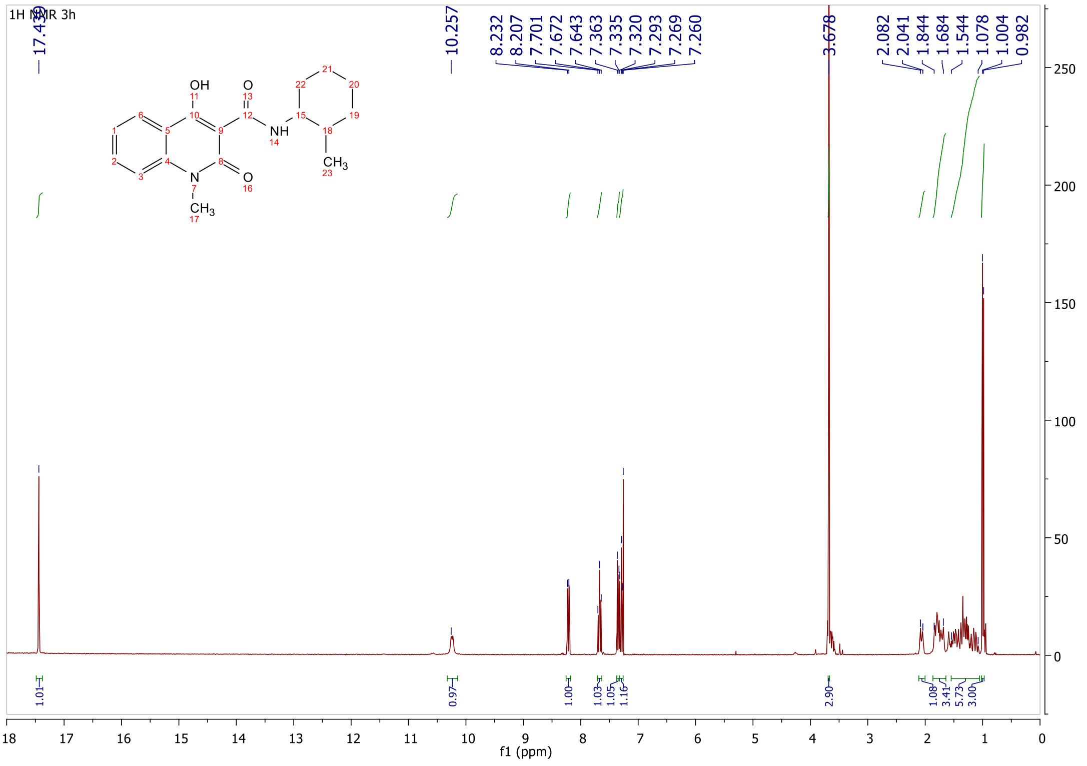

¹H NMR of compound 3i

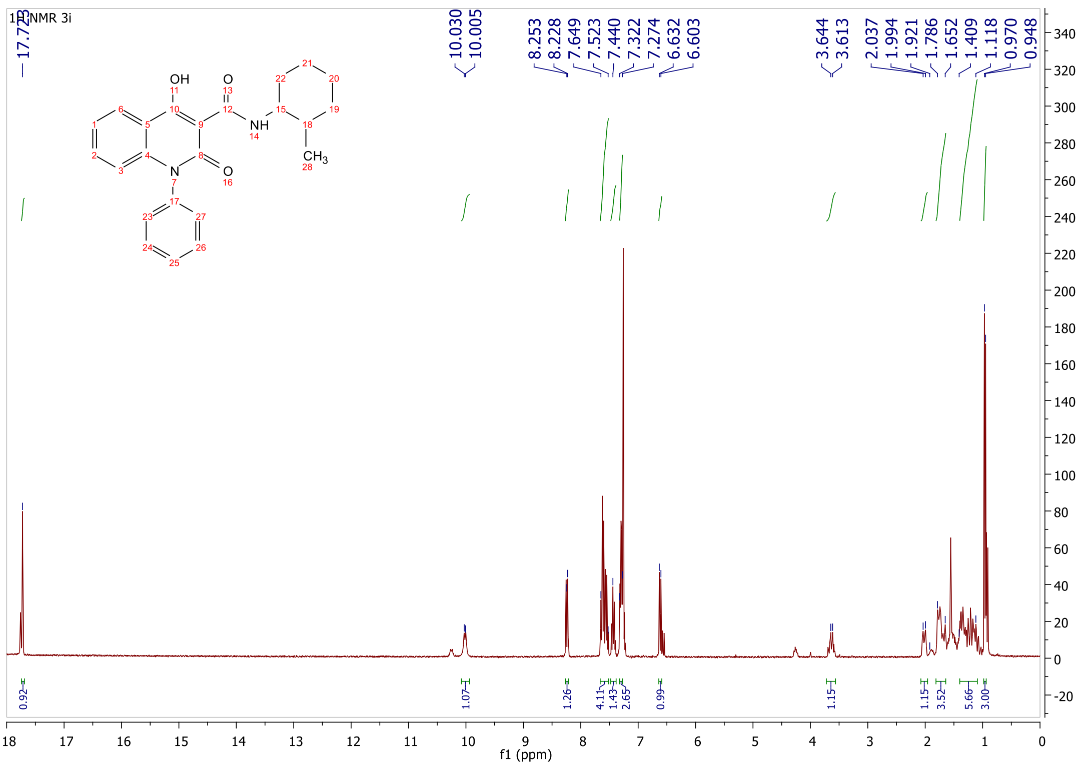

¹H NMR of compound 3j

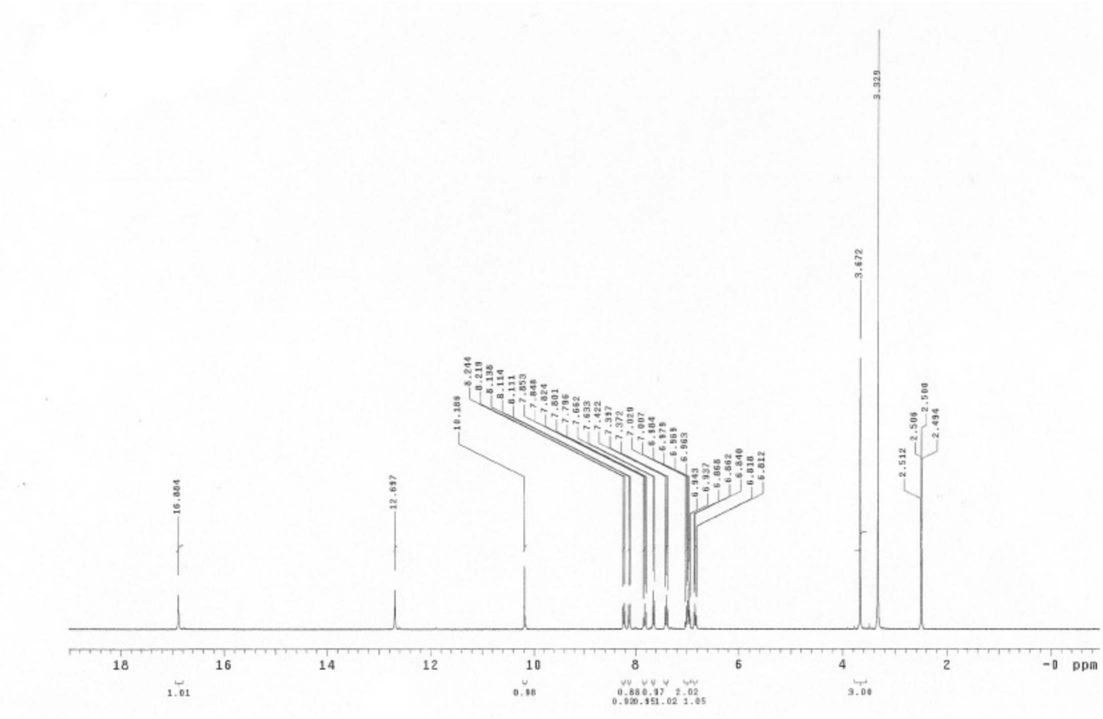

^{1}H NMR of compound 3k

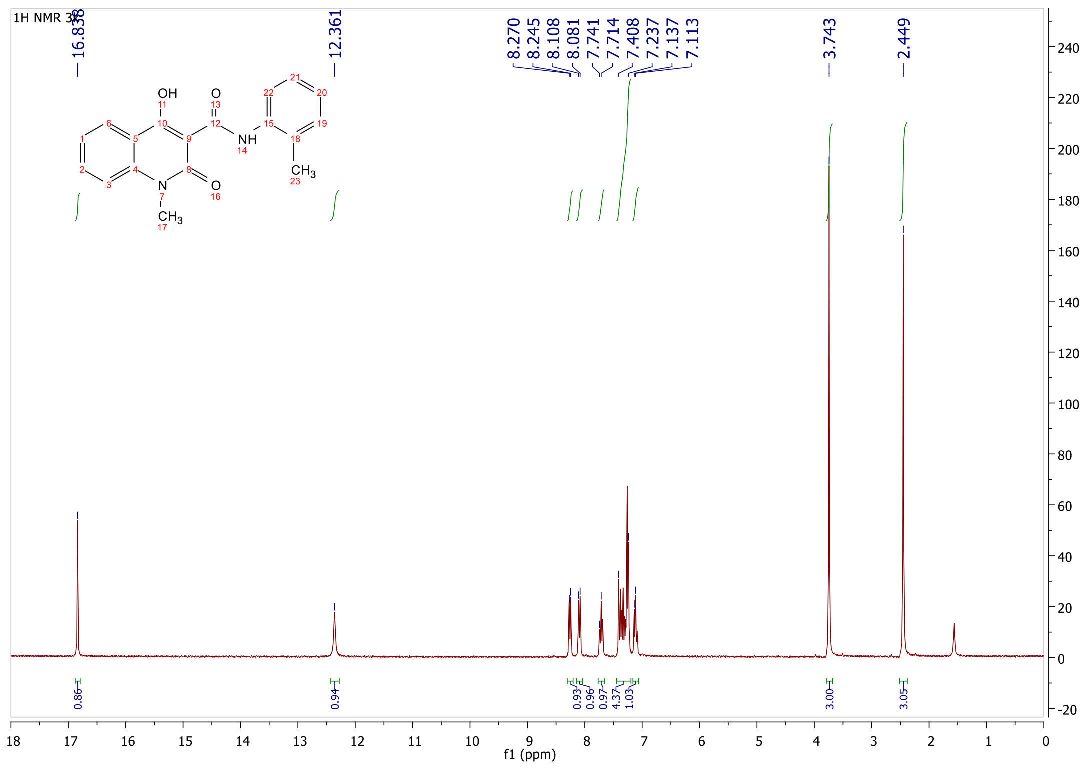

^{1}H NMR of compound 3l

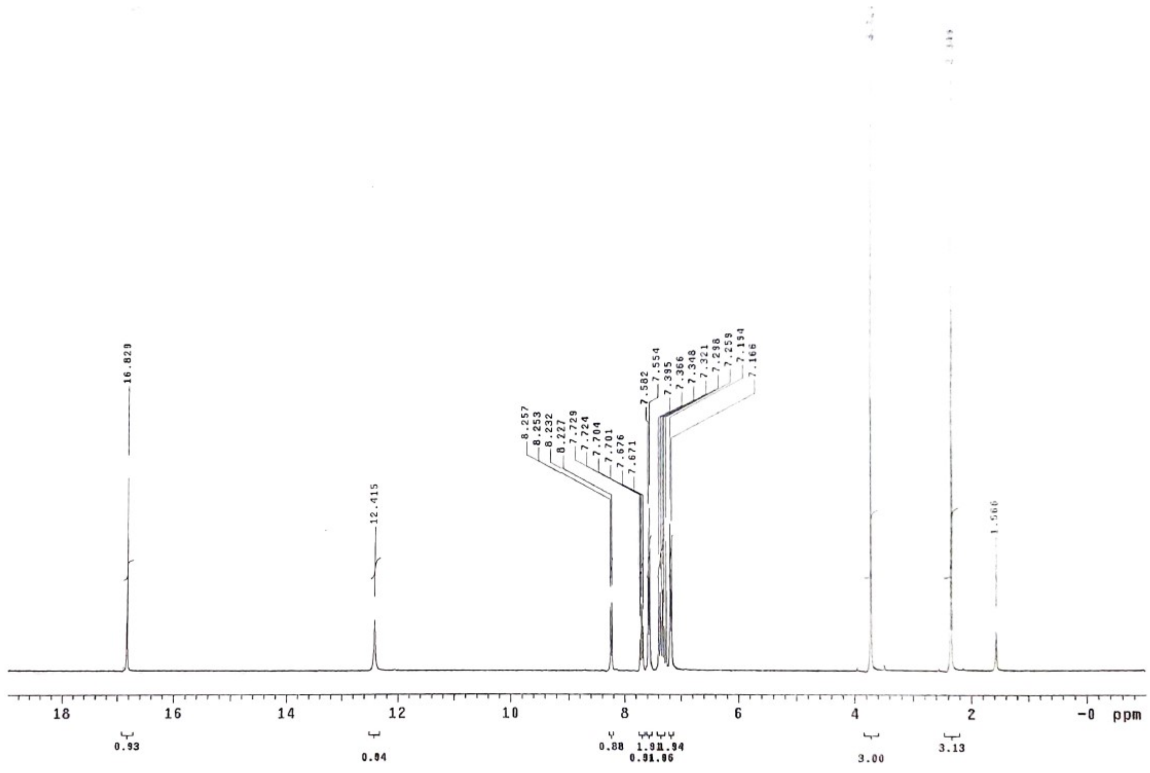

^{1}H NMR of compound 3m

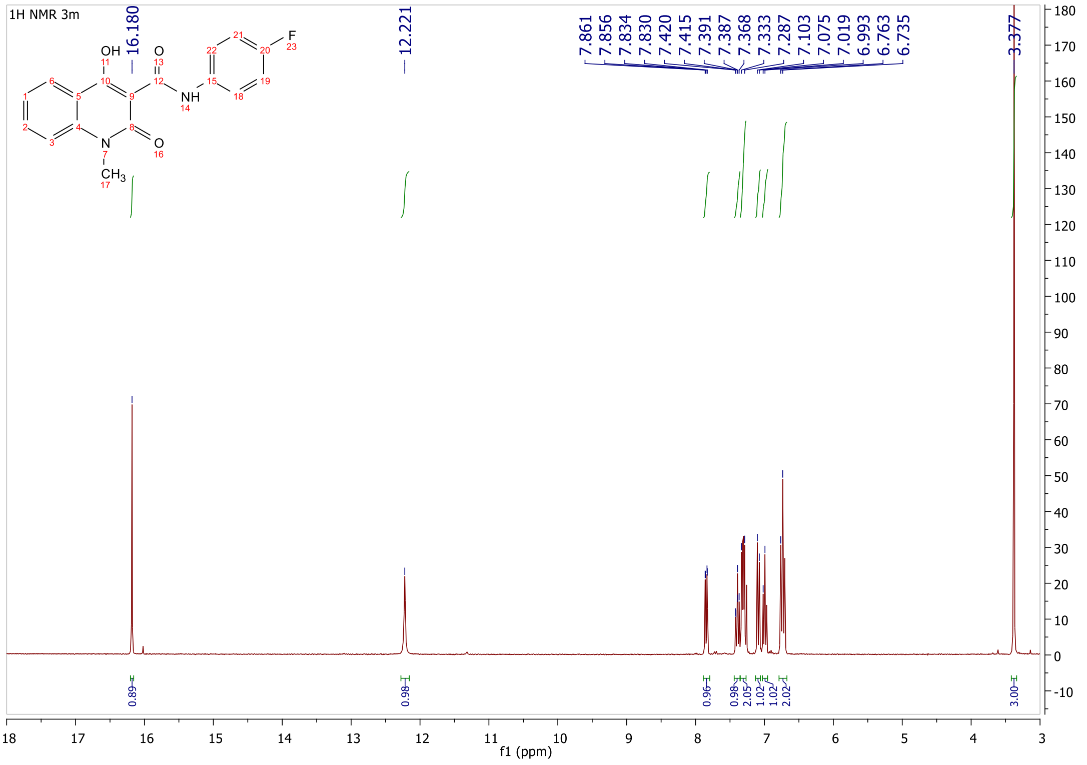

^{1}H NMR of compound 3n

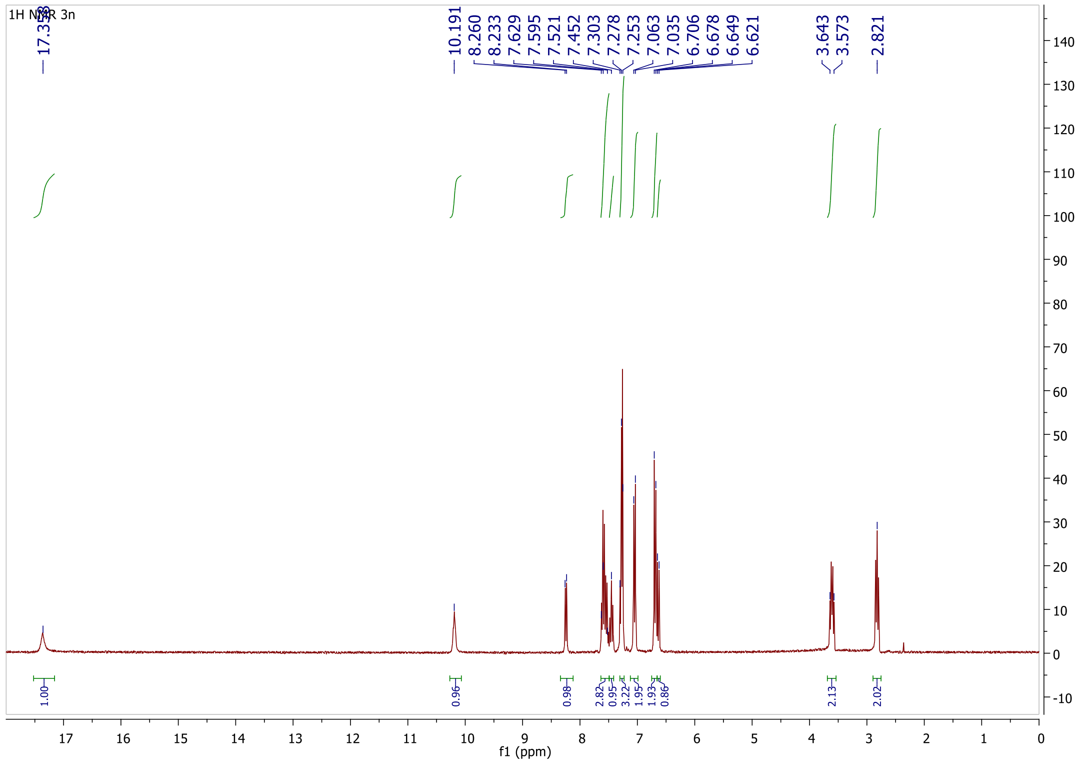

^{1}H NMR of compound 3o

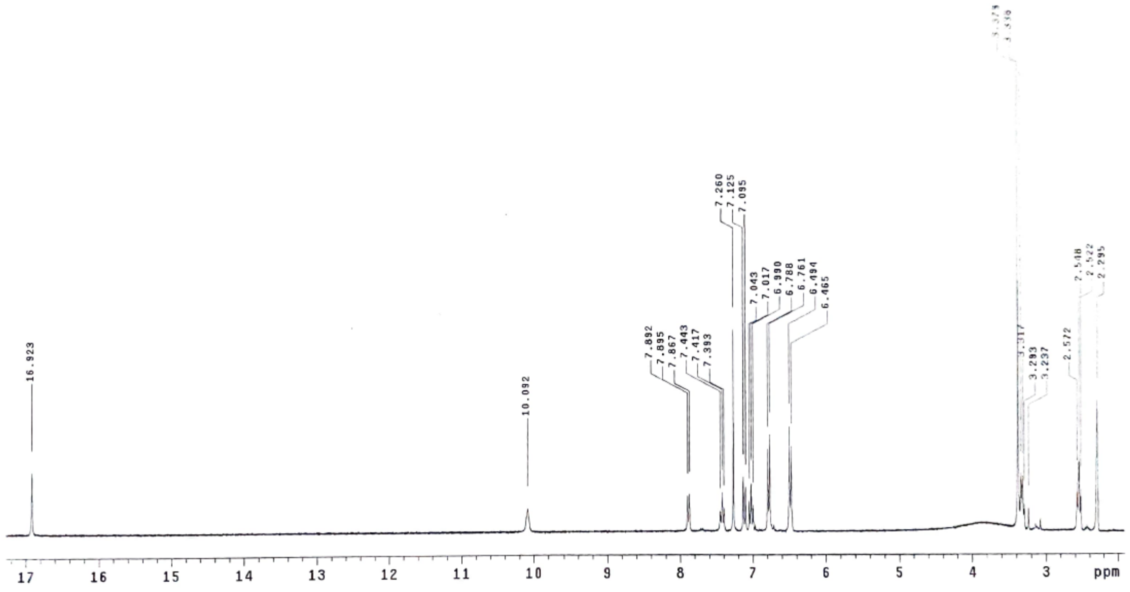

^{1}H NMR of compound 3p

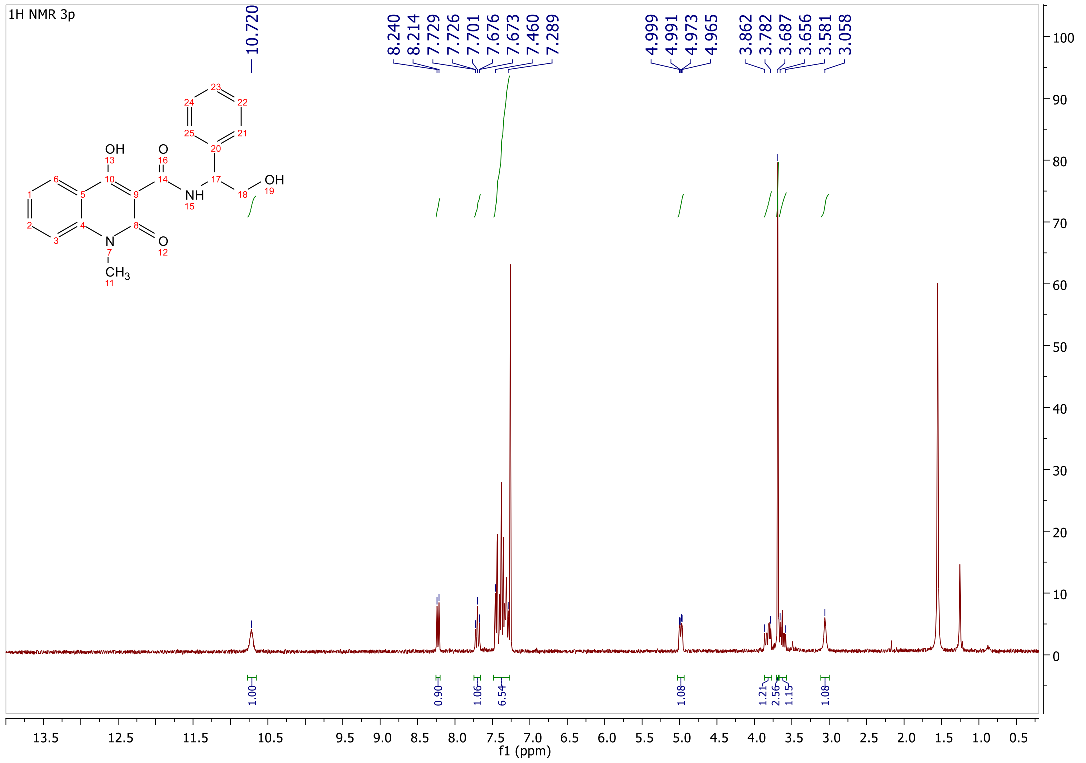

^{1}H NMR of compound 3q

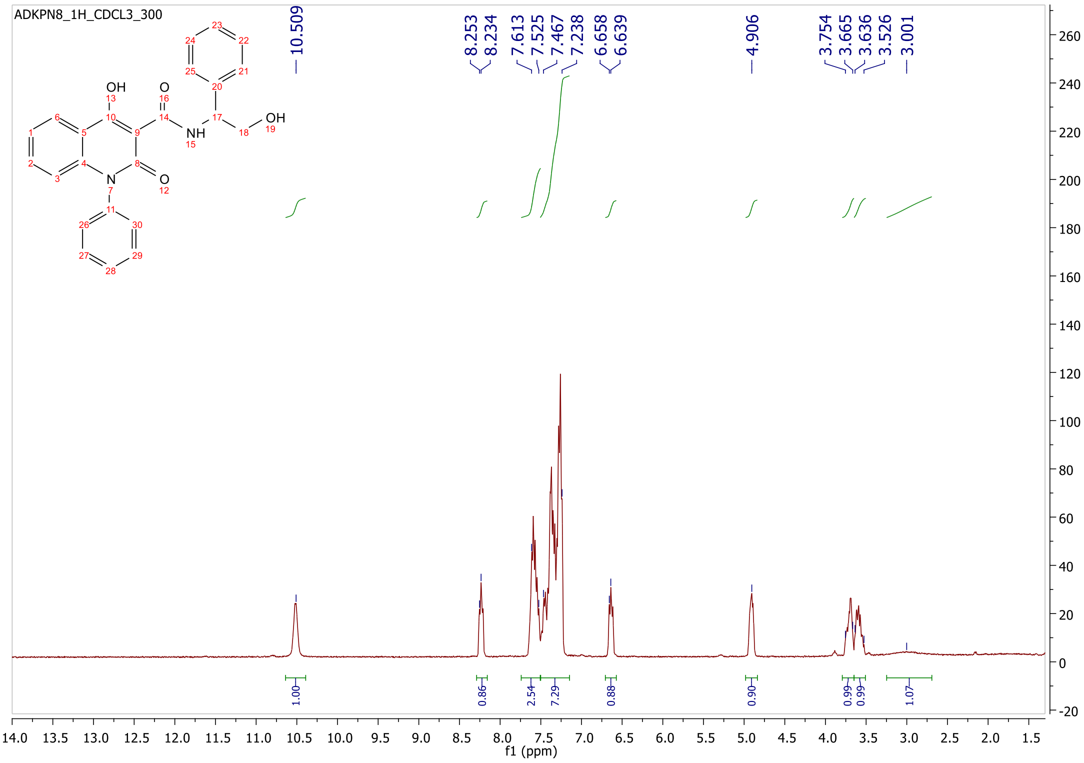

^{1}H NMR of compound 3r

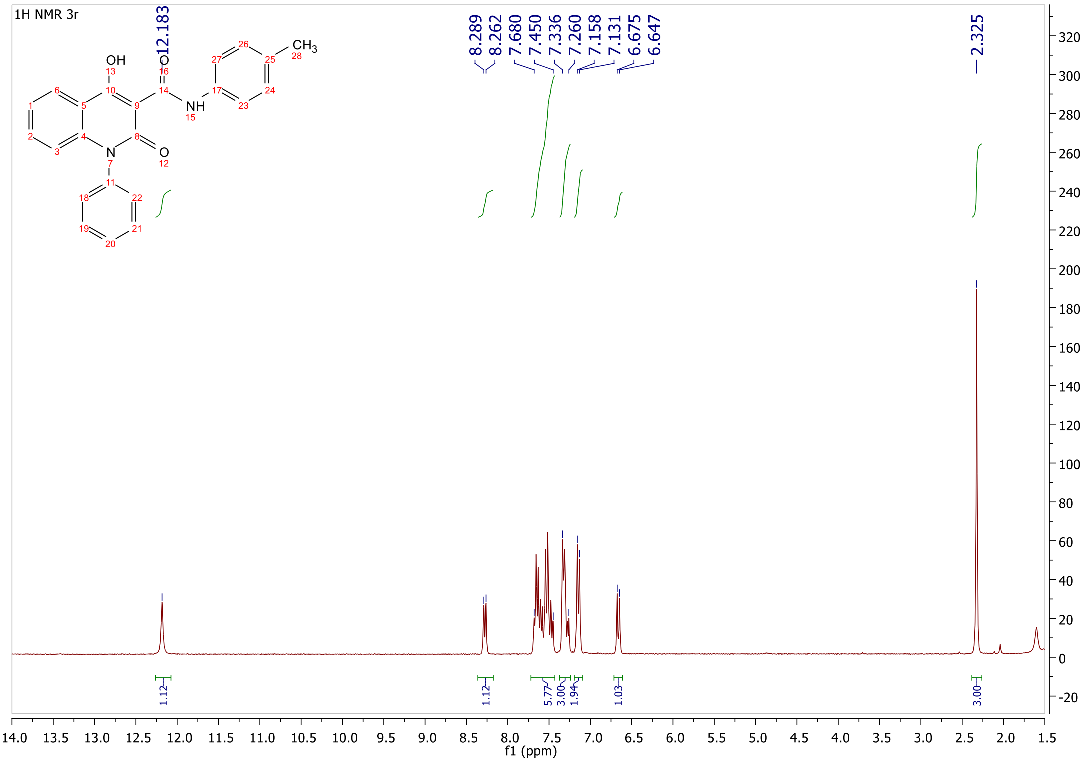

^{1}H NMR of compound 3s

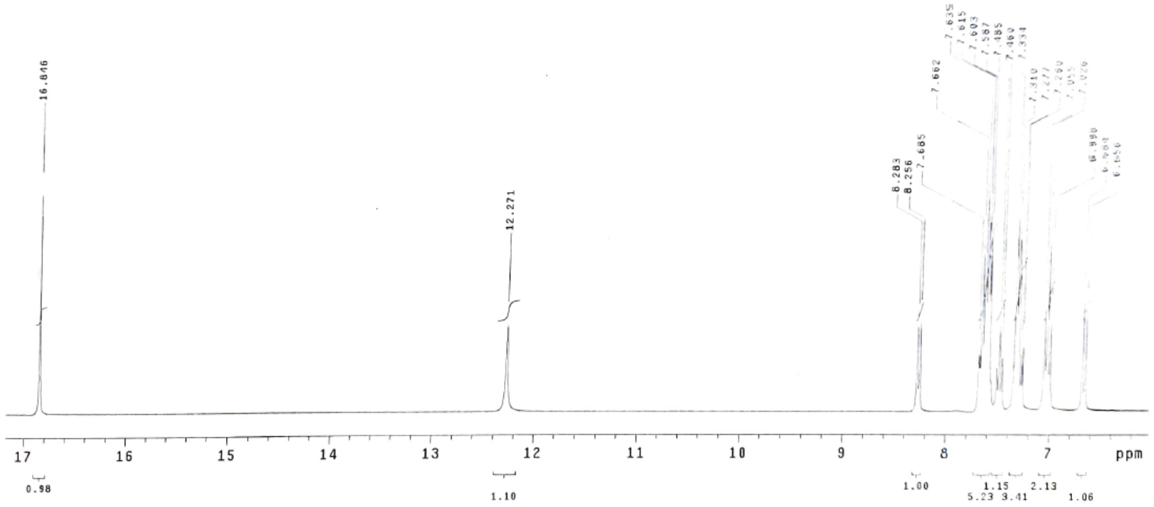

^{1}H NMR of compound 3t

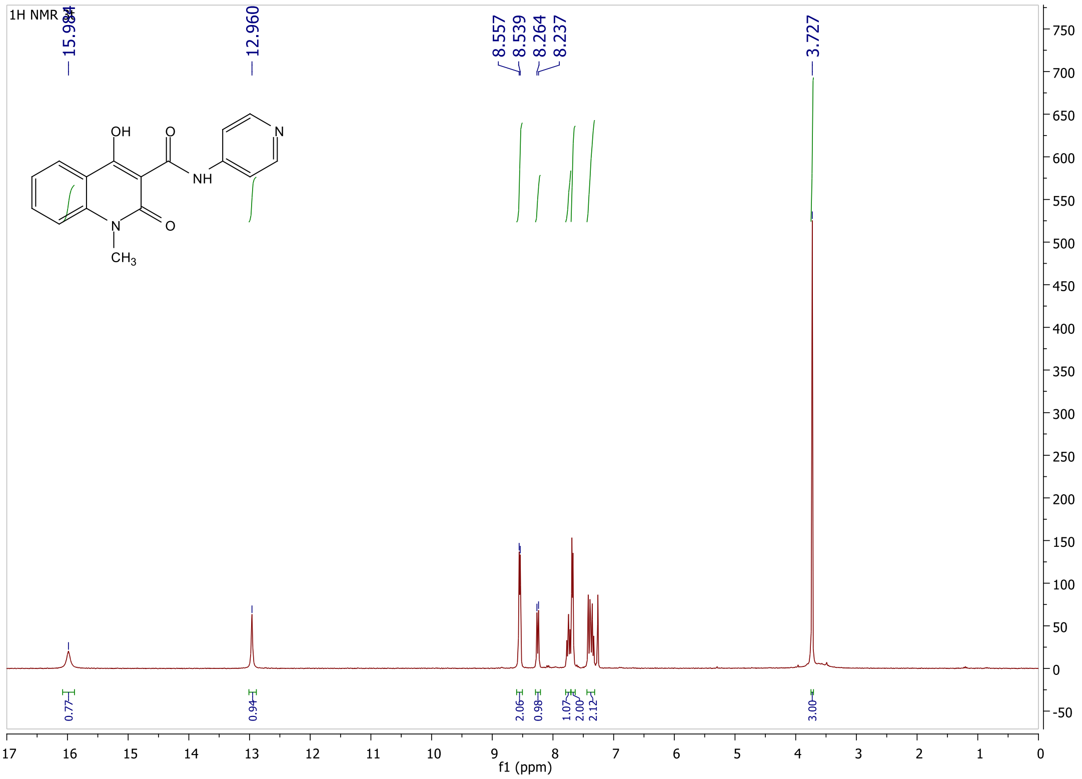

^{1}H NMR of compound 3u

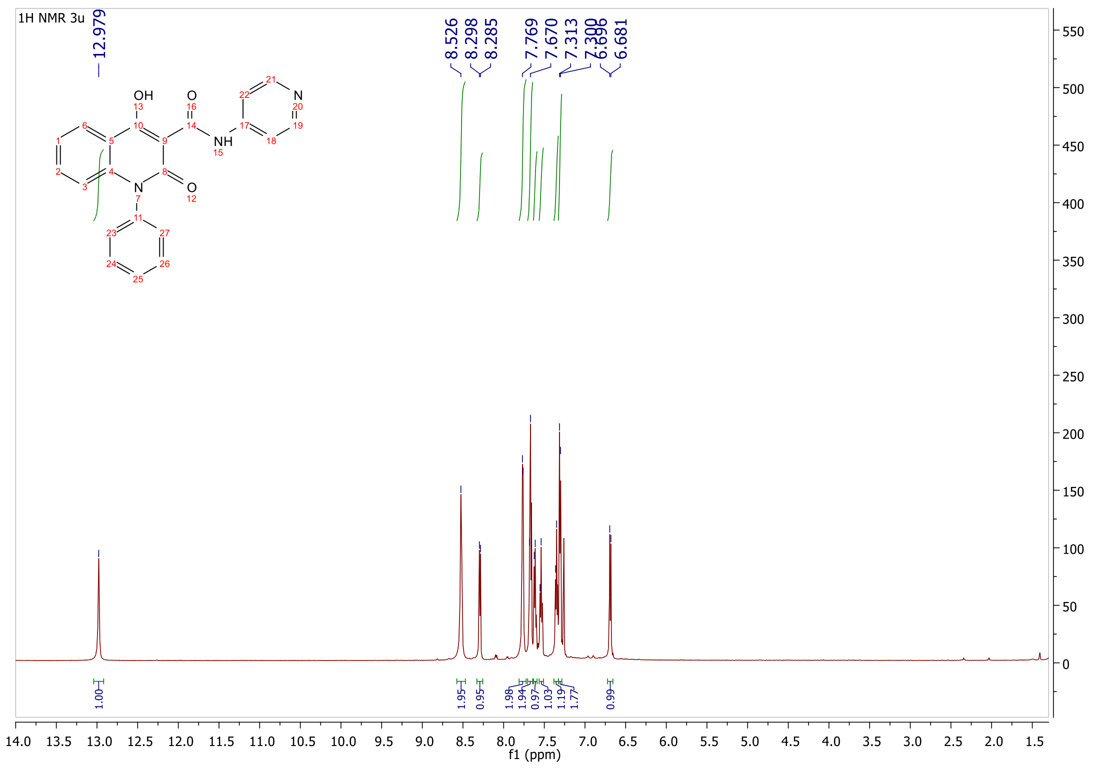

^{1}H NMR of compound 7

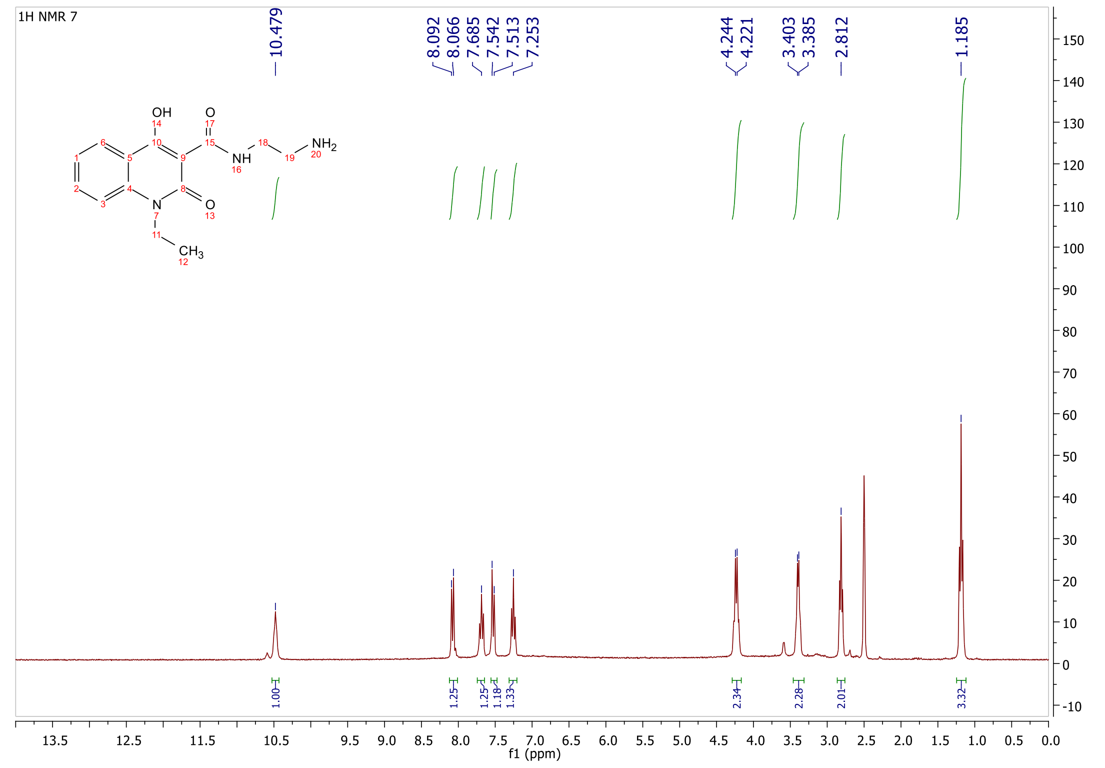

^{1}H NMR of compound 11a

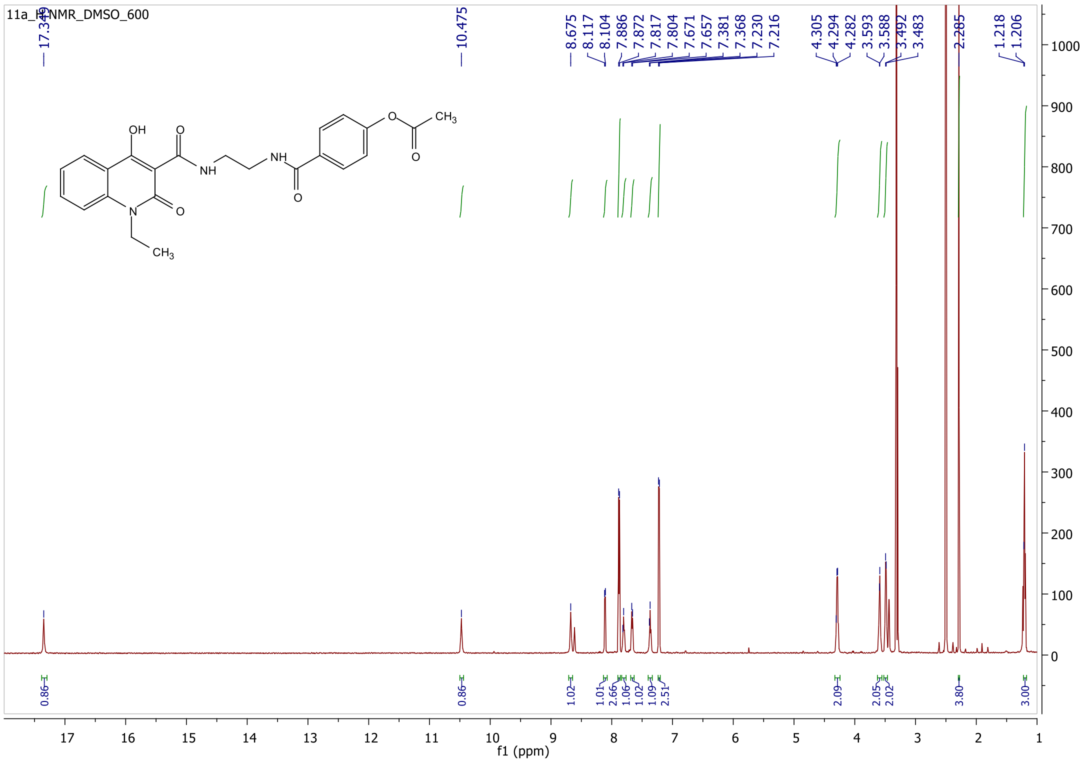

^{1}H NMR of compound 11b

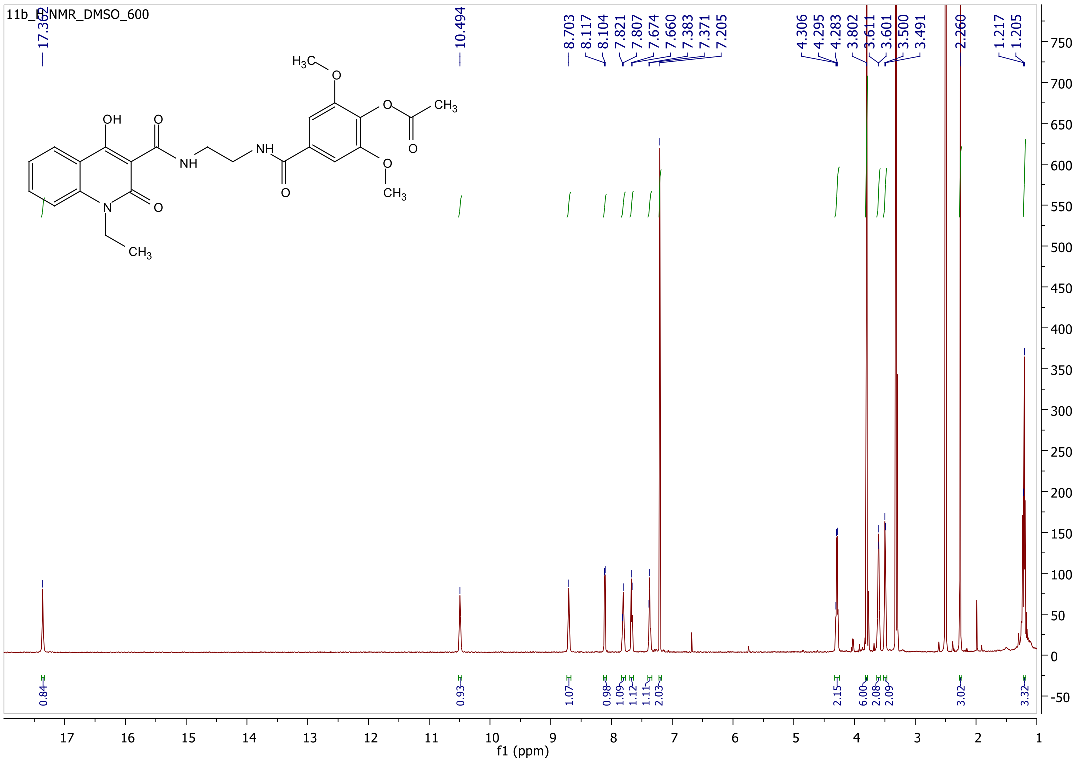

^{1}H NMR of compound 11c

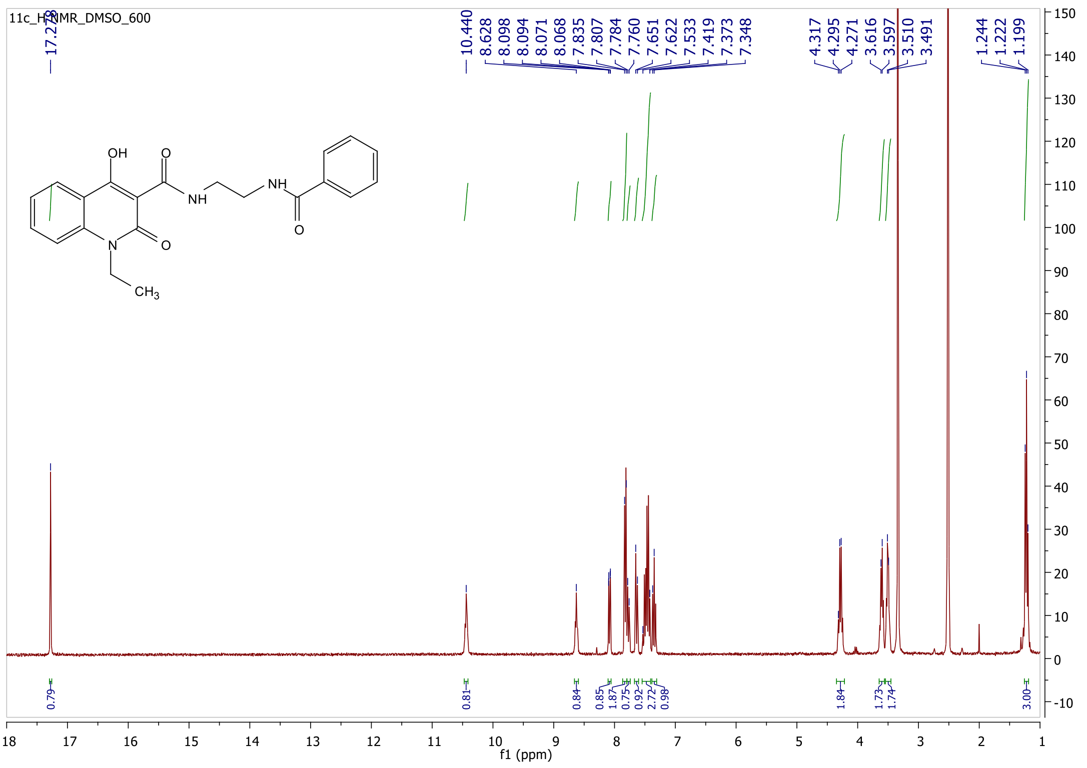

^{1}H NMR of compound 11d

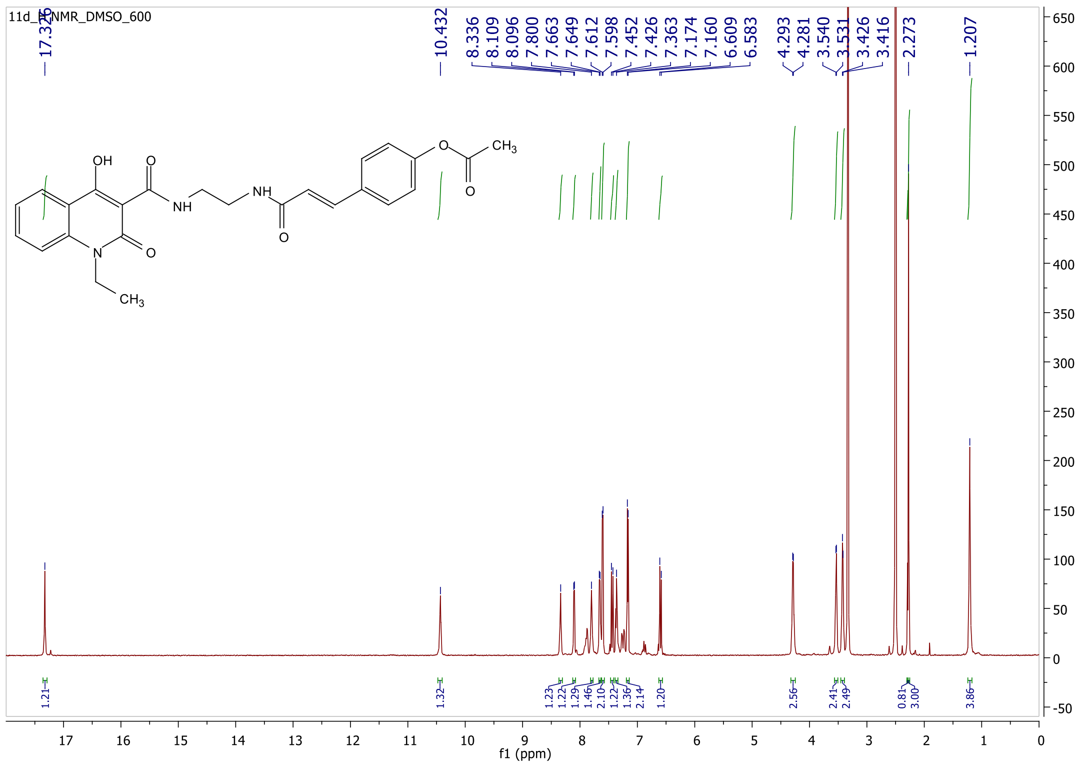

^{1}H NMR of compound 11e

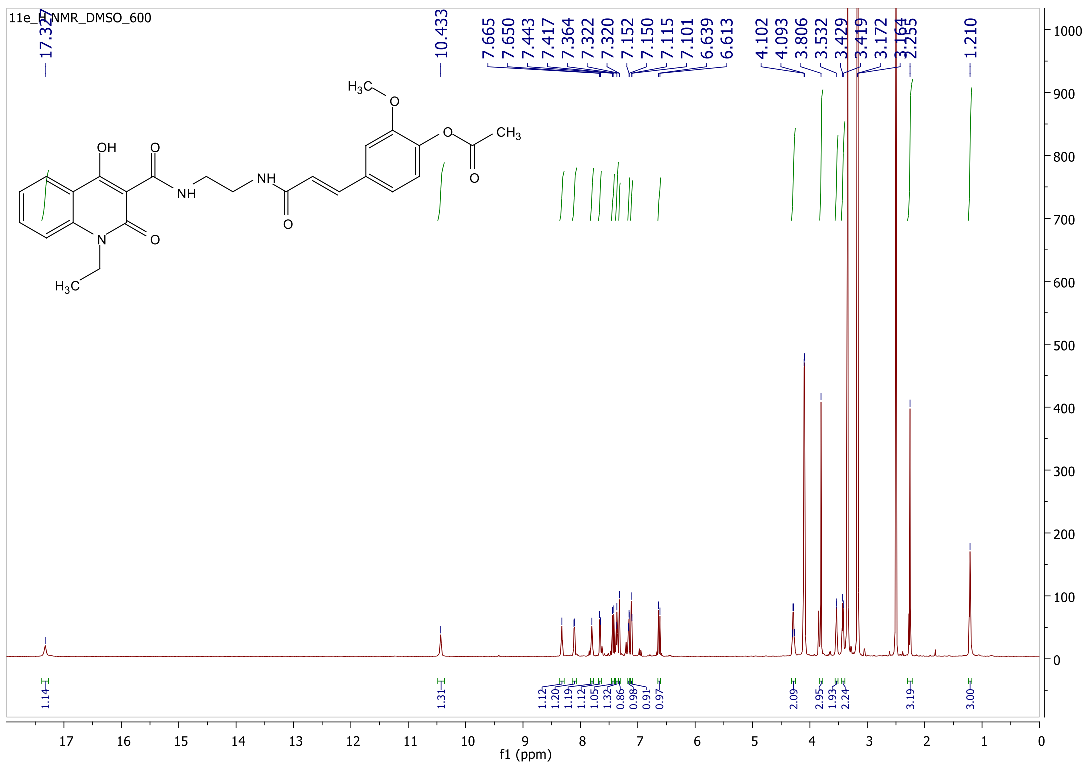

^{1}H NMR of compound 11f

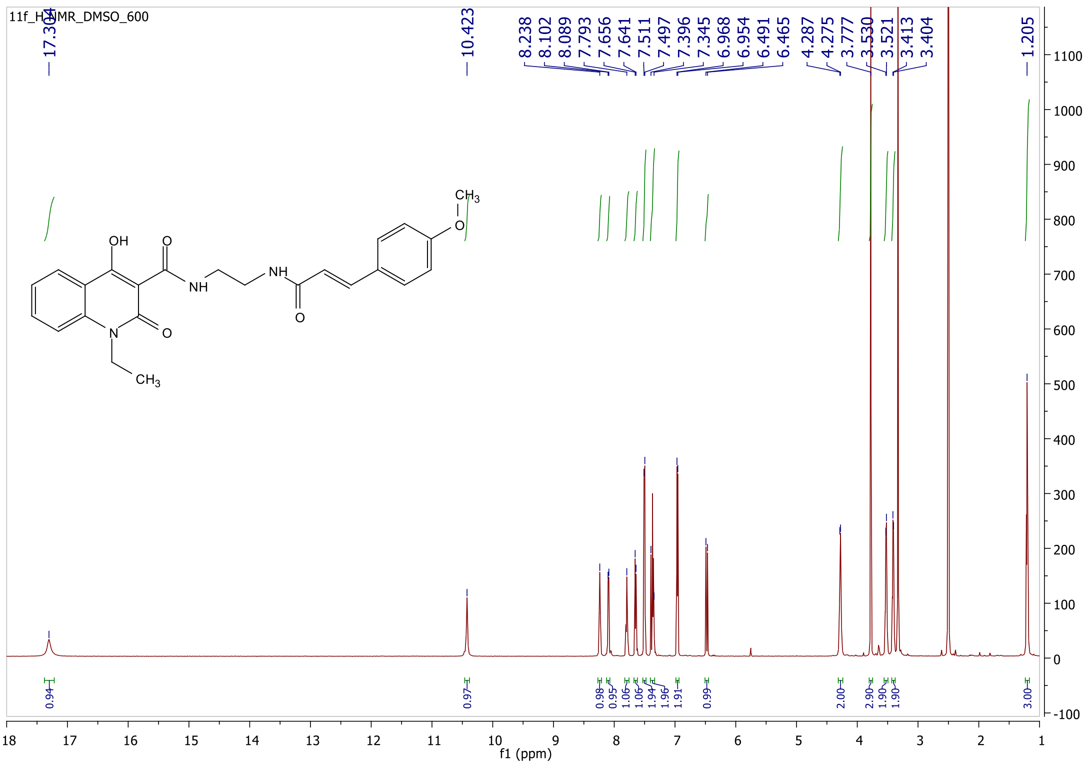

^{1}H NMR of compound 11g

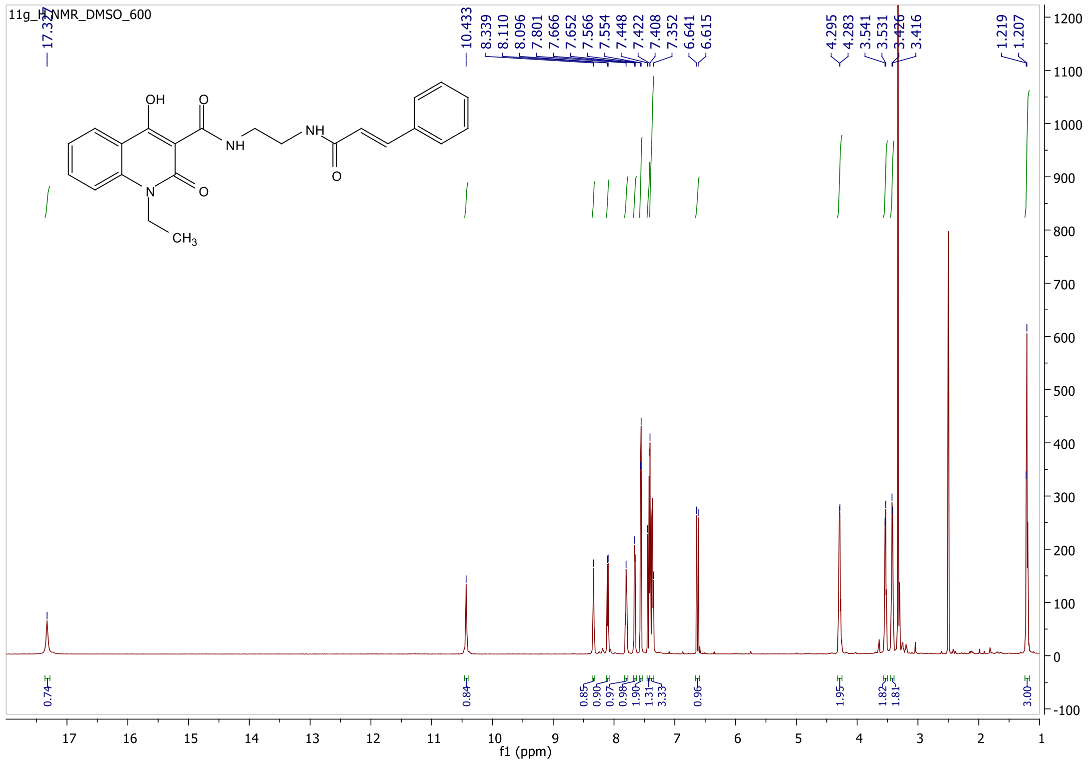

^{1}H NMR of compound 16a

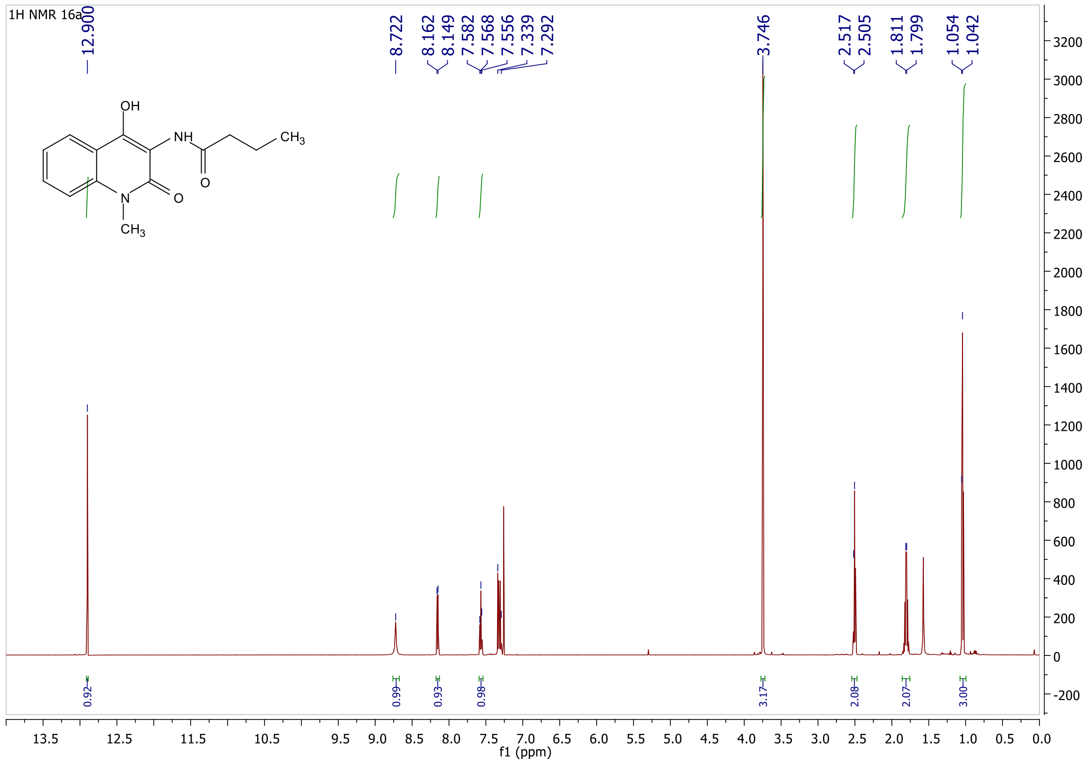

^{1}H NMR of compound 16b

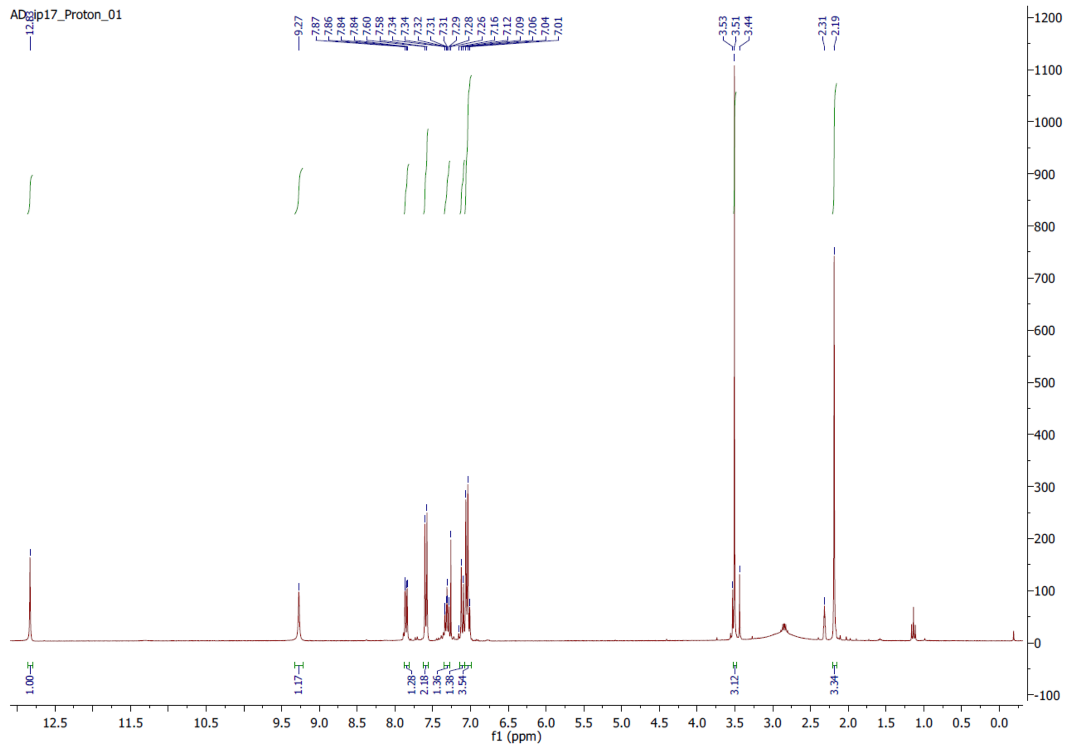

^{1}H NMR of compound 16c

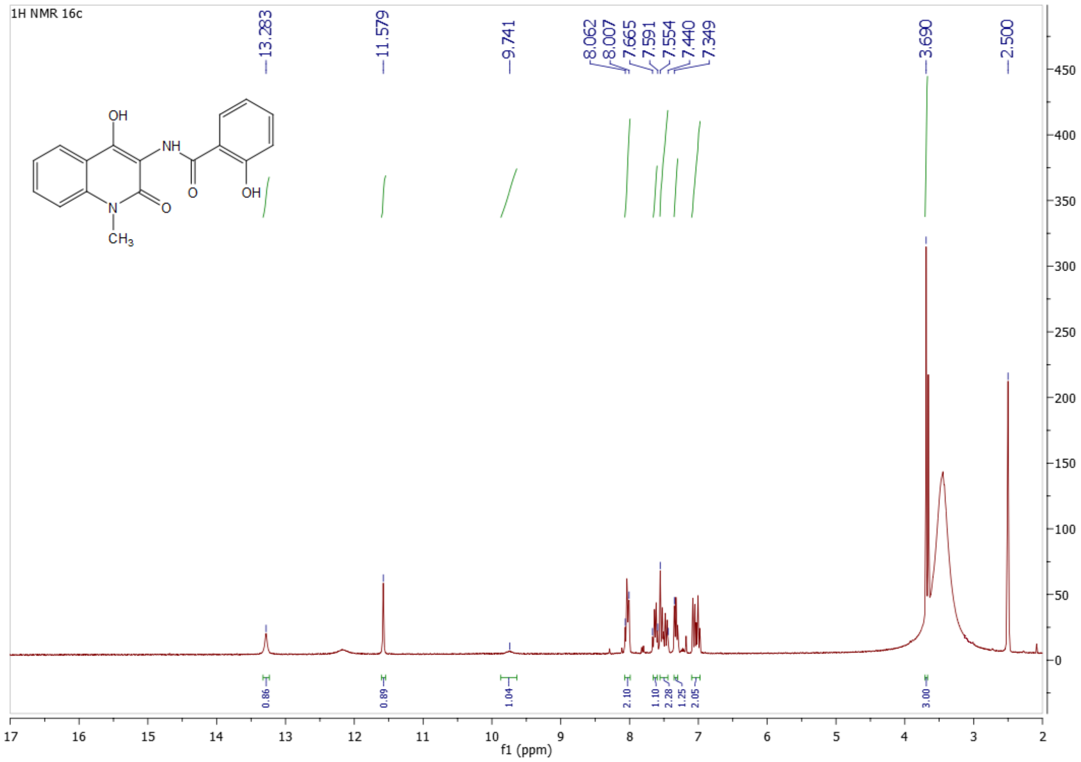

^{13}C NMR of compound 3a

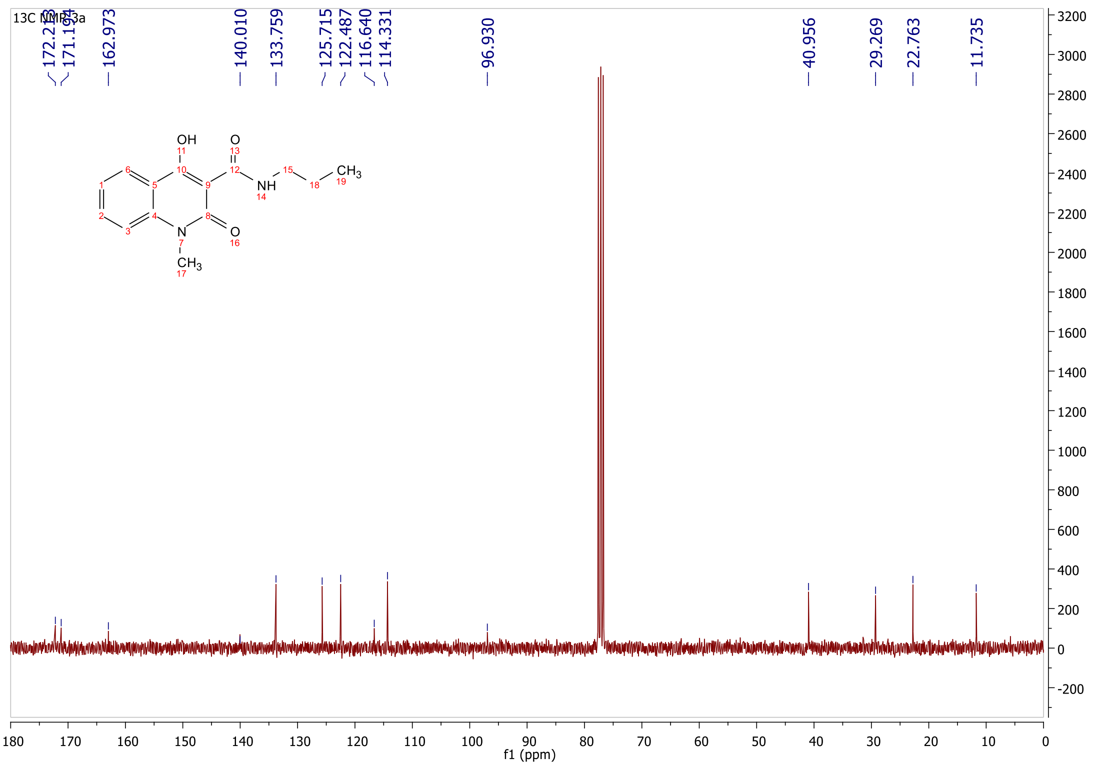

^{13}C NMR of compound 3b

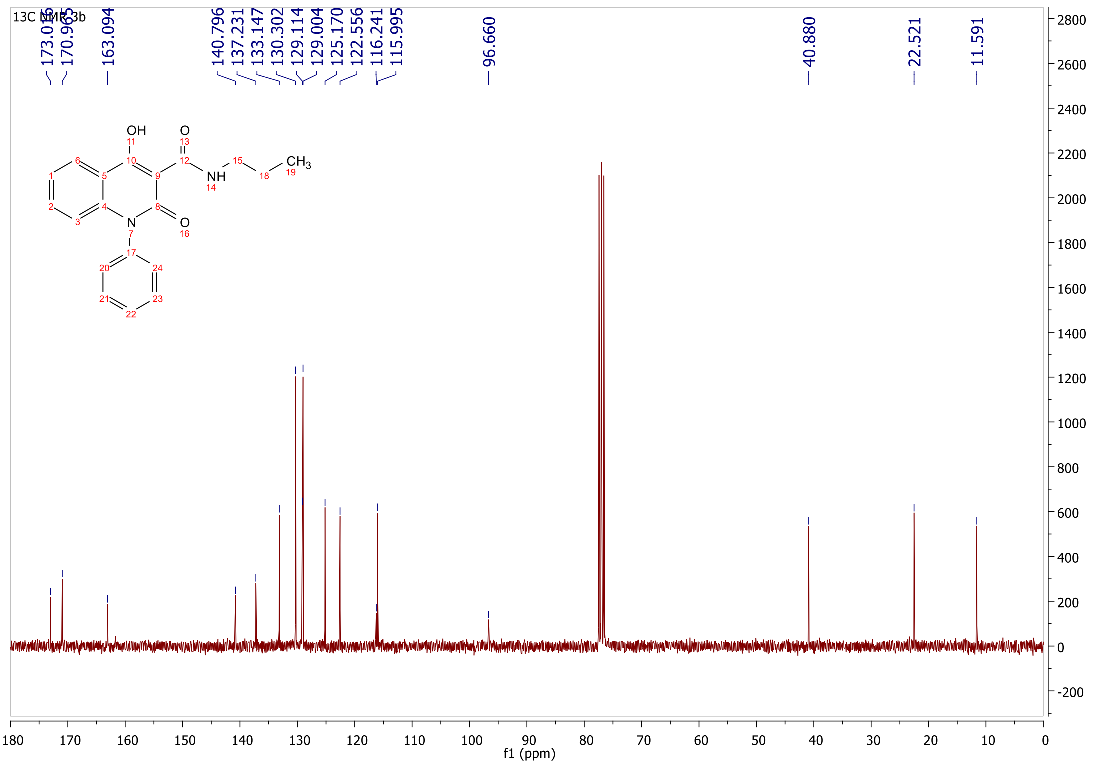

^{13}C NMR of compound 3c

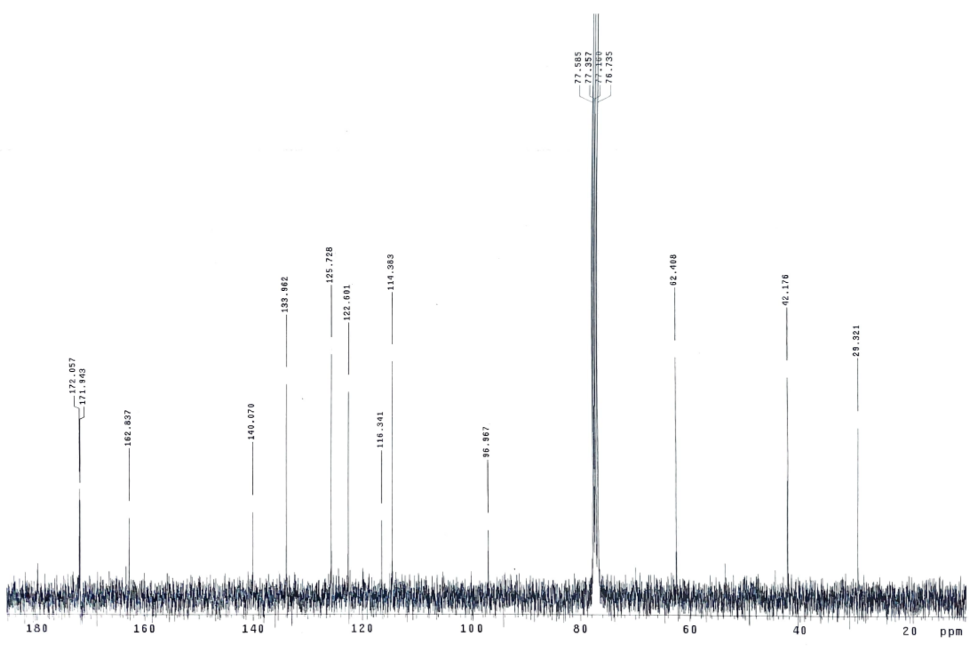

^{13}C NMR of compound 3d

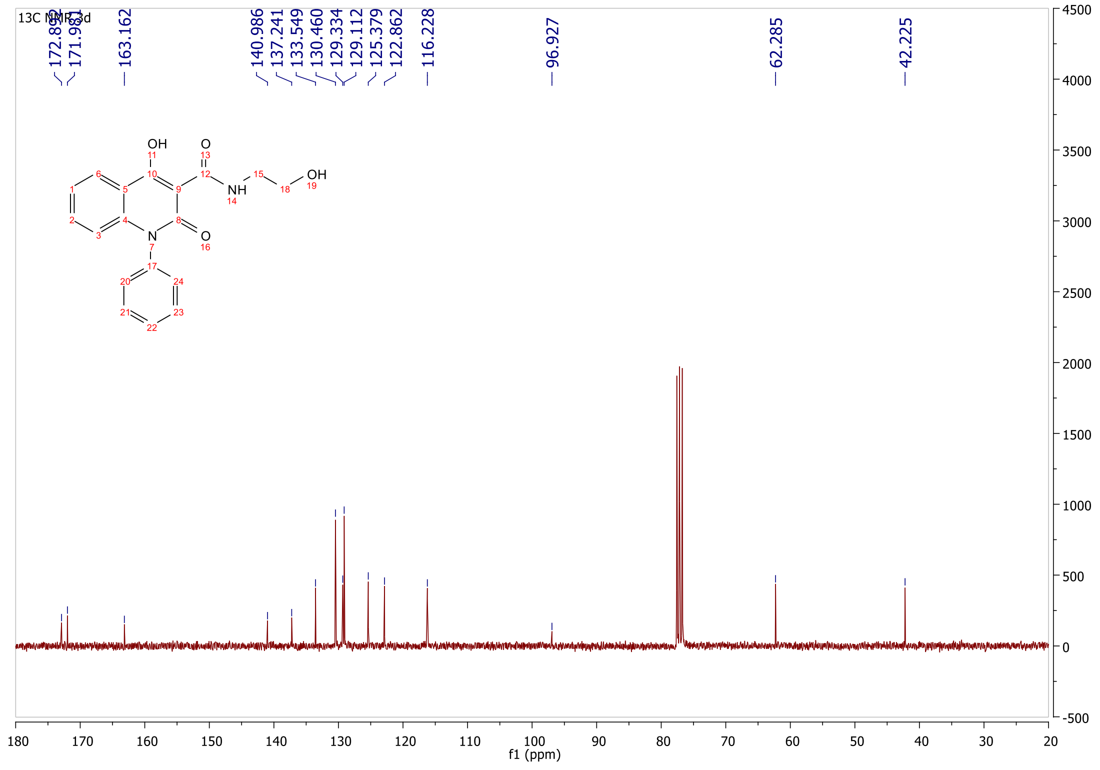

^{13}C NMR of compound 3e

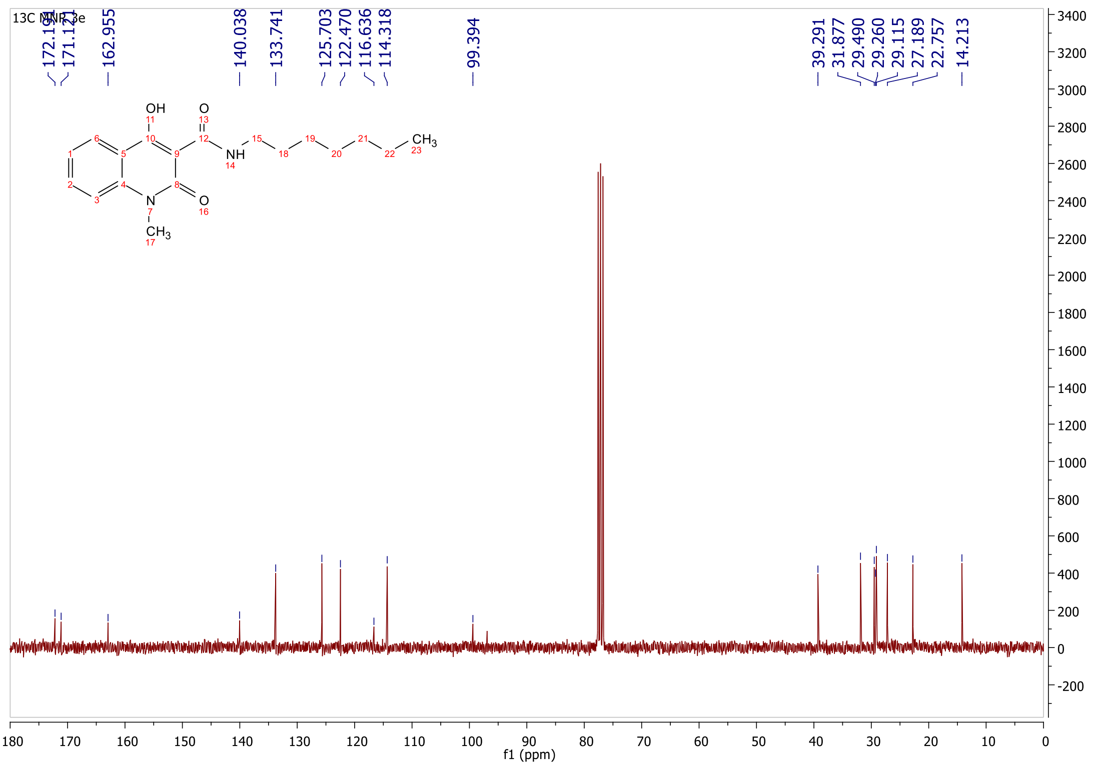

^{13}C NMR of compound 3f

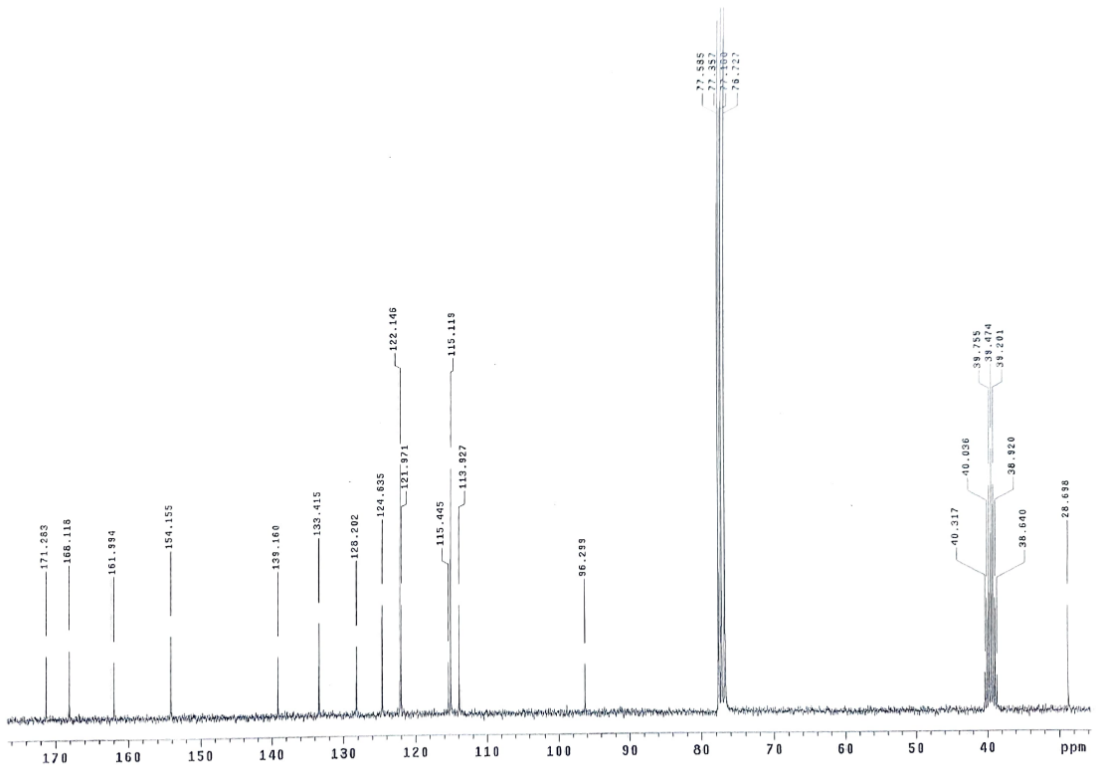

^{13}C NMR of compound 3g

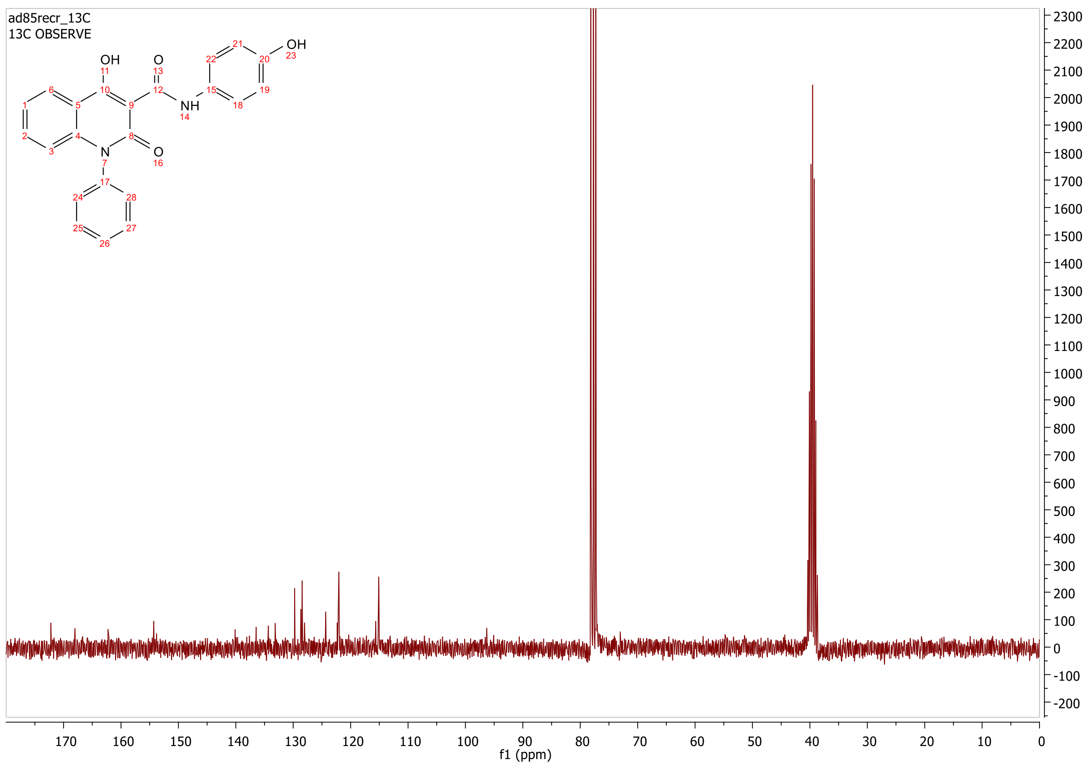

^{13}C NMR of compound 3h

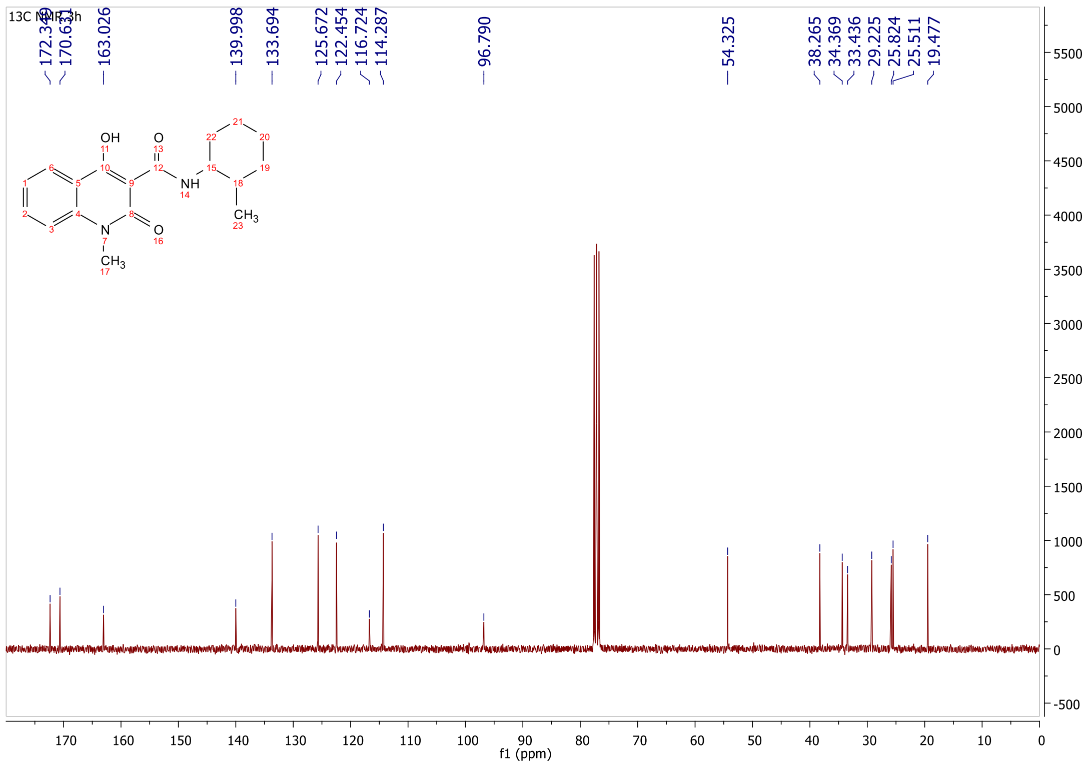

¹³C NMR of compound 3i

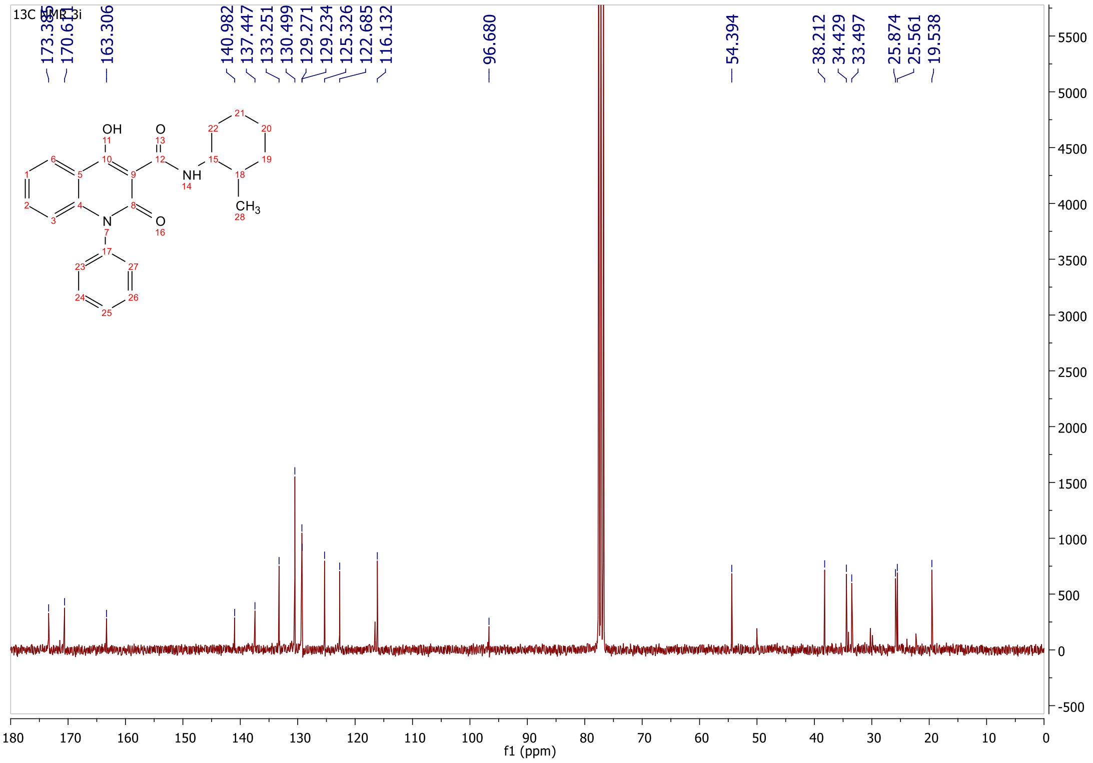

¹³C NMR of compound 3j

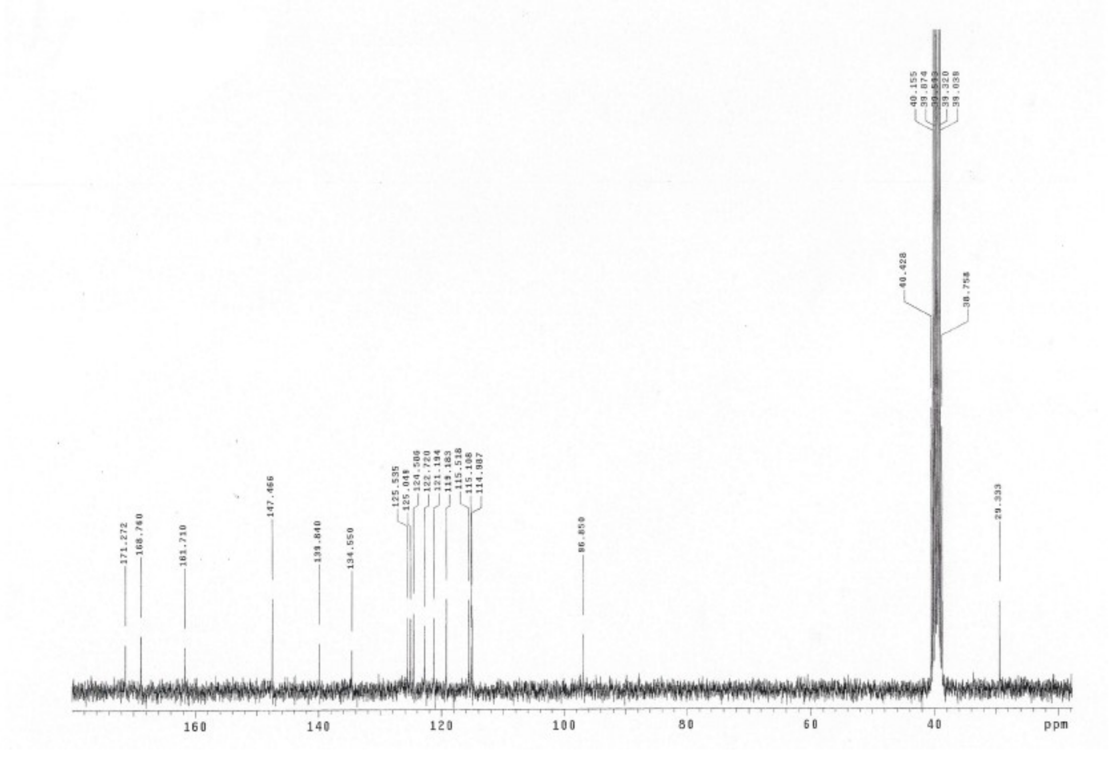

^{13}C NMR of compound 3k

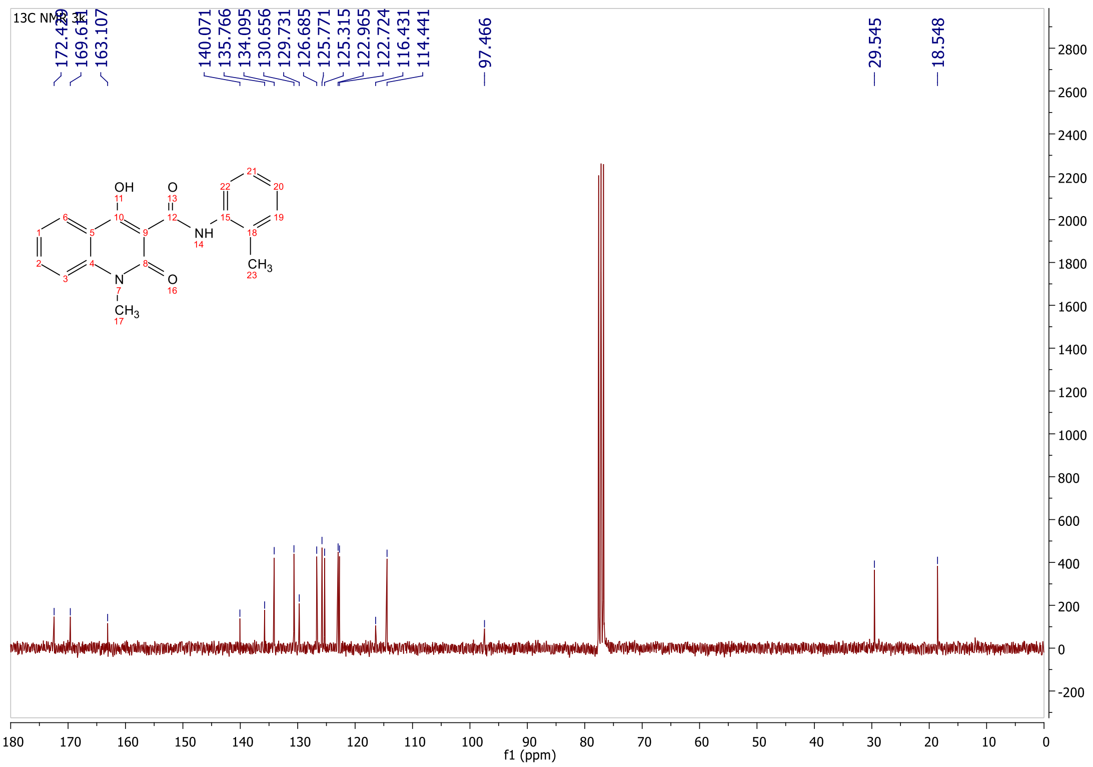

^{13}C NMR of compound 3l

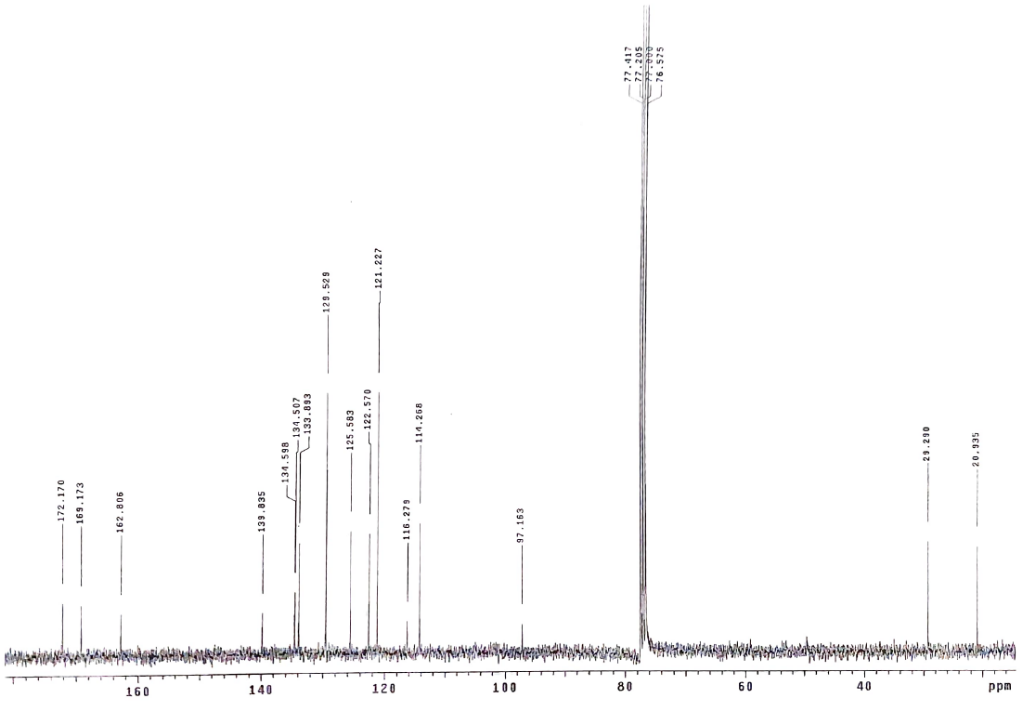

^{13}C NMR of compound 3m

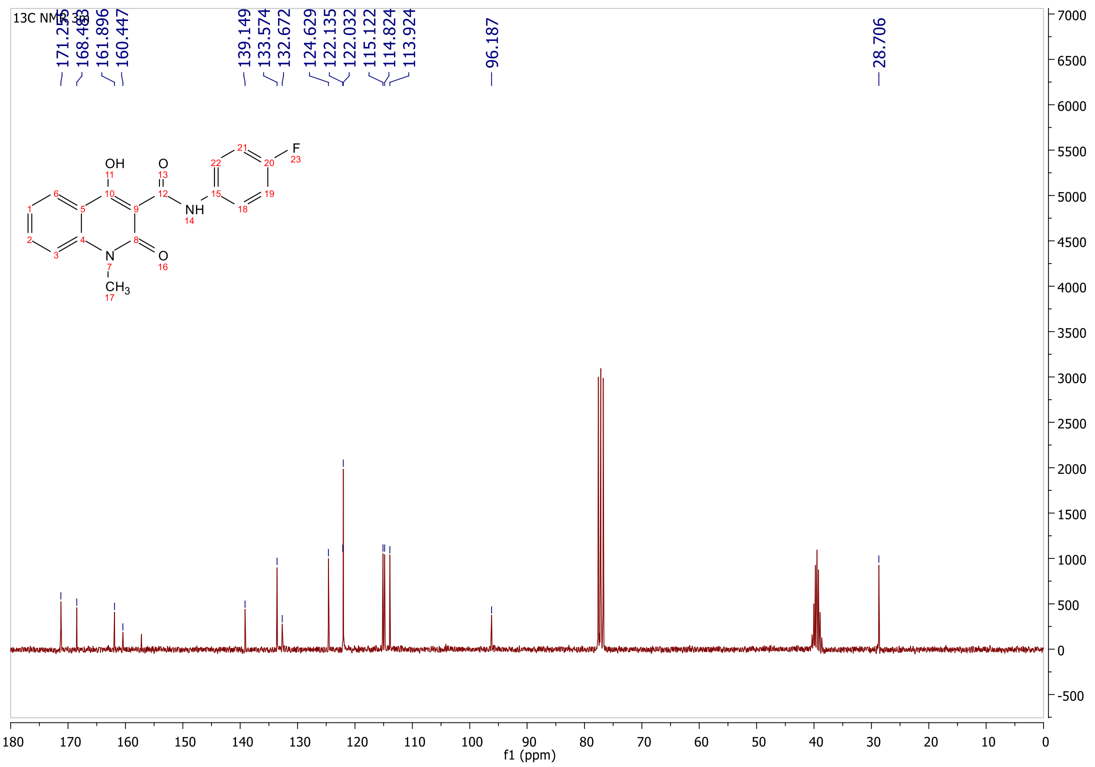

^{13}C NMR of compound 3n

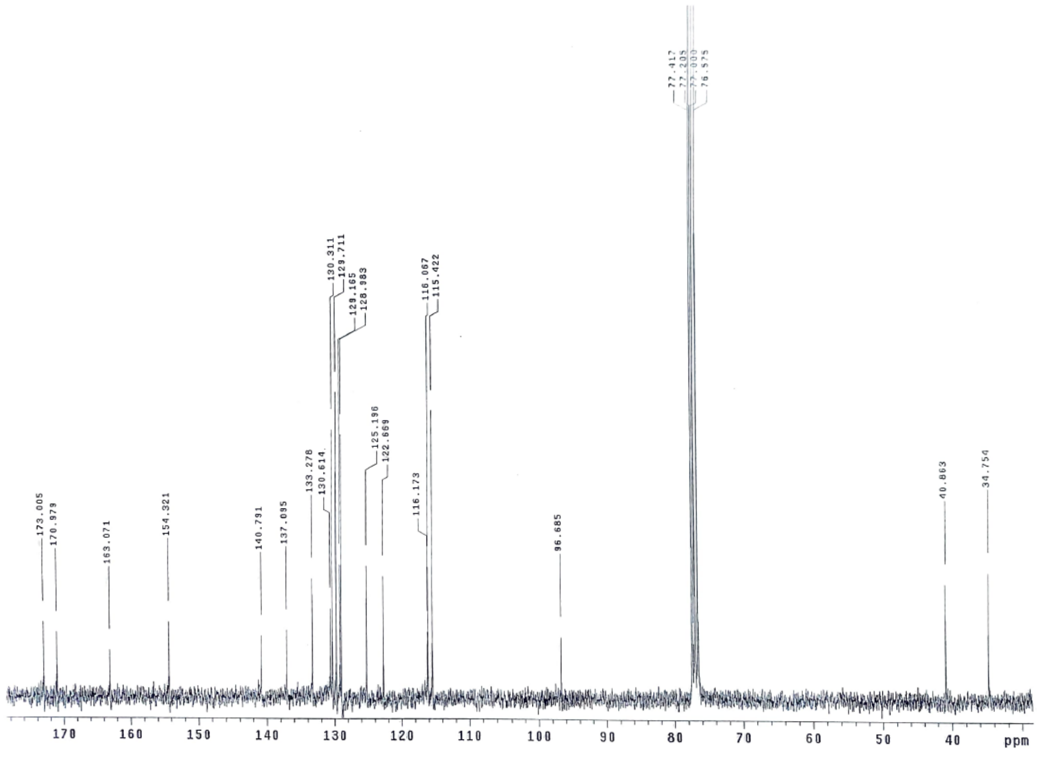

^{13}C NMR of compound 3o

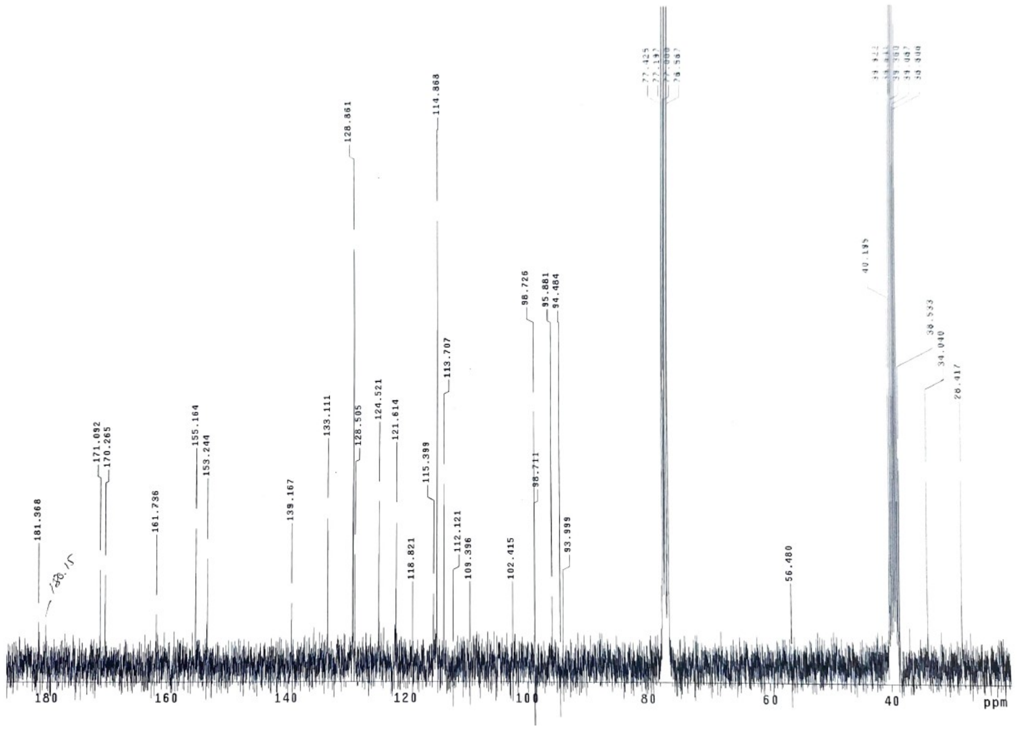

^{13}C NMR of compound 3p

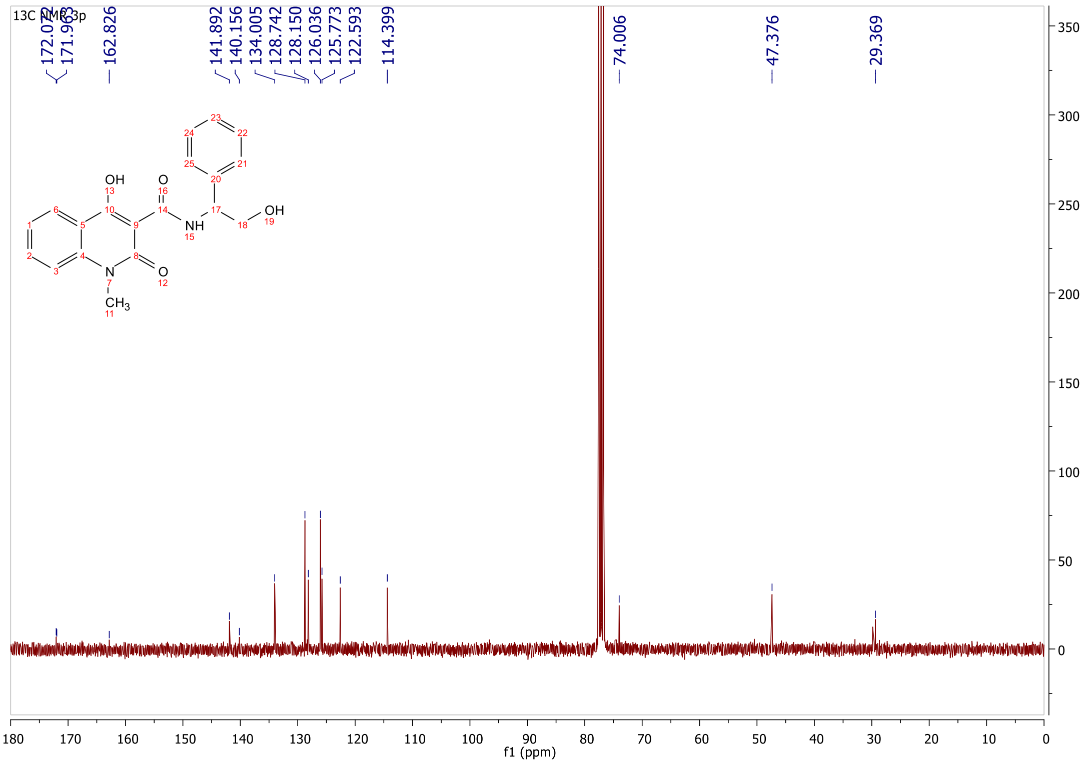

^{13}C NMR of compound 3q

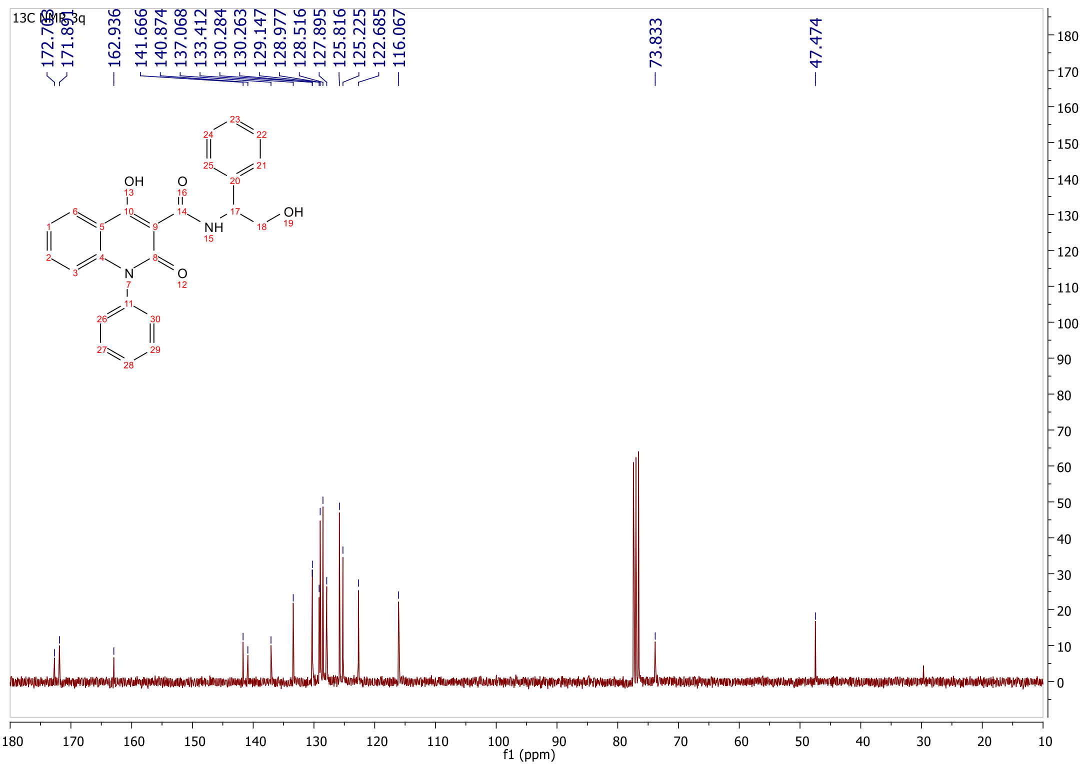

^{13}C NMR of compound 3r

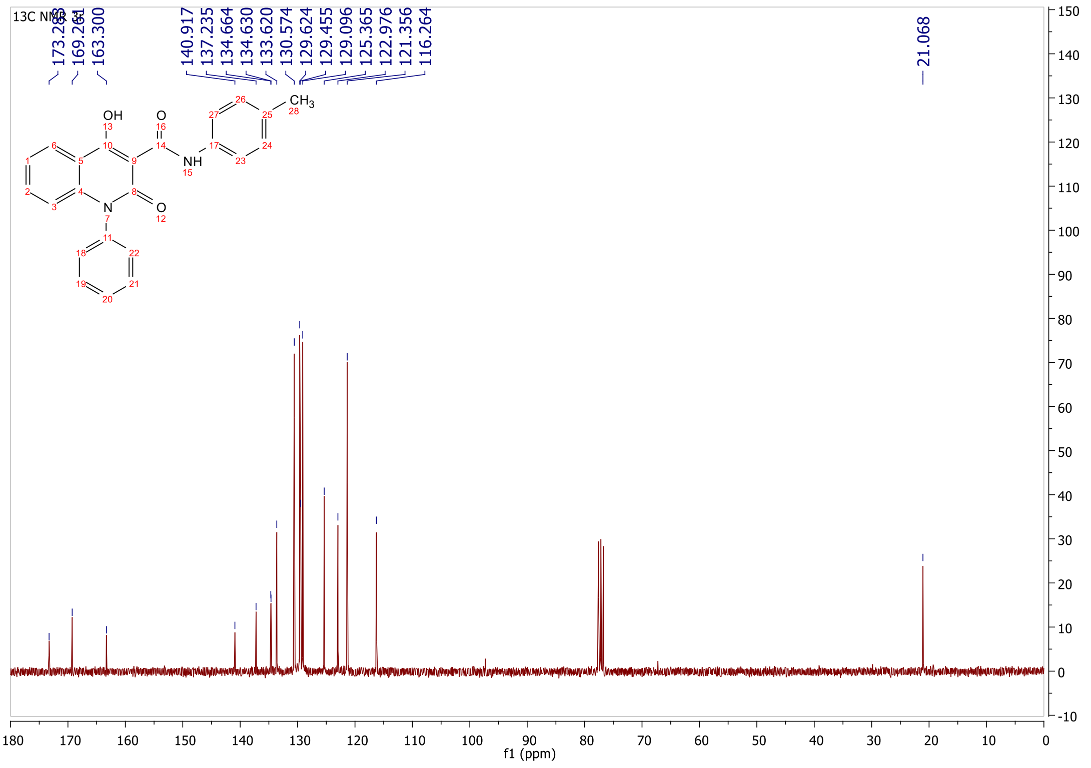

^{13}C NMR of compound 3s

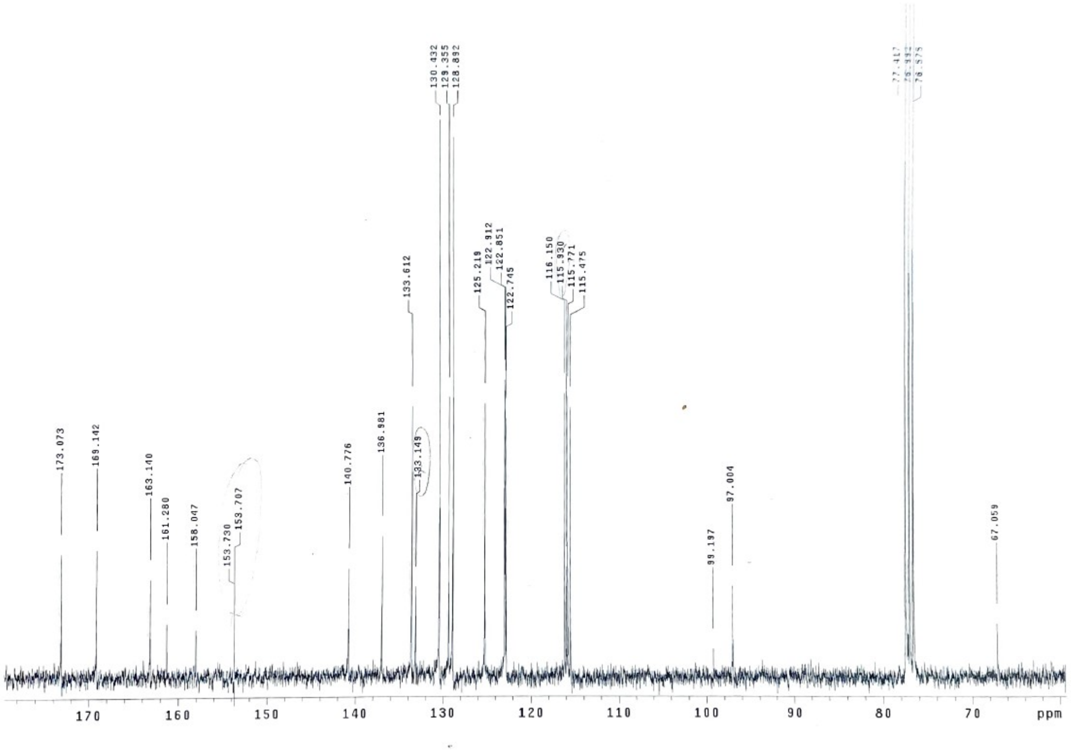

^{13}C NMR of compound 3u

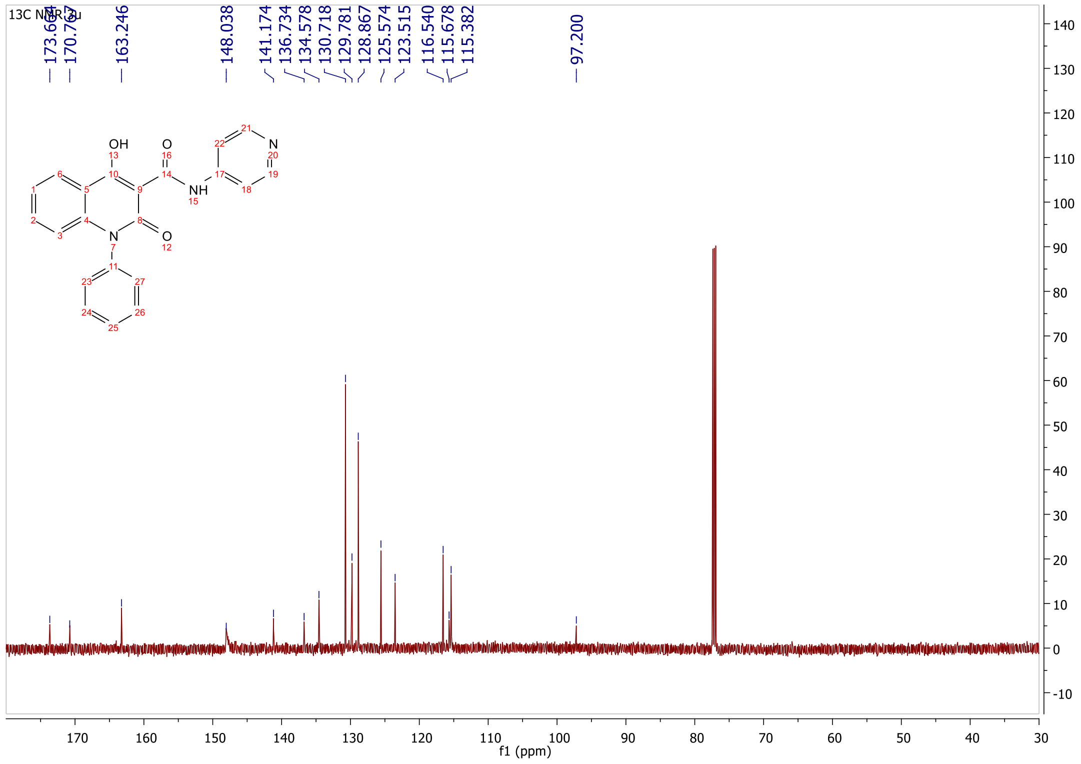

^{13}C NMR of compound 7

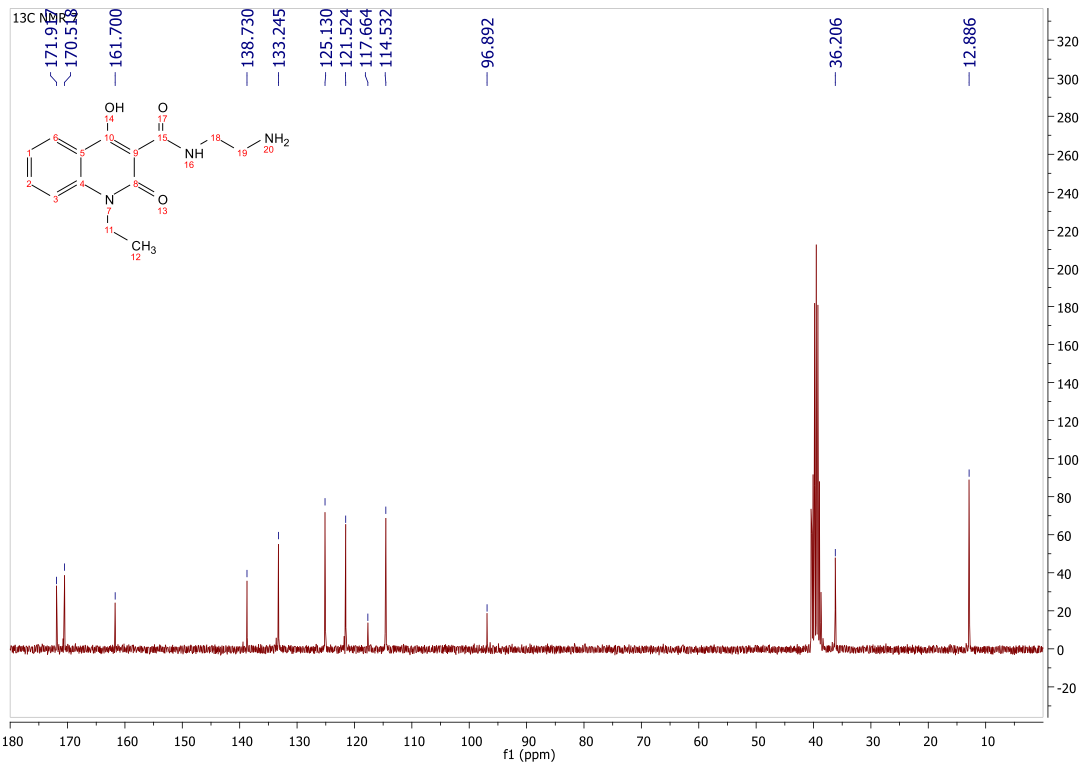

^{13}C NMR of compound 11a

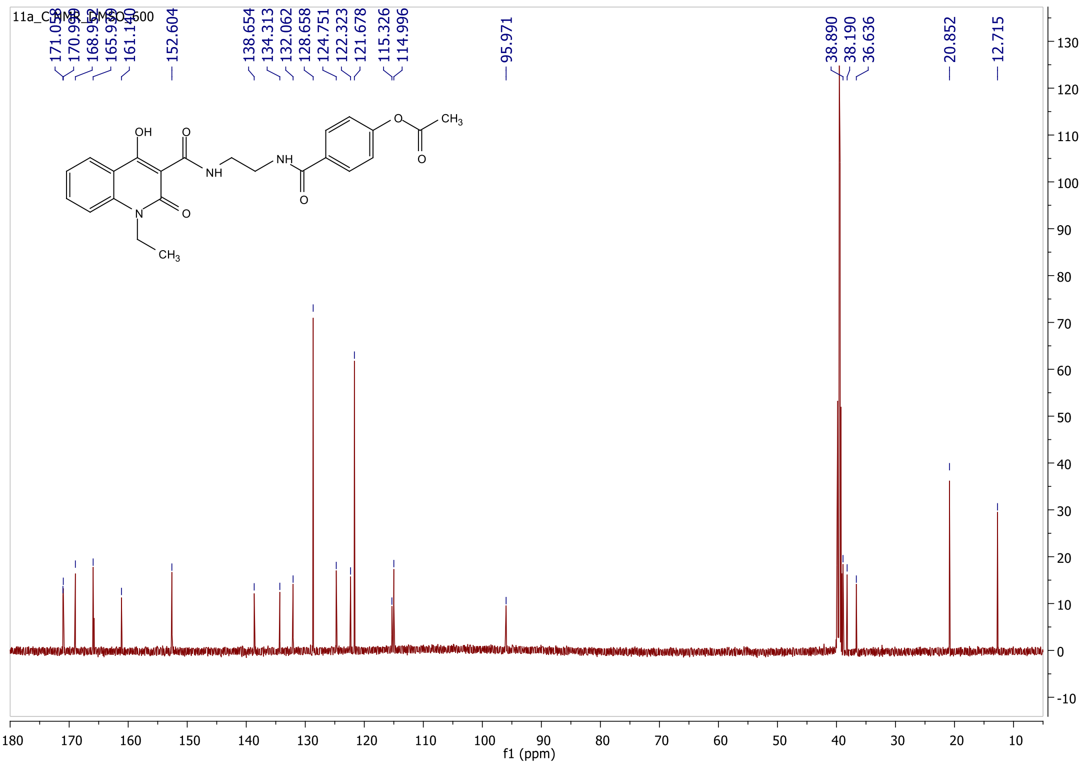

^{13}C NMR of compound 11b

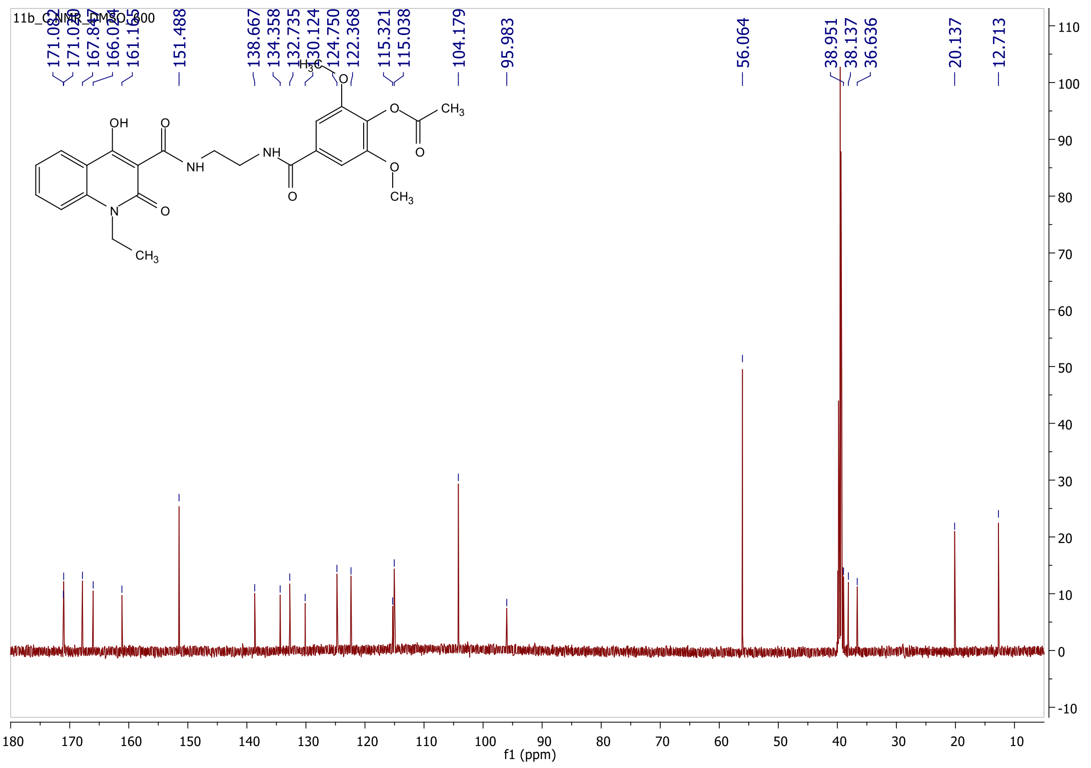

^{13}C NMR of compound 11c

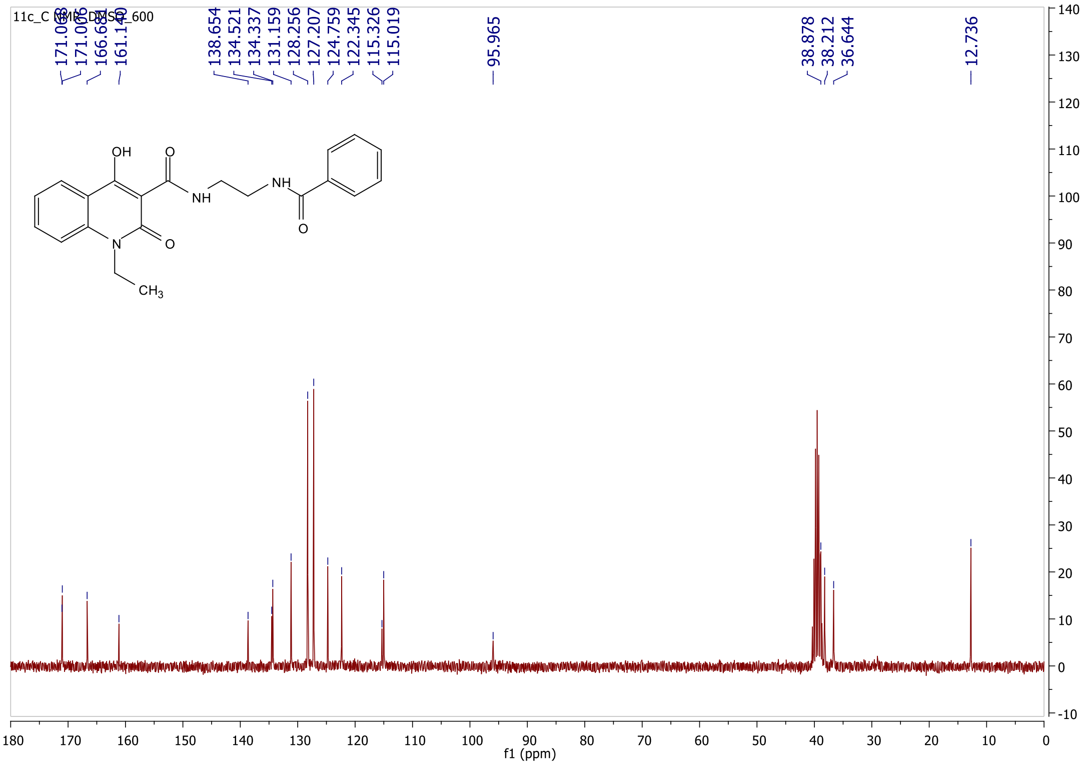

^{13}C NMR of compound 11d

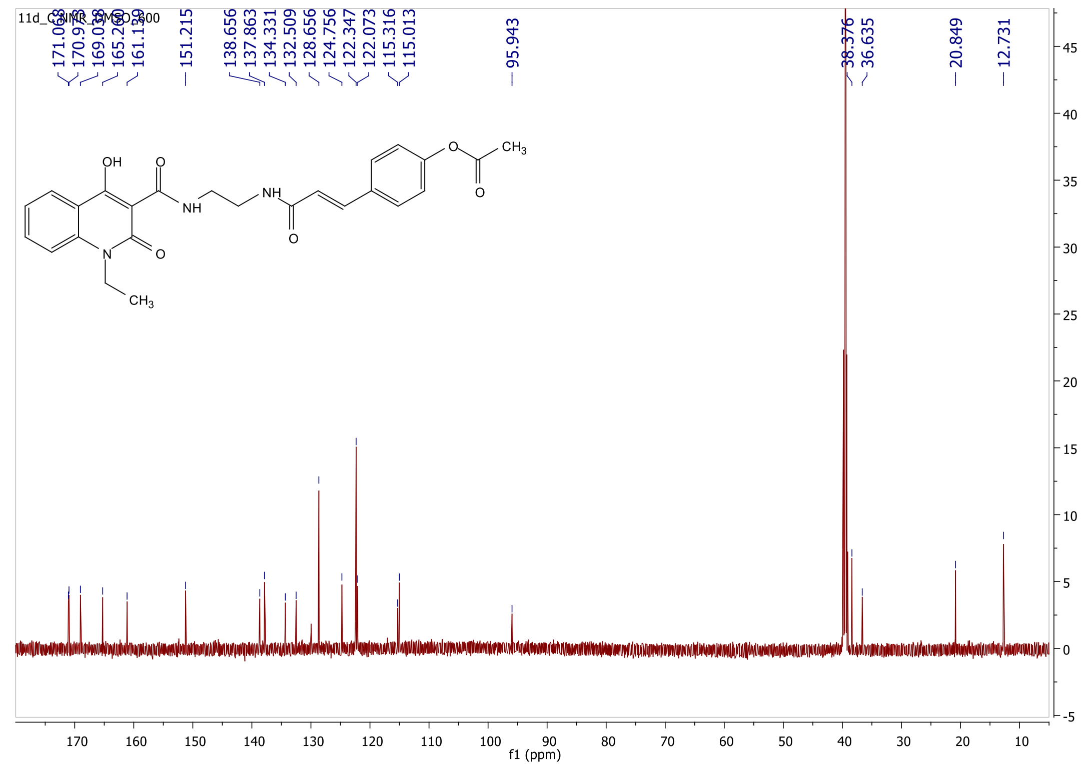

^{13}C NMR of compound 11e

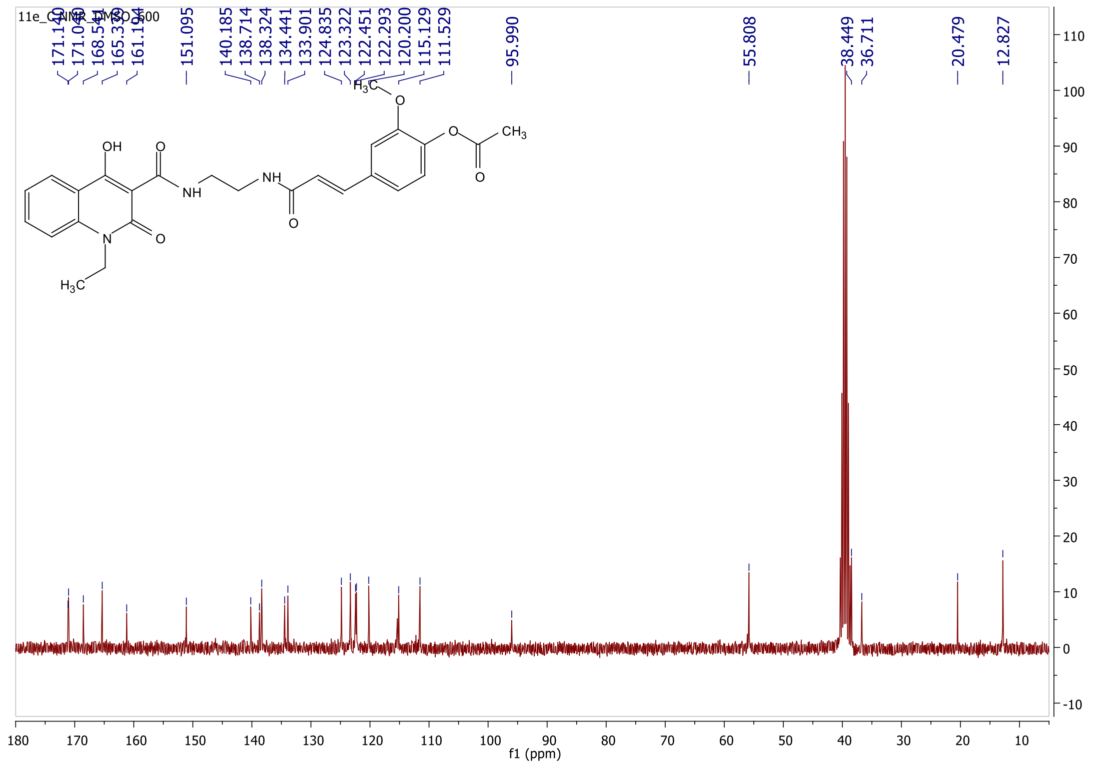

^{13}C NMR of compound 11f

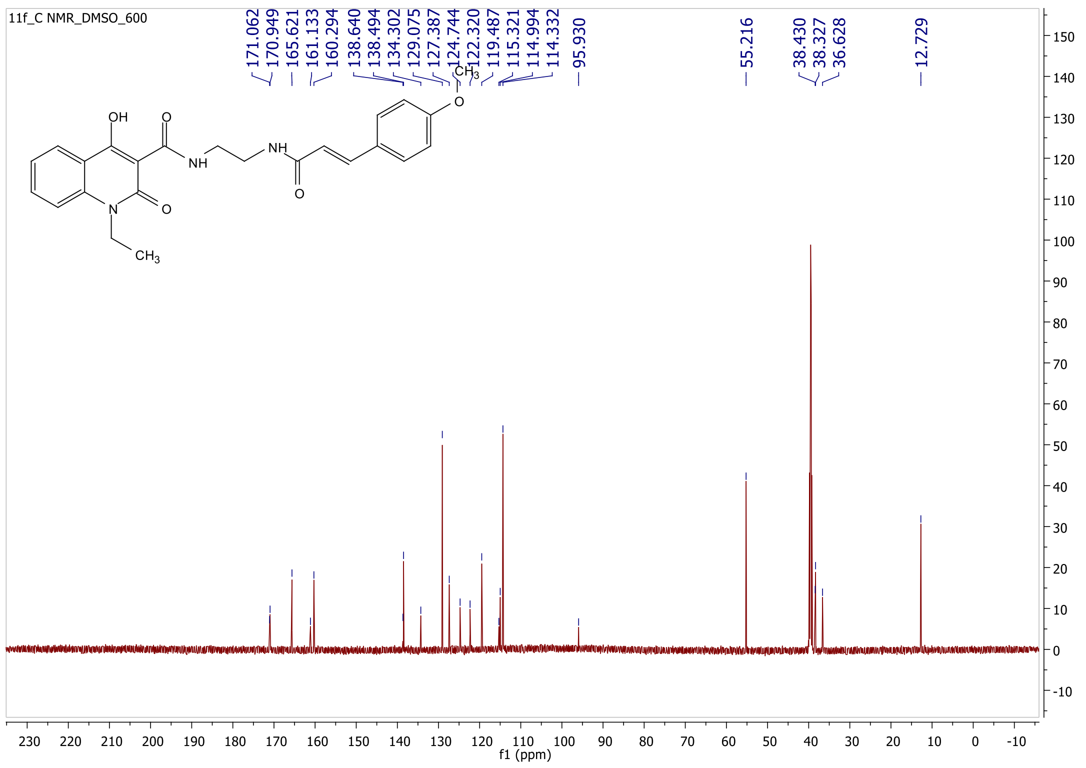

^{13}C NMR of compound 11g

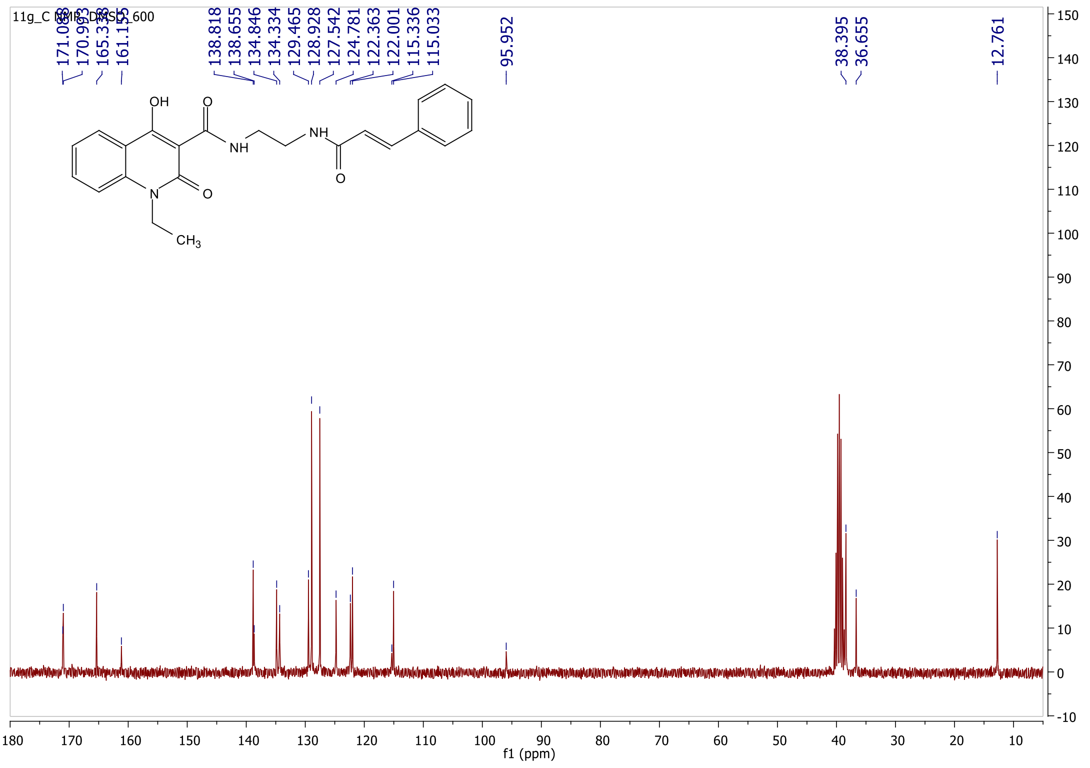

^{13}C NMR of compound 16a

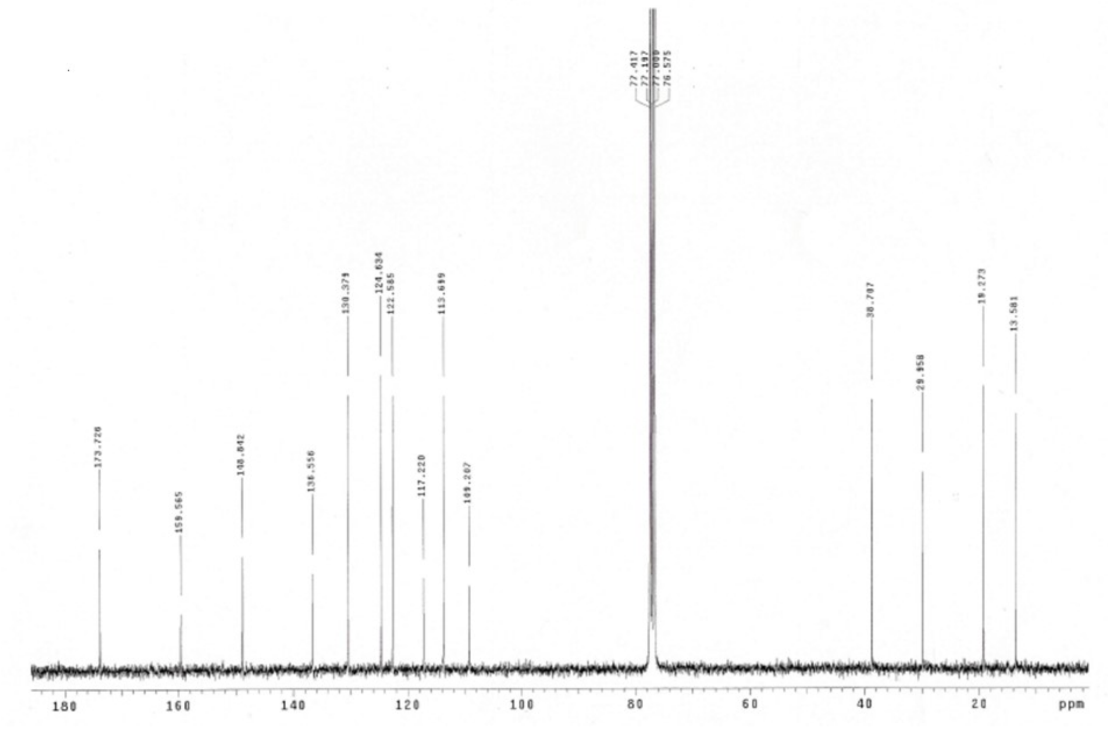

^{13}C NMR of compound 16b

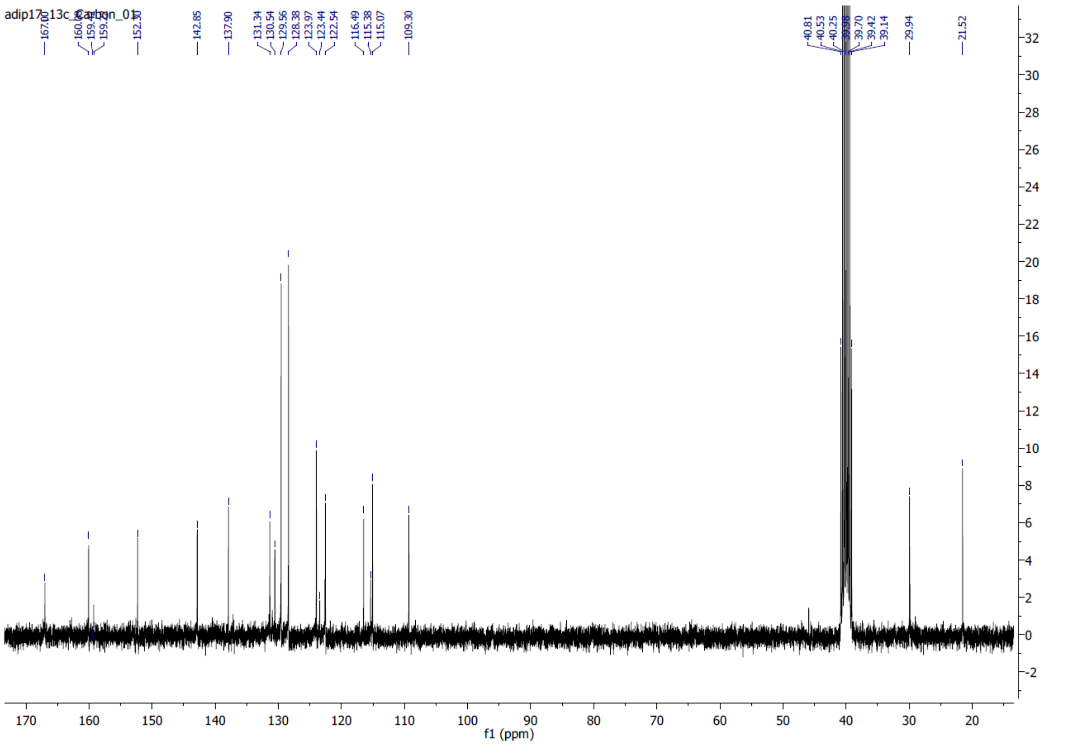

^{13}C NMR of compound 16c

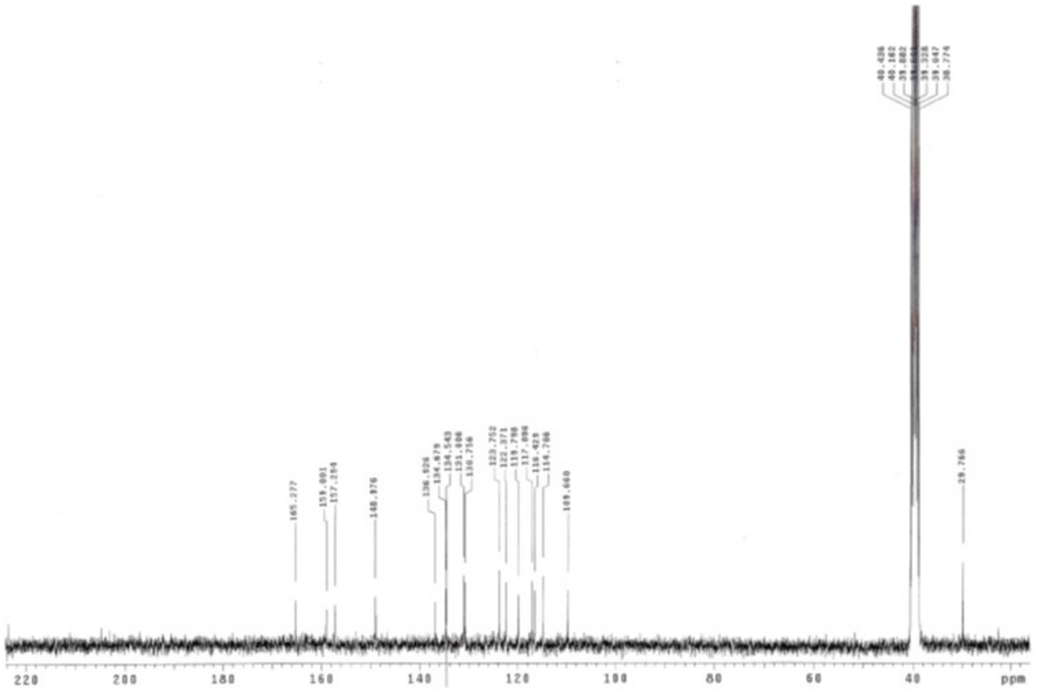

HRESIMS (pos) of compound 3a

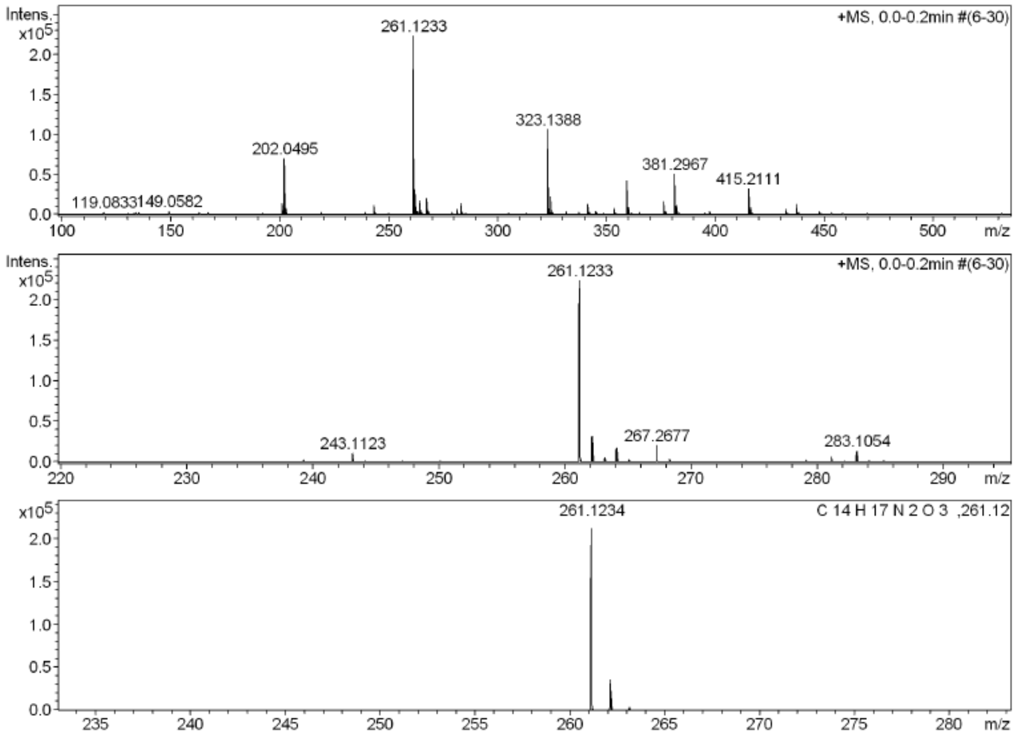

Meas. m/z	#	Formula	m/z	err [ppm]	Mean err [ppm]	rdb	N-Rule	e^- Conf
261.1233	1	C 14 H 17 N 2 O 3	261.1234	0.4	0.5	7.5	ok	even

HRESIMS (pos) of compound 3b

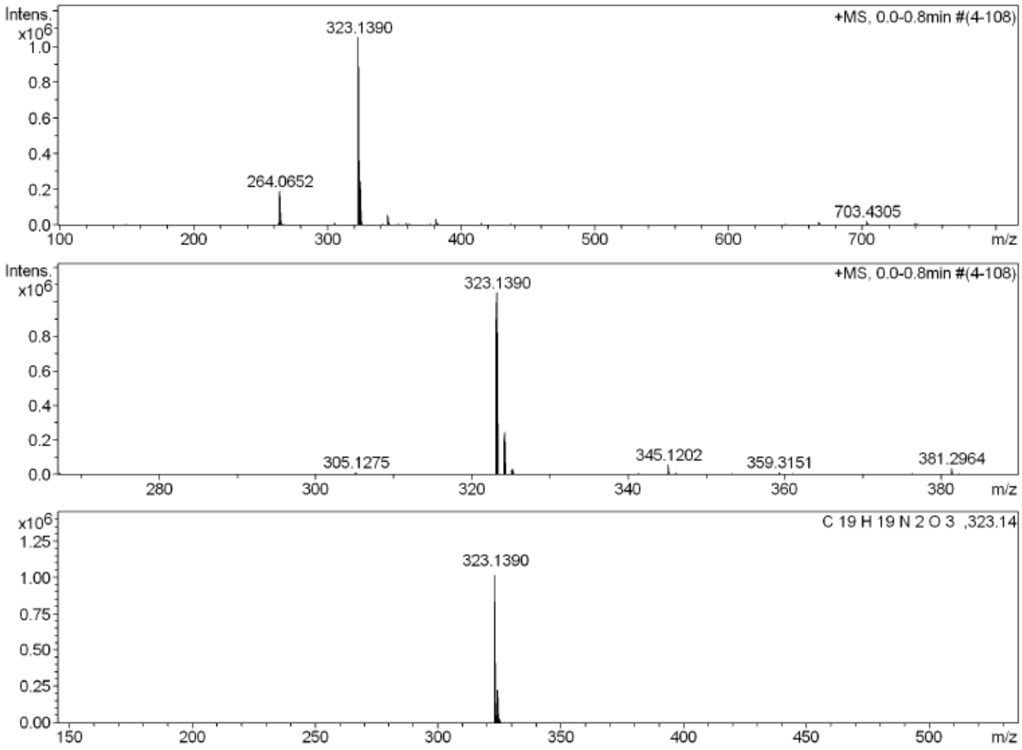

Meas. m/z	#	Formula	m/z	err [ppm]	Mean err [ppm]	rdb	N-Rule	e^- Conf
323.1390	1	C 19 H 19 N 2 O 3	323.1390	0.1	0.6	11.5	ok	even

HRESIMS (pos) of compound 3c

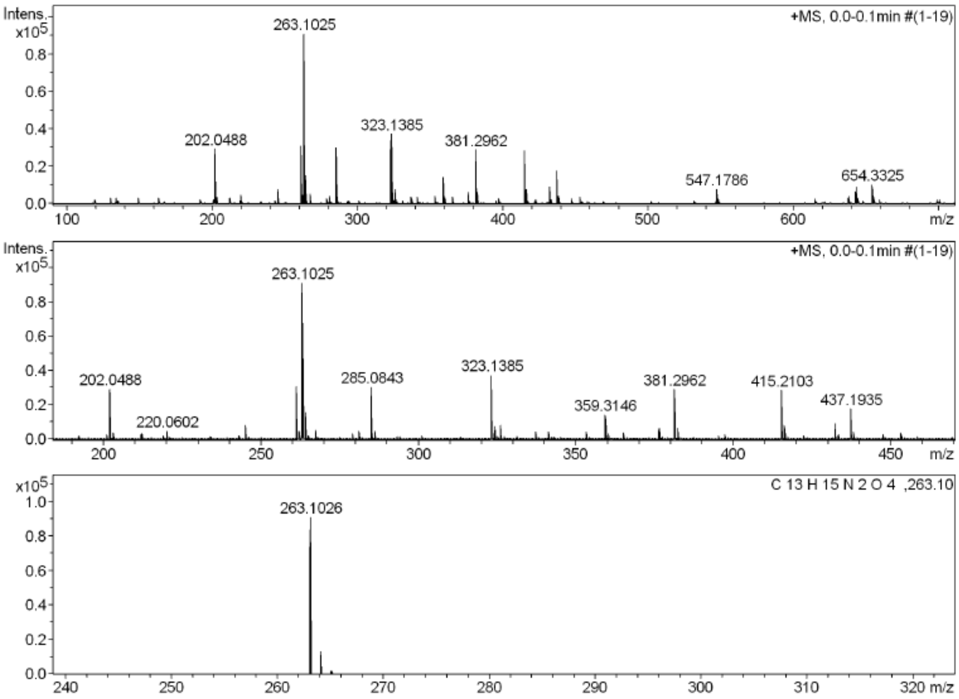

Meas. m/z	#	Formula	m/z	err [ppm]	Mean err [ppm]	rdb	N-Rule	e⁻ Conf
263.1025	1	C 13 H 15 N 2 O 4	263.1026	0.4	1.1	7.5	ok	even

HRESIMS (pos) of compound 3d

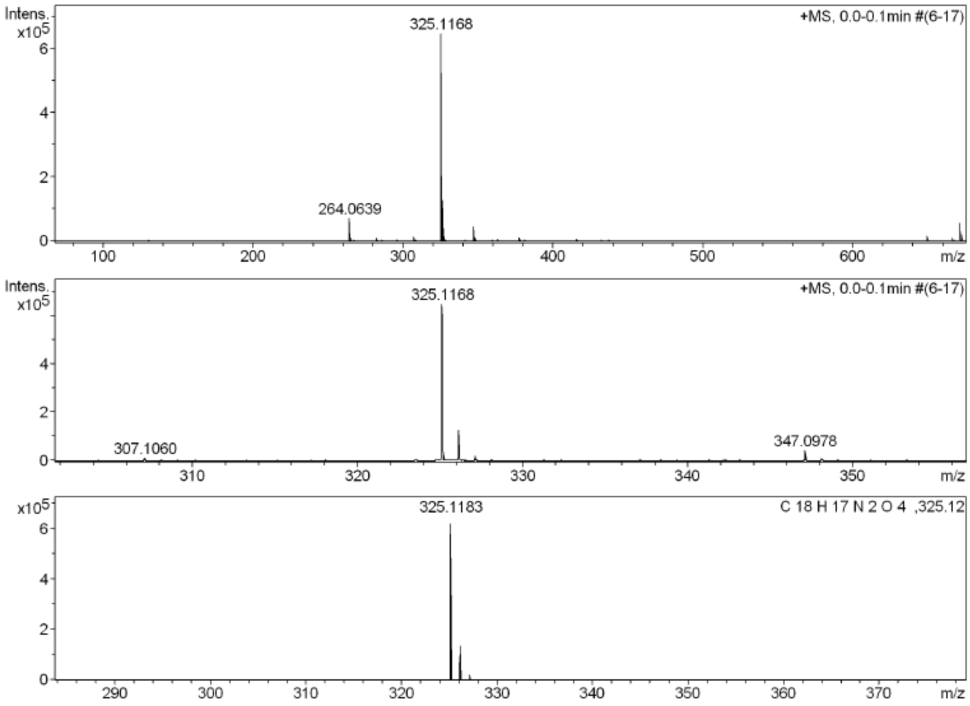

Meas. m/z	#	Formula	m/z	err [ppm]	Mean err [ppm]	rdb	N-Rule	e⁻ Conf
325.1168	1	C 18 H 17 N 2 O 4	325.1183	4.5	4.8	11.5	ok	even

HRESIMS (pos) of compound 3e

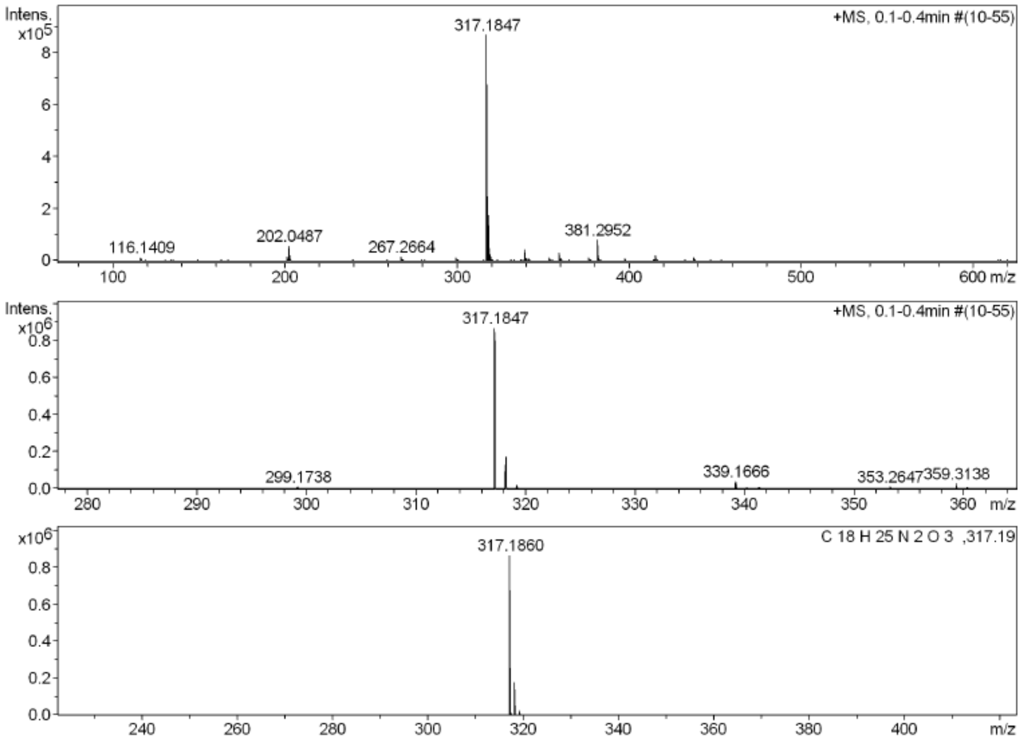

Meas. m/z	#	Formula	m/z	err [ppm]	Mean err [ppm]	rdb	N-Rule	e⁻ Conf
317.1847	1	C 18 H 25 N 2 O 3	317.1860	4.0	4.2	7.5	ok	even

HRESIMS (pos) of compound 3f

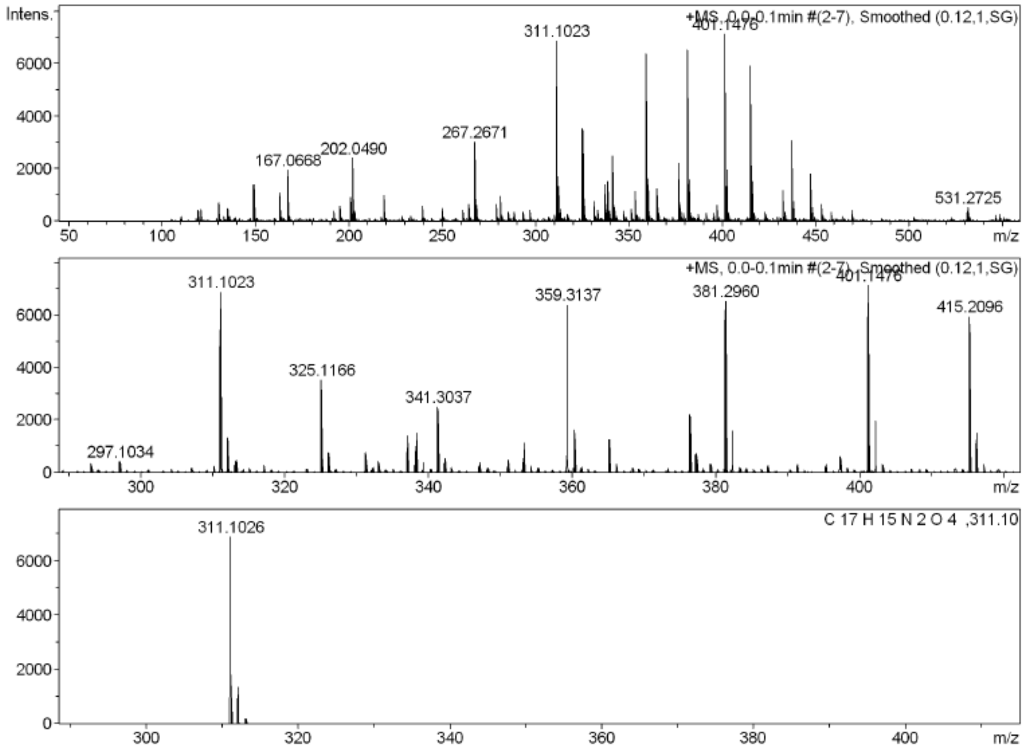

Meas. m/z	#	Formula	m/z	err [ppm]	Mean err [ppm]	rdb	N-Rule	e⁻ Conf
311.1023	1	C 17 H 15 N 2 O 4	311.1026	1.2	0.7	11.5	ok	even

HRESIMS (pos) of compound 3g

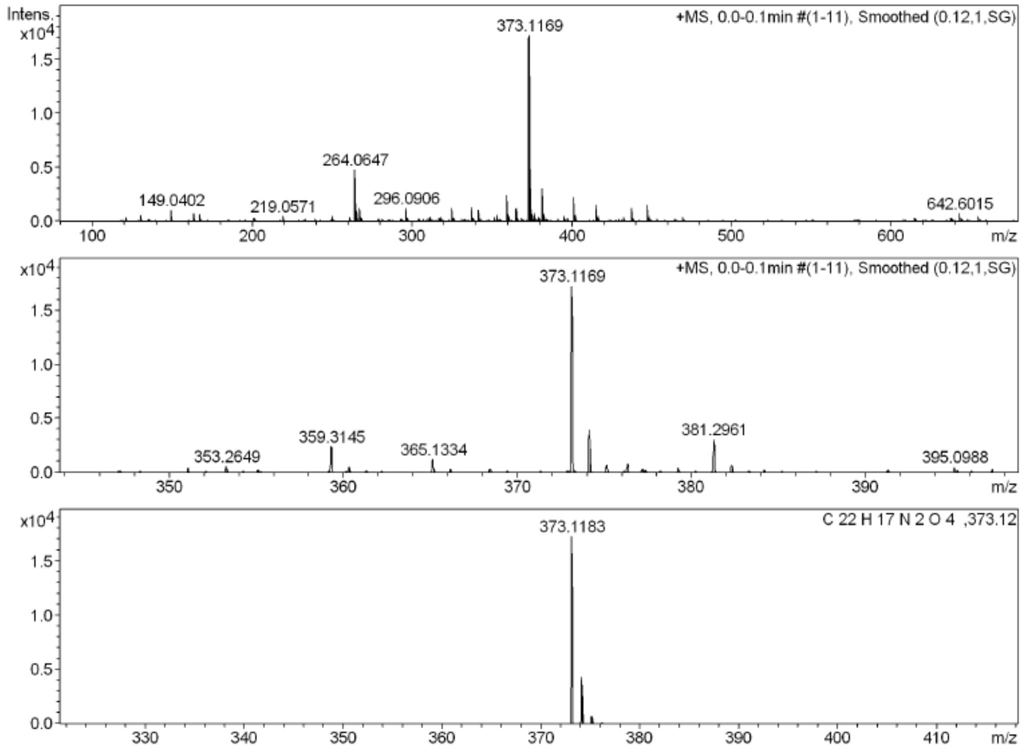

Meas. m/z	#	Formula	m/z	err [ppm]	Mean err [ppm]	rdb	N-Rule	e⁻ Conf
373.1169	1	C 22 H 17 N 2 O 4	373.1183	3.8	3.7	15.5	ok	even

HRESIMS (pos) of compound 3h

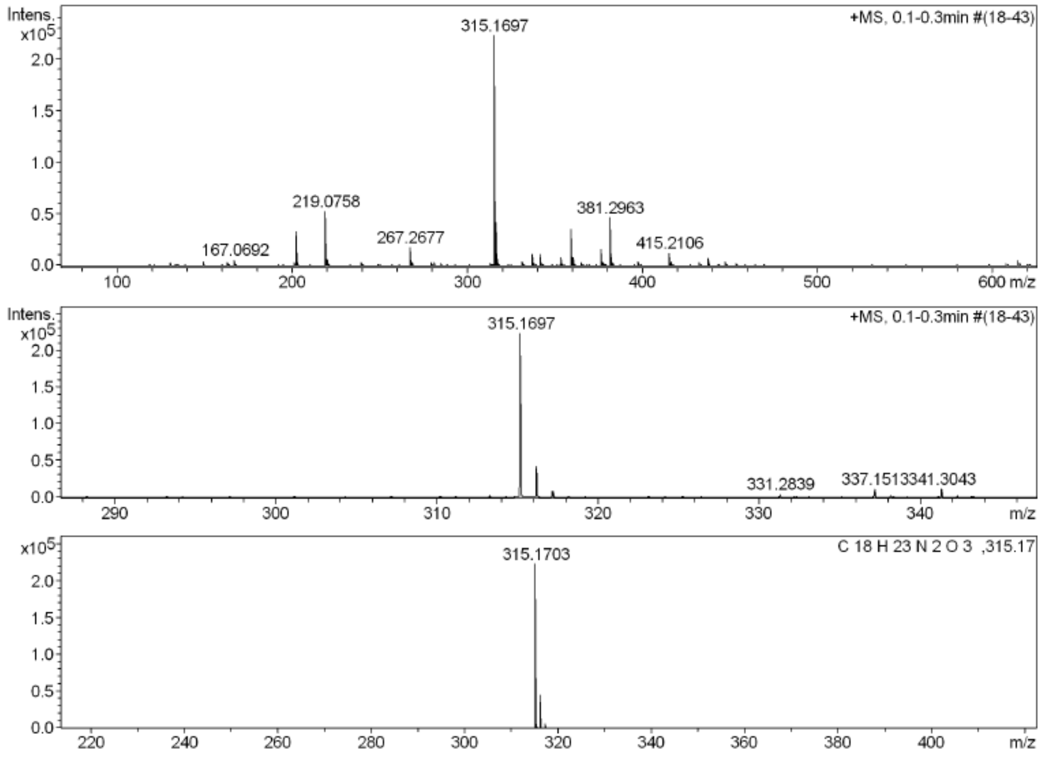

Meas. m/z	#	Formula	m/z	err [ppm]	Mean err [ppm]	rdb	N-Rule	e⁻ Conf
315.1697	1	C 18 H 23 N 2 O 3	315.1703	2.0	1.4	8.5	ok	even

HRESIMS (pos) of compound 3i

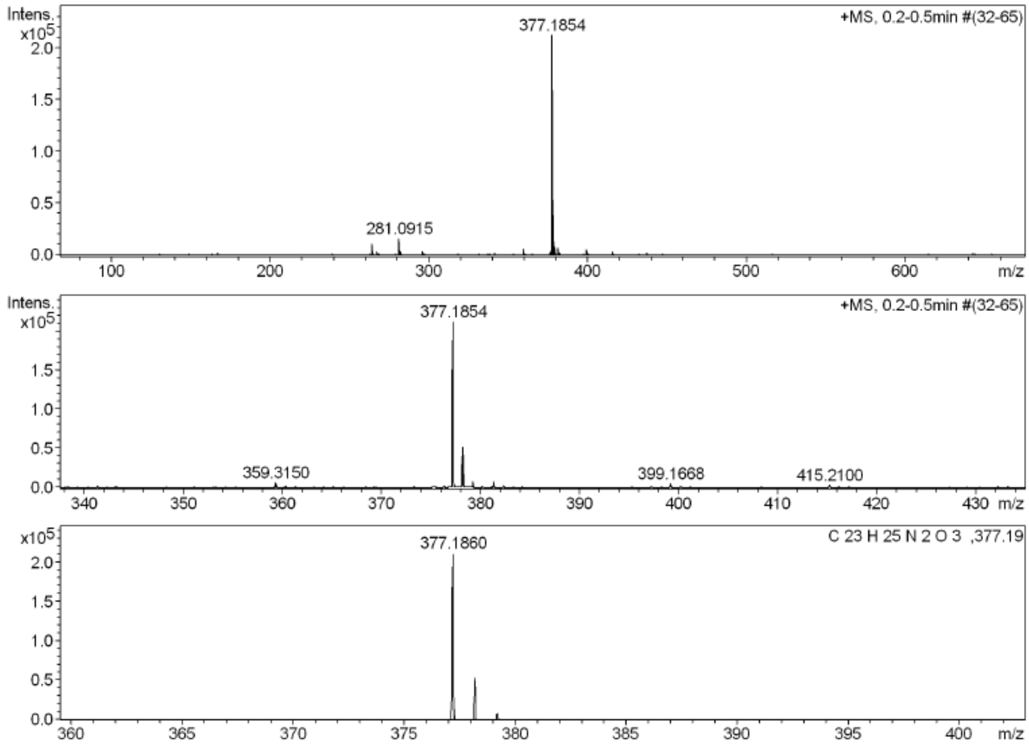

Meas. m/z	#	Formula	m/z	err [ppm]	Mean err [ppm]	rdb	N-Rule	e¯ Conf
377.1854	1	C 23 H 25 N 2 O 3	377.1860	1.5	1.9	12.5	ok	even

HRESIMS (pos) of compound 3j

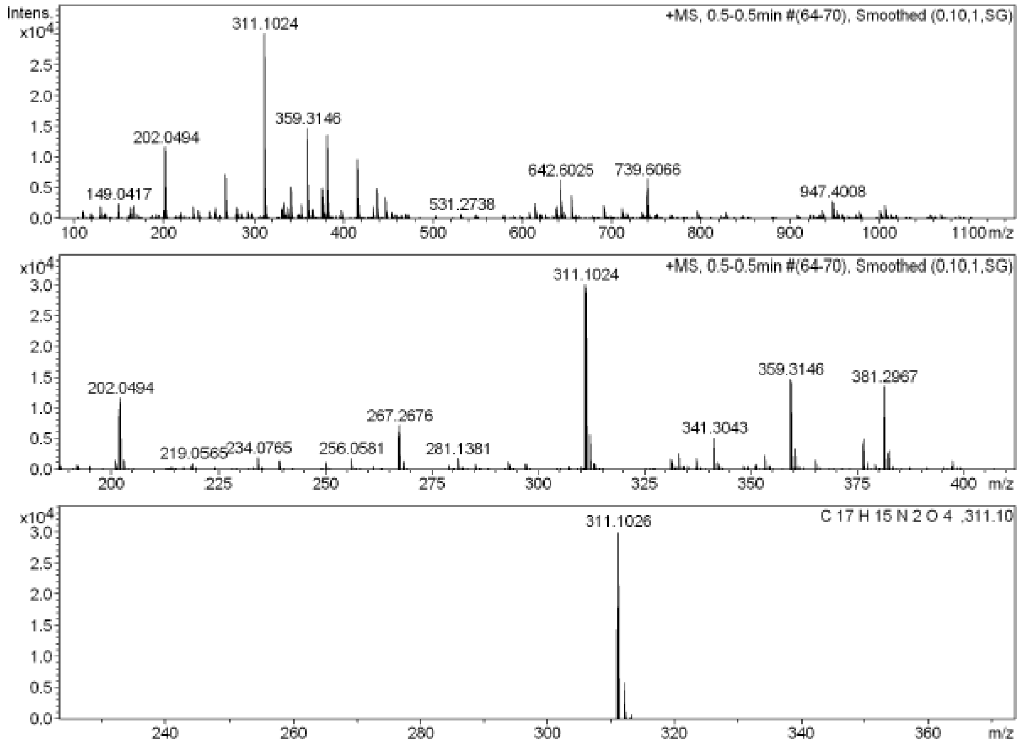

Meas. m/z	#	Formula	m/z	err [ppm]	Mean err [ppm]	rdb	N-Rule	e¯ Conf
311.1024	1	C 17 H 15 N 2 O 4	311.1026	0.7	0.7	11.5	ok	even

HRESIMS (pos) of compound 3k

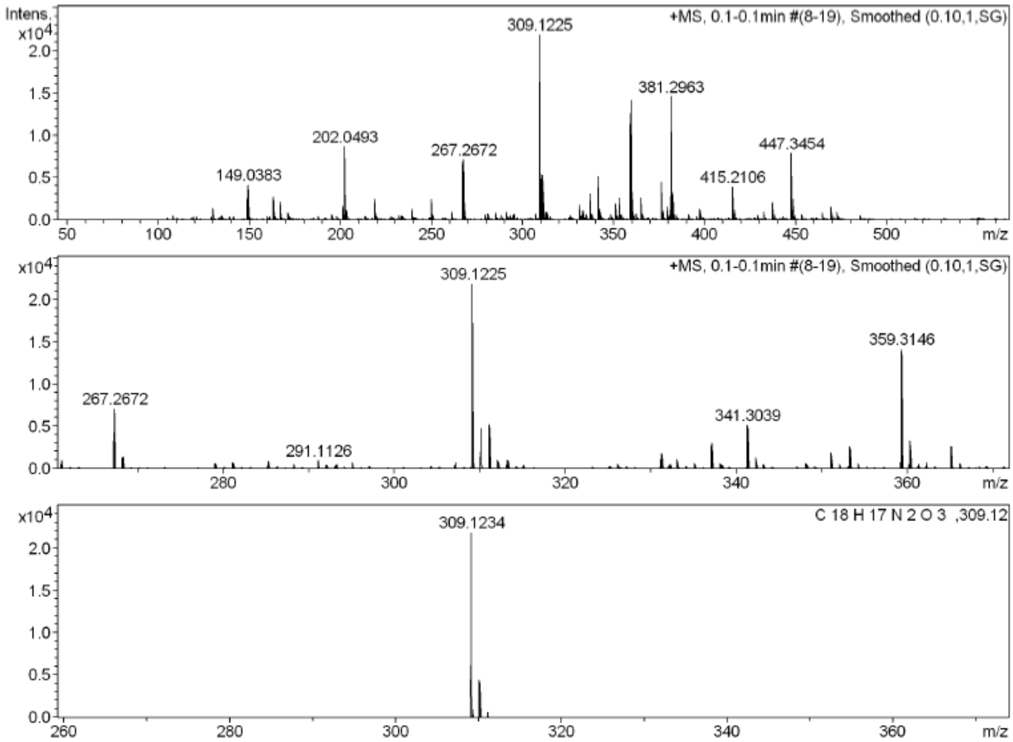

Meas. m/z	#	Formula	m/z	err [ppm]	Mean err [ppm]	rdb	N-Rule	e⁻ Conf
309.1225	1	C 18 H 17 N 2 O 3	309.1234	2.9	11.8	11.5	ok	even

HRESIMS (pos) of compound 3l

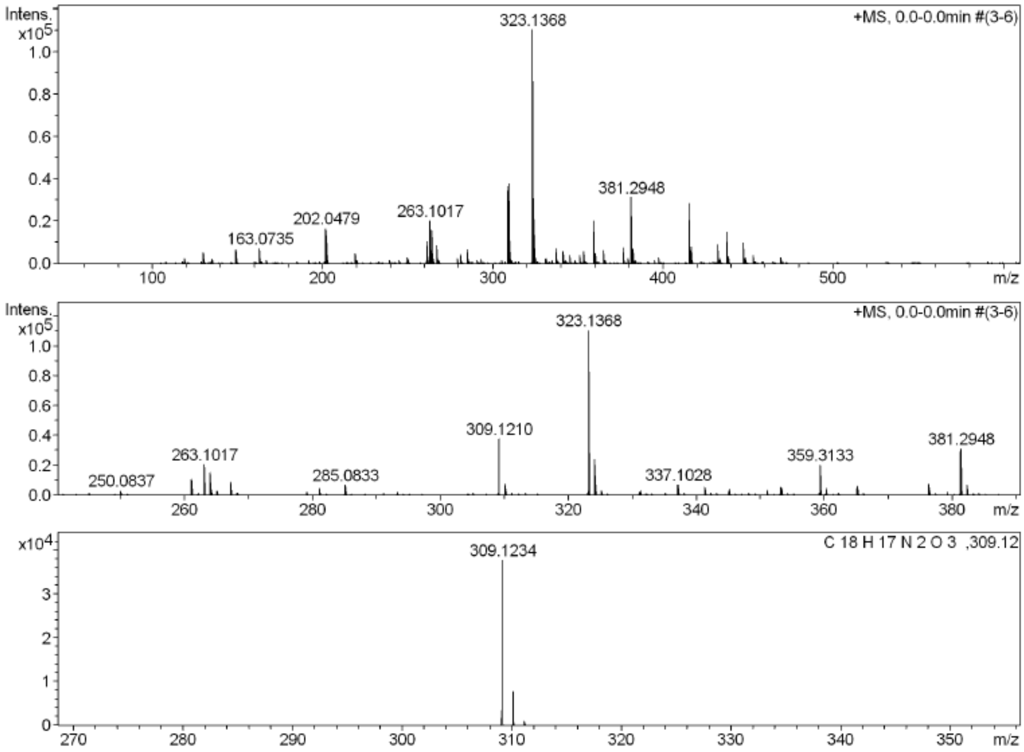

Meas. m/z	#	Formula	m/z	err [ppm]	Mean err [ppm]	rdb	N-Rule	e⁻ Conf
309.1210	1	C 18 H 17 N 2 O 3	309.1234	7.6	7.3	11.5	ok	even

HRESIMS (pos) of compound 3m

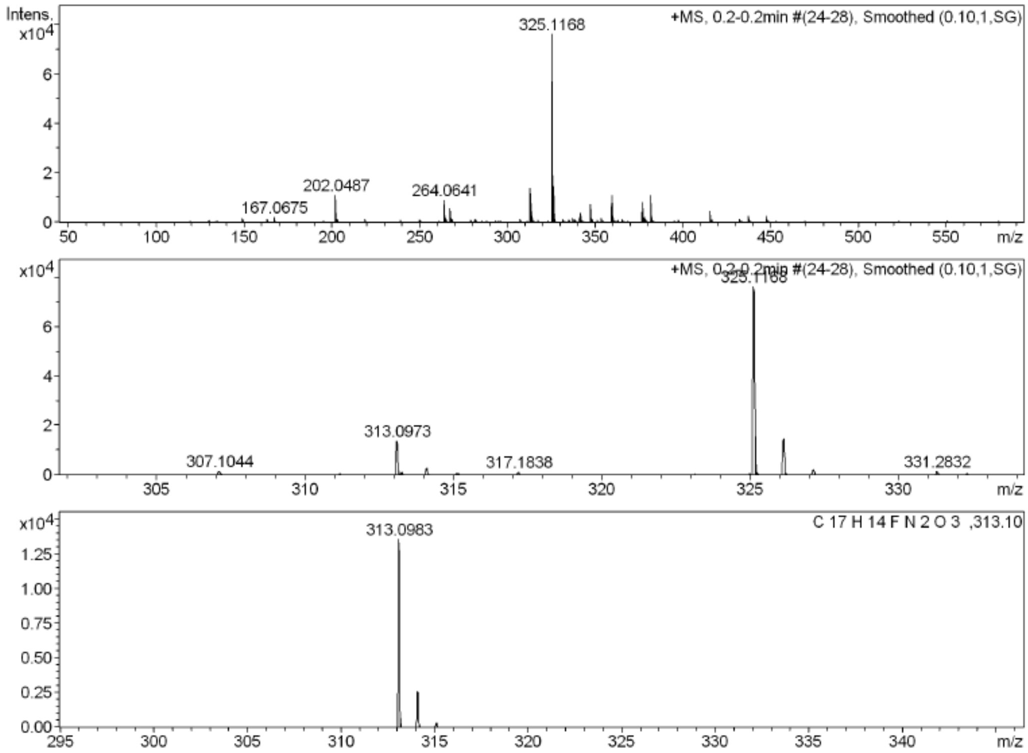

Meas. m/z	#	Formula	m/z	err [ppm]	Mean err [ppm]	rdb	N-Rule	e⁻ Conf
313.0973	1	C 17 H 14 F N 2 O 3	313.0983	3.2	-0.3	11.5	ok	even

HRESIMS (pos) of compound 3n

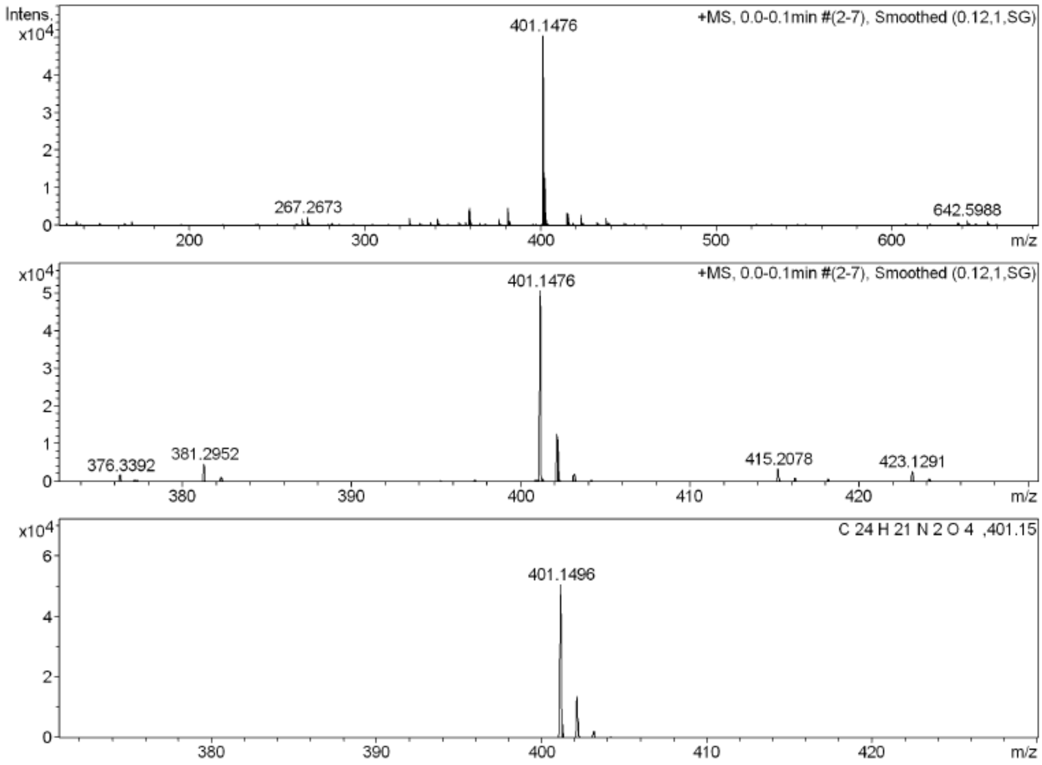

Meas. m/z	#	Formula	m/z	err [ppm]	Mean err [ppm]	rdb	N-Rule	e⁻ Conf
401.1476	1	C 24 H 21 N 2 O 4	401.1496	5.0	5.6	15.5	ok	even

HRESIMS (pos) of compound 3o

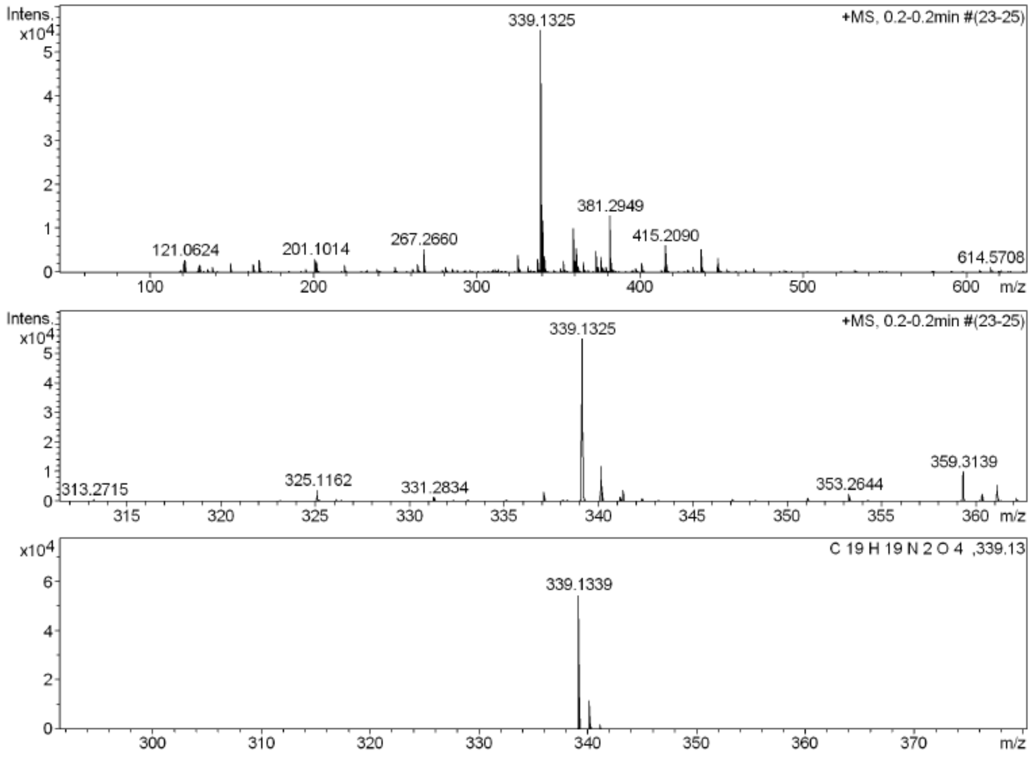

Meas. m/z	#	Formula	m/z	err [ppm]	Mean err [ppm]	rdb	N-Rule	e^- Conf
339.1325	1	C 19 H 19 N 2 O 4	339.1339	4.2	4.7	11.5	ok	even

HRESIMS (pos) of compound 3p

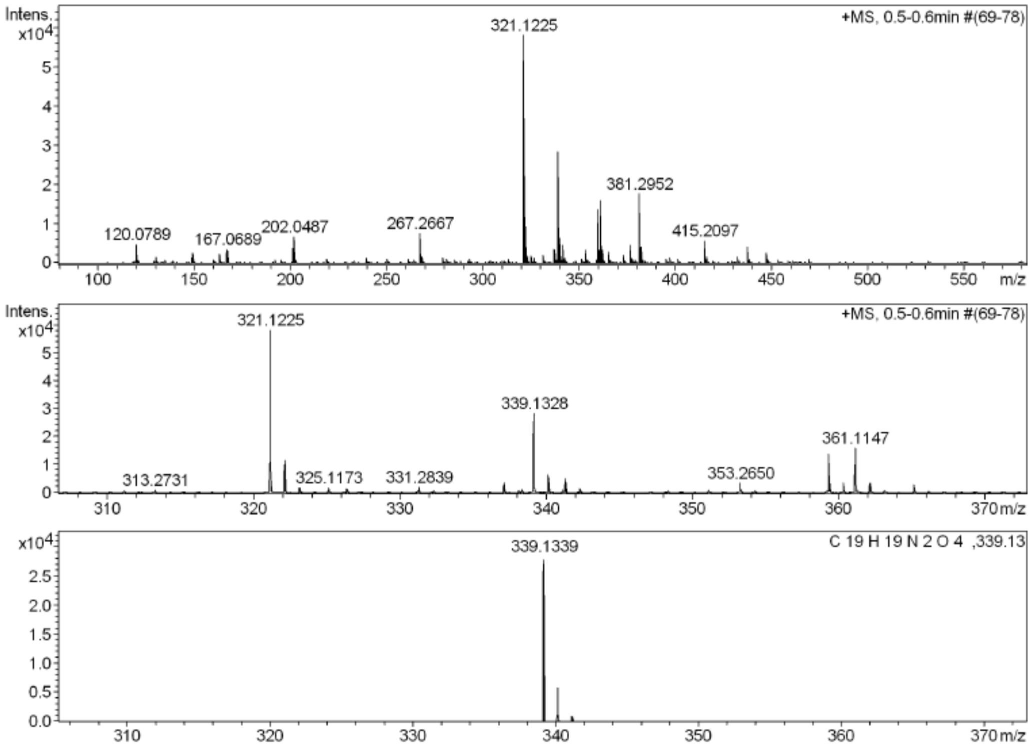

Meas. m/z	#	Formula	m/z	err [ppm]	Mean err [ppm]	rdb	N-Rule	e^- Conf
339.1328	1	C 19 H 19 N 2 O 4	339.1339	3.3	3.9	11.5	ok	even

HRESIMS (pos) of compound 3q

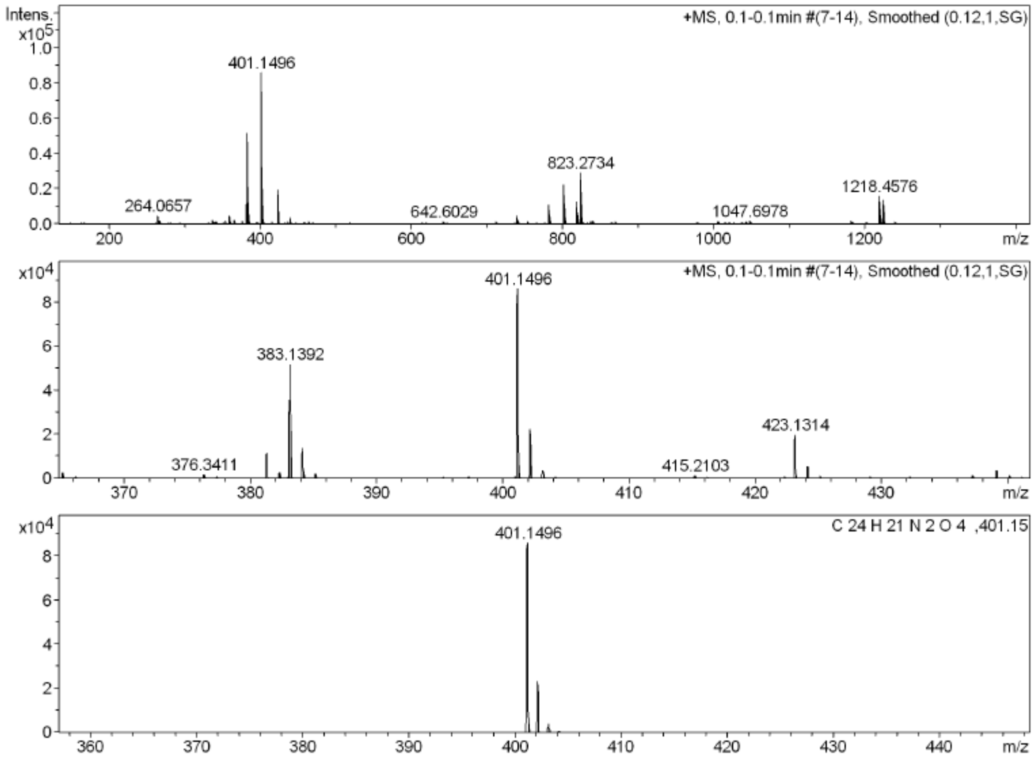

Meas. m/z	#	Formula	m/z	err [ppm]	Mean err [ppm]	rdb	N-Rule	e⁻ Conf
401.1496	1	C 24 H 21 N 2 O 4	401.1496	-0.0	0.7	15.5	ok	even

HRESIMS (pos) of compound 3r

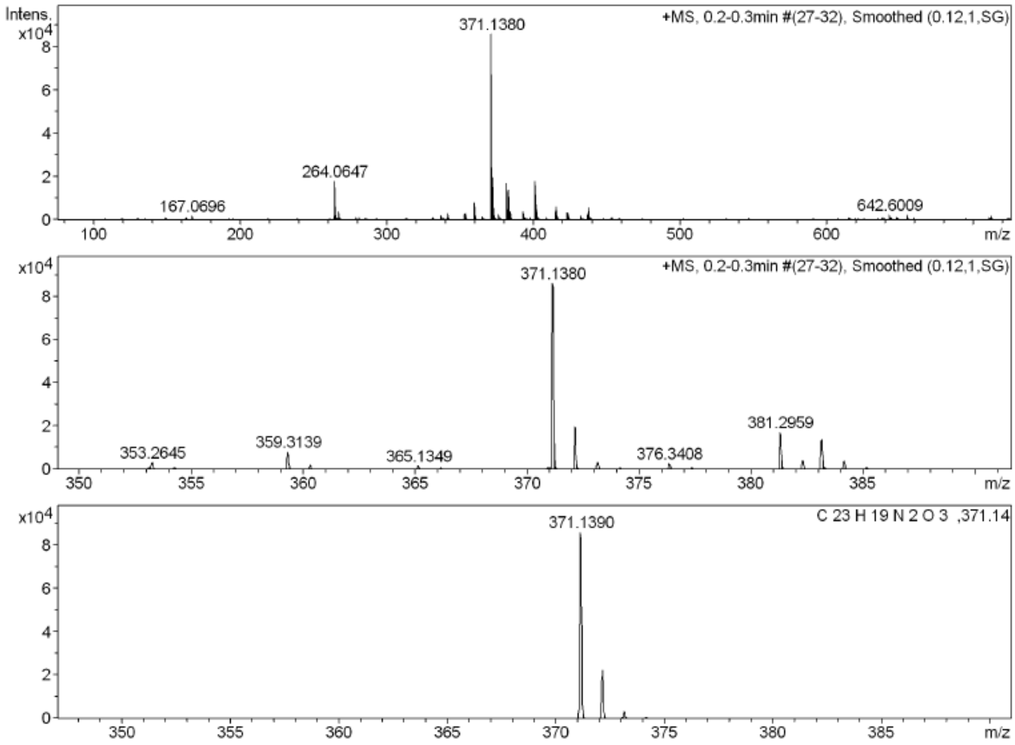

Meas. m/z	#	Formula	m/z	err [ppm]	Mean err [ppm]	rdb	N-Rule	e⁻ Conf
371.1380	1	C 23 H 19 N 2 O 3	371.1390	2.8	3.6	15.5	ok	even

HRESIMS (pos) of compound 3s

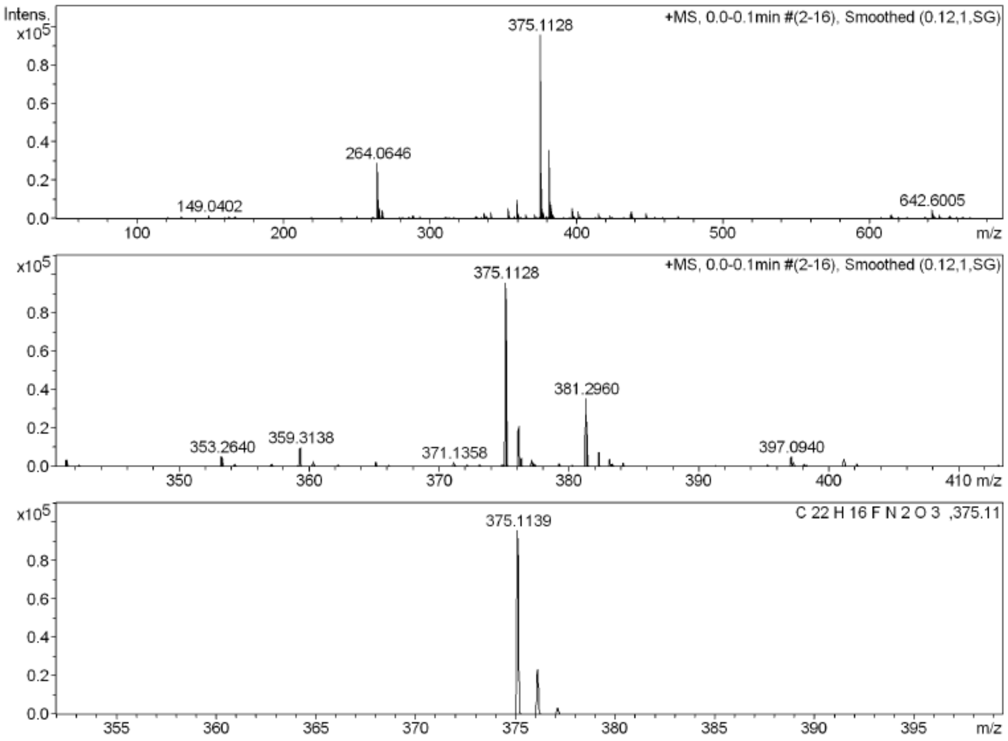

Meas. m/z	#	Formula	m/z	err [ppm]	Mean err [ppm]	rdb	N-Rule	e⁻ Conf
375.1128	1	C 22 H 16 F N 2 O 3	375.1139	3.0	3.4	15.5	ok	even

HRESIMS (pos) of compound 3t

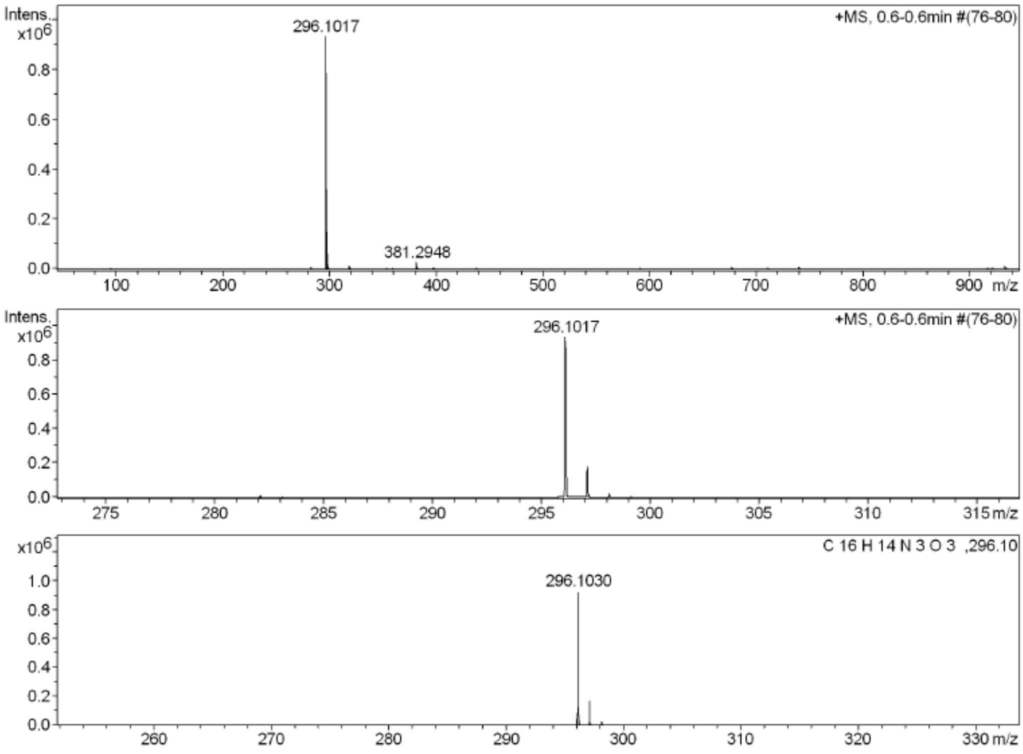

Meas. m/z	#	Formula	m/z	err [ppm]	Mean err [ppm]	rdb	N-Rule	e⁻ Conf
296.1017	1	C 16 H 14 N 3 O 3	296.1030	4.2	4.9	11.5	ok	even

HRESIMS (pos) of compound 3u

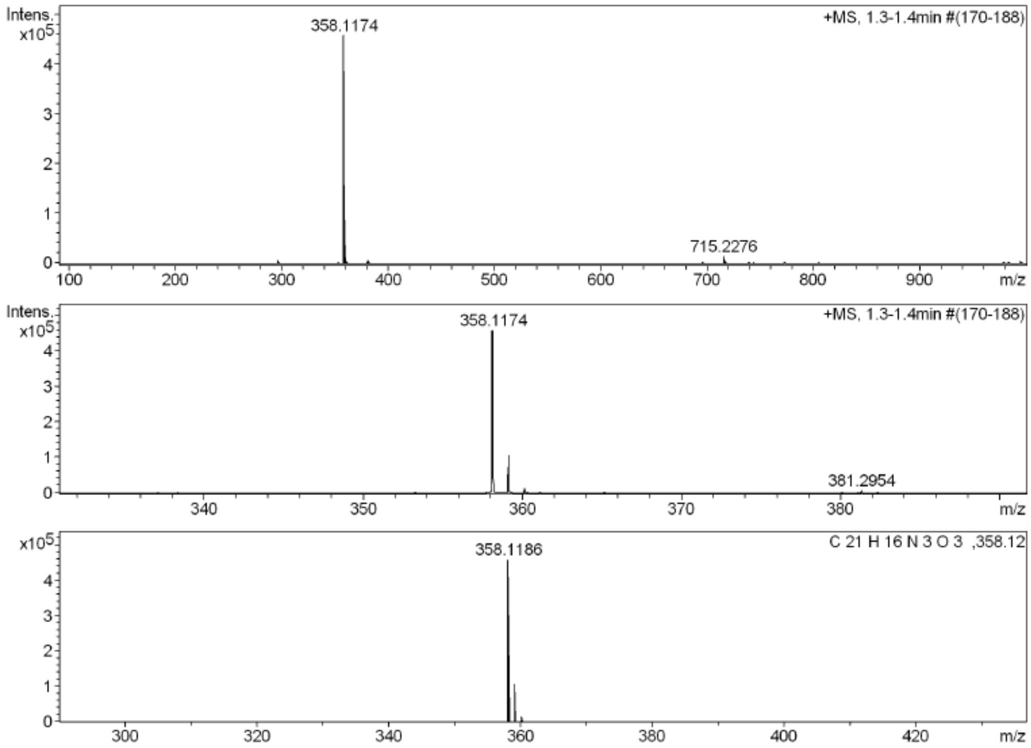

Meas. m/z	#	Formula	m/z	err [ppm]	Mean err [ppm]	rdb	N-Rule	e⁻ Conf
358.1174	1	C 21 H 16 N 3 O 3	358.1186	3.5	3.8	15.5	ok	even

HRESIMS (neg) of compound 11a

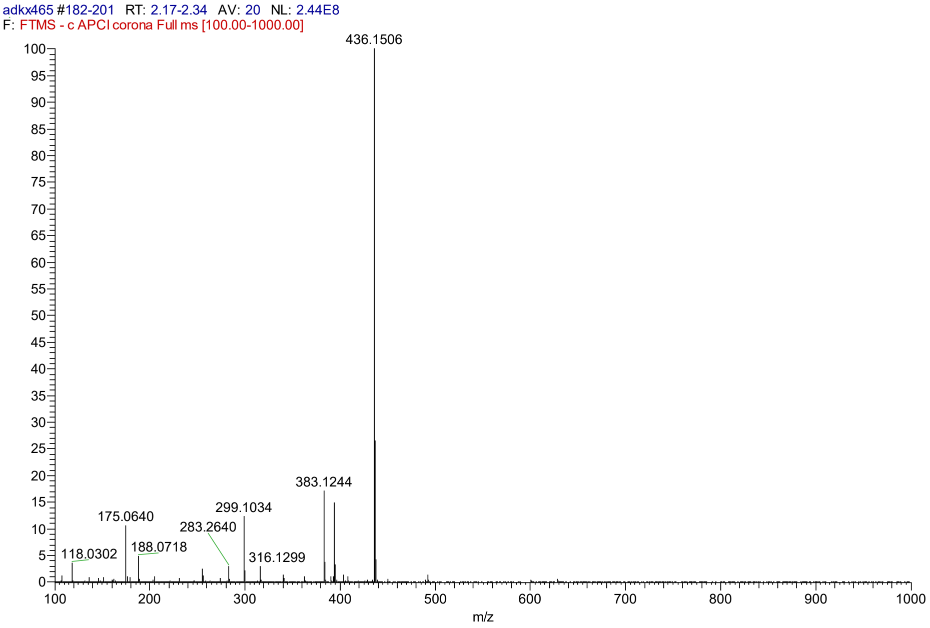

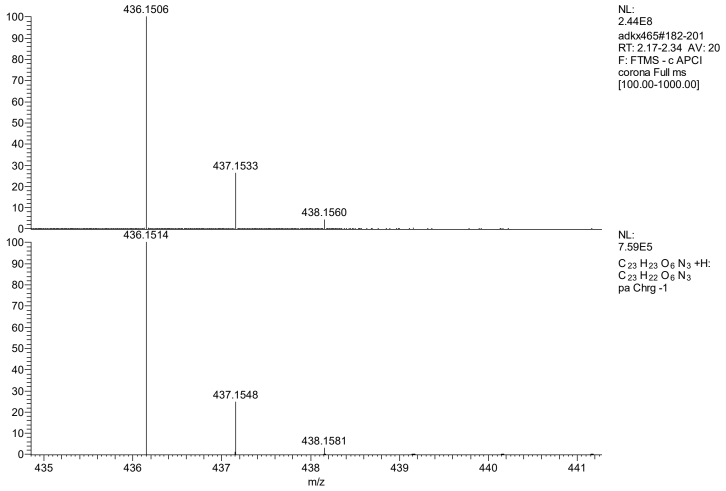

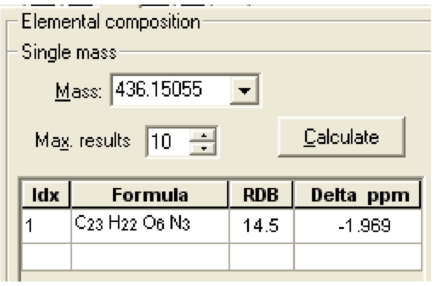

Elemental composition

Single mass

Mass: 436.15055

Max. results 10

Calculate

Idx	Formula	RDB	Delta ppm
1	$C_{23}H_{22}O_6N_3$	14.5	-1.969

HRESIMS (neg) of compound 11b

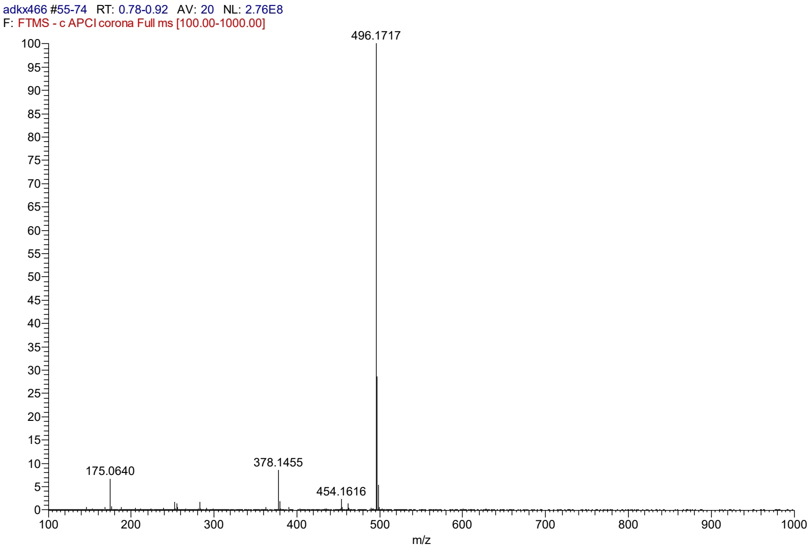

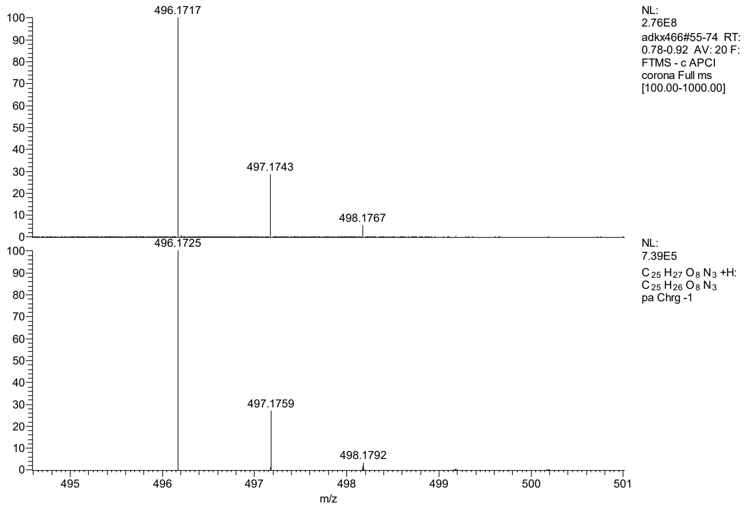

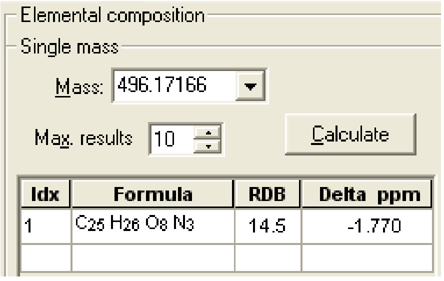

Elemental composition

Single mass

Mass: 496.17166

Max. results 10

Calculate

Idx	Formula	RDB	Delta ppm
1	$C_{25}H_{26}O_8N_3$	14.5	-1.770

HRESIMS (neg) of compound 11c

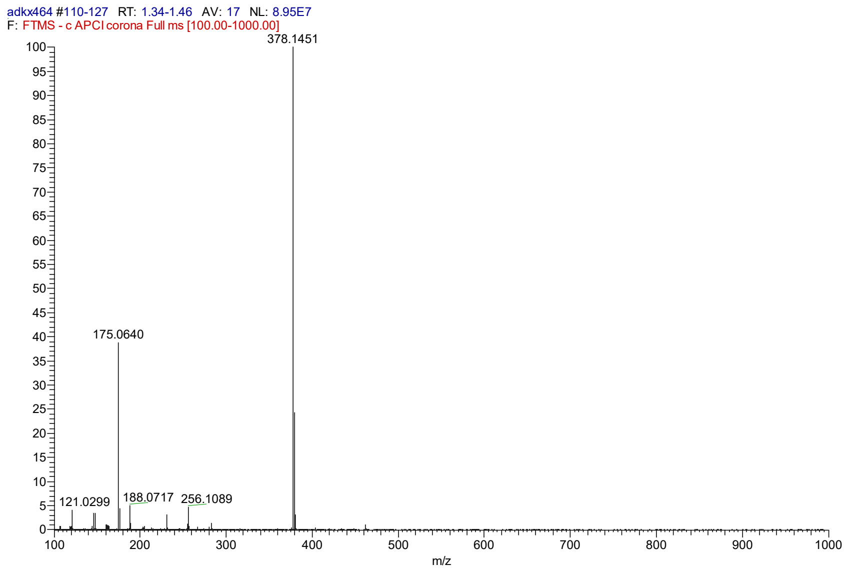

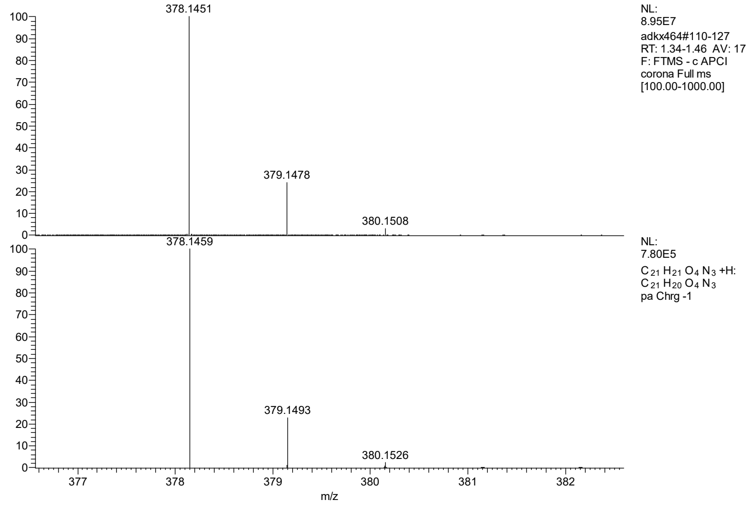

Elemental composition

Single mass

Mass: 378.14505

Max. results 10

Calculate

Idx	Formula	RDB	Delta ppm
1	$C_{21}H_{20}O_4N_3$	13.5	-2.326

HRESIMS (neg) of compound 11d

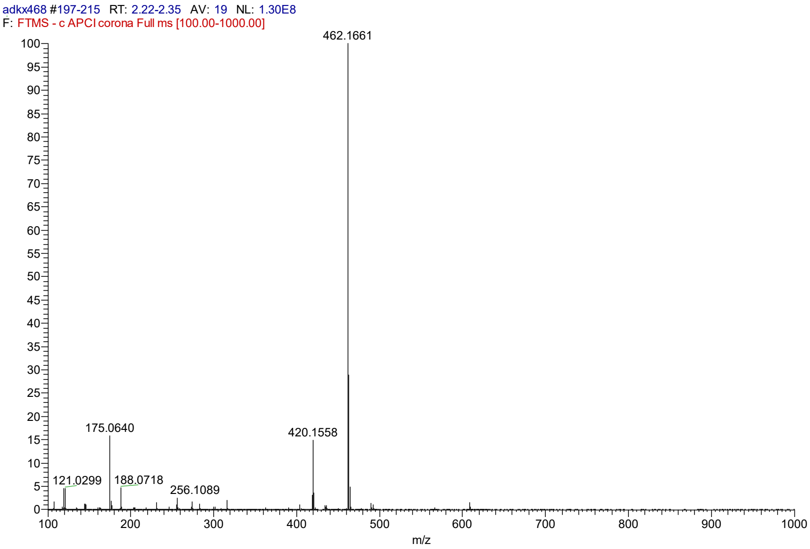

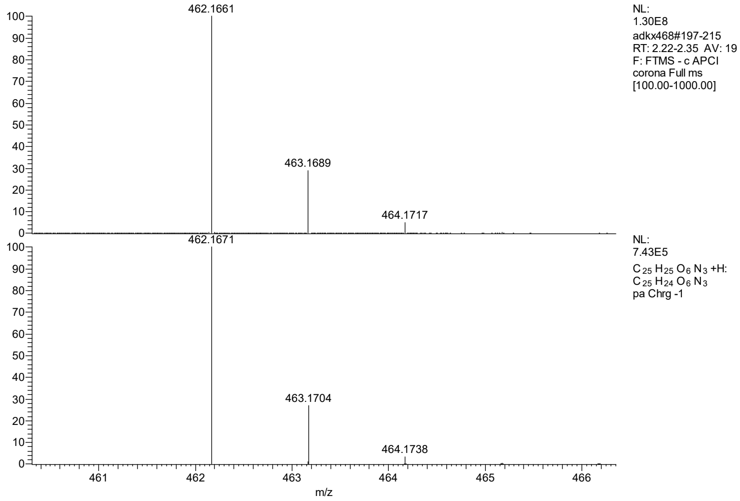

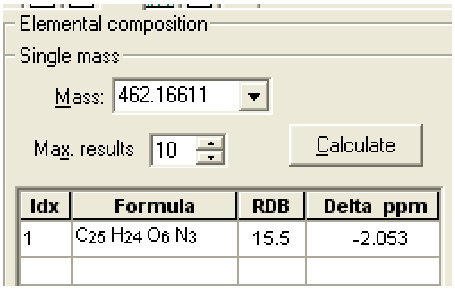

Elemental composition

Single mass

Mass: 462.16611

Max. results 10

Calculate

Idx	Formula	RDB	Delta ppm
1	$C_{25}H_{24}O_6N_3$	15.5	-2.053

HRESIMS (neg) of compound 11e

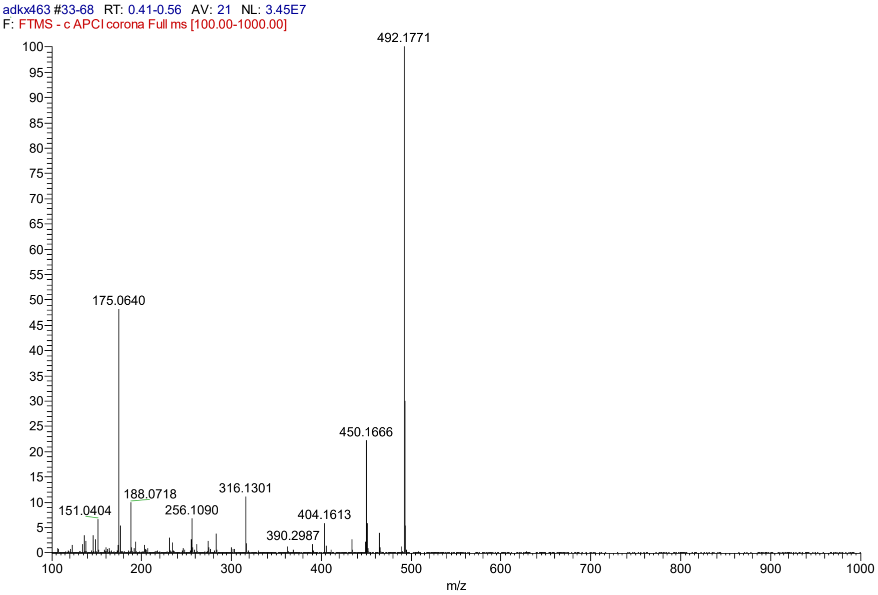

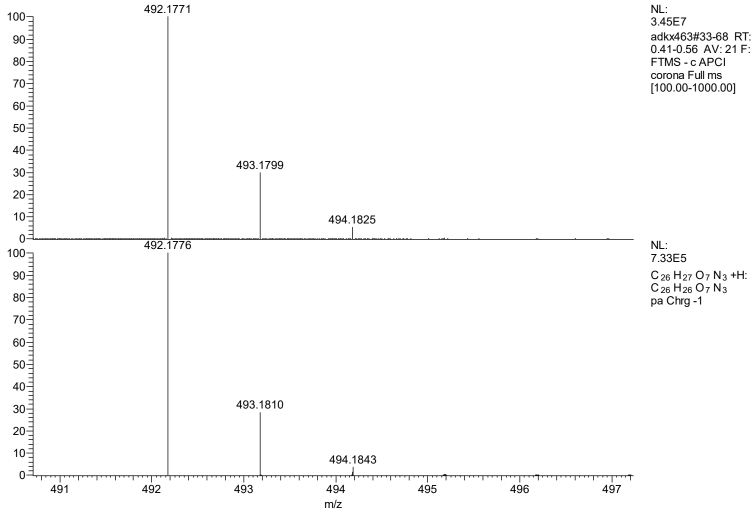

Elemental composition

Single mass

Mass: 492.17709

Max. results 10 Calculate

Idx	Formula	RDB	Delta ppm
1	$C_{26}H_{26}O_7N_3$	15.5	-1.084

HRESIMS (neg) of compound 11f

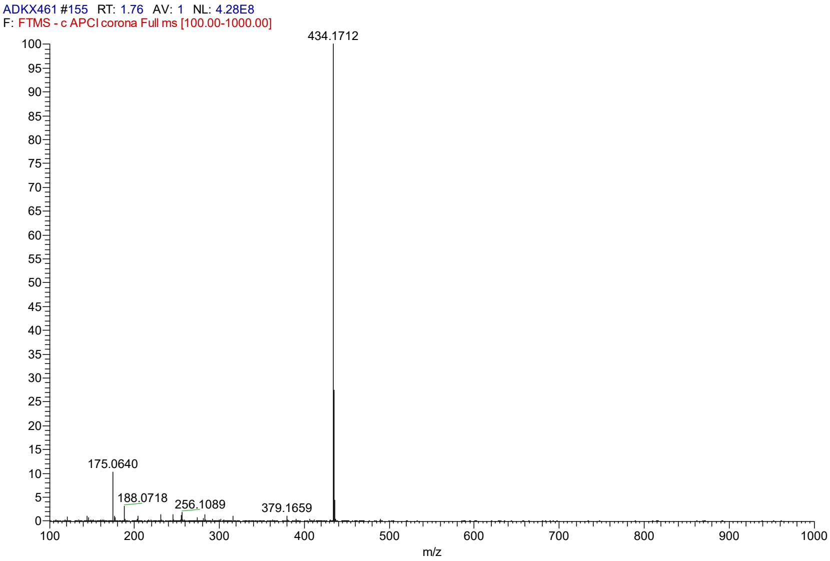

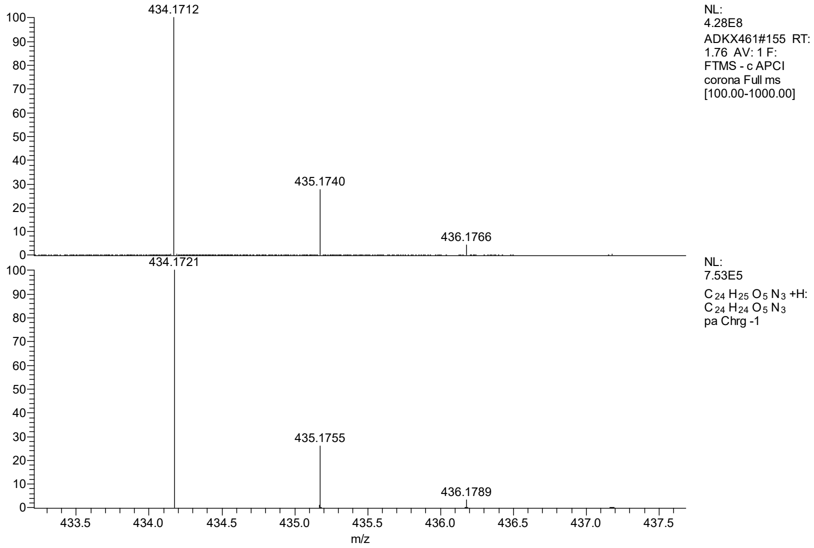

Elemental composition

Single mass

Mass: 434.17123

Max. results 10

Calculate

Idx	Formula	RDB	Delta ppm
1	$C_{24}H_{24}O_5N_3$	14.5	-2.105

HRESIMS (neg) of compound 11g

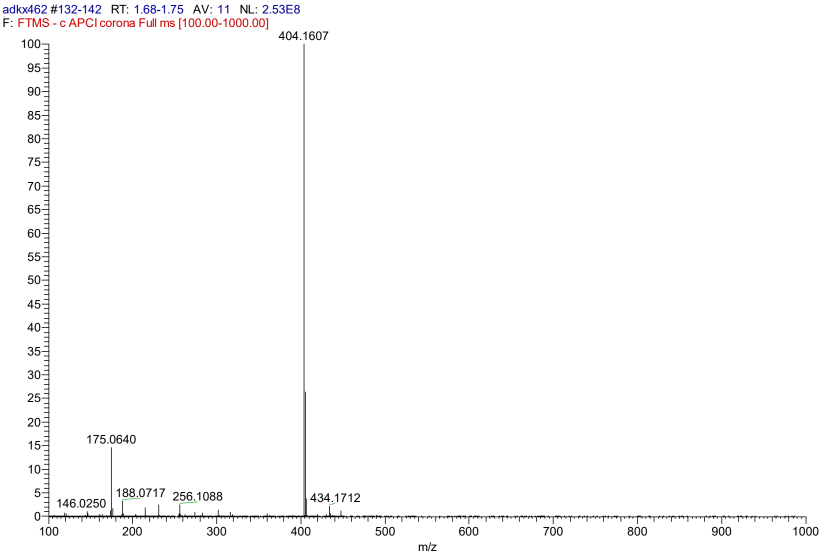

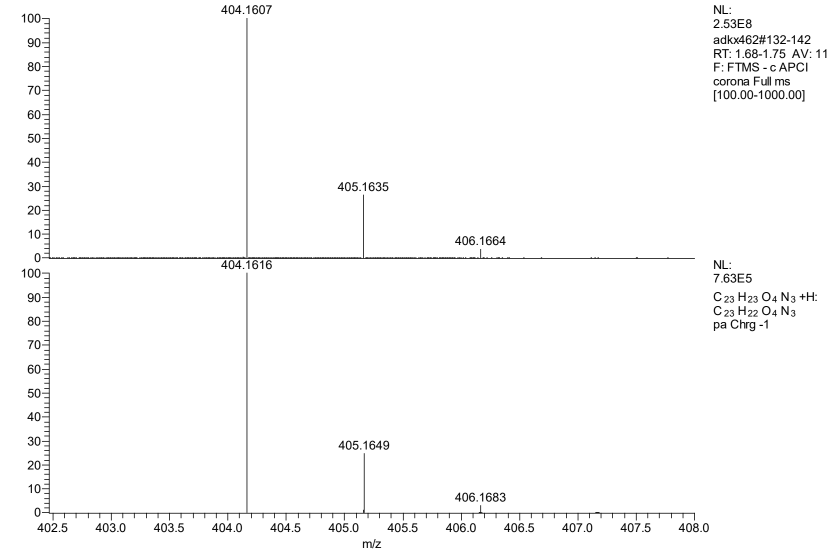

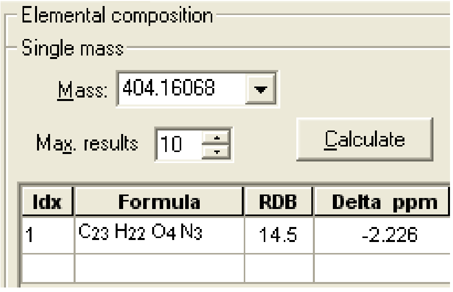

Elemental composition

Single mass

Mass: 404.16068

Max. results 10

Calculate

Idx	Formula	RDB	Delta ppm
1	$C_{23}H_{22}O_4N_3$	14.5	-2.226
